# Acknowledgement to Reviewers of *Sensors* in 2019

**DOI:** 10.3390/s20020576

**Published:** 2020-01-20

**Authors:** Sensors Editorial Office

**Affiliations:** MDPI AG, St. Alban-Anlage 66, 4052 Basel, Switzerland

Peer review is an essential part in the publication process, ensuring that *Sensors* maintains high quality standards for its published papers. In 2019, a total of 5658 papers were published in the journal. Thanks to the cooperation of our reviewers, the median time to first decision was 17 days and the median time to publication was 43 days. Among the reviewers who reviewed for *Sensors* in 2019, the following five have been selected to receive the “*Sensors* 2019 Outstanding Reviewer Award” for the timeliness and quality of their review reports in 2019:

Herrera-May, Agustin L. from Universidad Veracruzana, Mexico
Curiac, Daniel-Ioan from Politehnica University of Timisoara, Romania
Esposito, Flavio from University of Naples “Parthenope”, Italy
Tsiropoulou, Eirini Eleni from University of New Mexico, USA
Moh, Sangman from Chosun University, Korea

The editors also express their sincere gratitude to all of the following, who shared their time and expert opinion by reviewing for *Sensors* in 2019:
Aaen-Stockdale, CraigLiu, ShaohuiAazam, MohammadLiu, ShenghengAbadal, SergiLiu, ShijunAbaid, NicoleLiu, Shing-HongAbalde-Cela, SaraLiu, ShuaiqiAbali, B. EmekLiu, SongtaoAbambres, MiguelLiu, SongzuoAbate, DanteLiu, SuxingAbawajy, JemalLiu, TaoAbbas, SawaidLiu, TianyuAbbasi, HamidLiu, TingyiAbbasi, QammerLiu, Tung-KuanAbbaszadeh, Shiva (Canada)Liu, Tzong-shiAbbaszadeh, Shiva (USA)Liu, WankeAbbene, LeonardoLiu, WeiAbbod, MaysamLiu, WeiboAbdalla, AhmedLiu, Wei-ChengAbdallah, AliLiu, WeihuaAbdelazim, SamehLiu, Wei-RongAbdelaziz Kerrache, ChakerLiu, WeitingAbdeljaber, OsamaLiu, WeiweiAbdelkefi, AbdessattarLiu, WenAbdel-Motaleb, Ibrahim M.Liu, Wen-ChengAbdel-Nasser, MohamedLiu, Weng-KunAbdel-Rahman, EihabLiu, XianhuAbdelsamea, MohamedLiu, XiaoguangAbdolvand, RezaLiu, XinAbdul, RazaqueLiu, XuejunAbdul-Aziz, AliLiu, XunchenAbdulhalim, IbrahimLiu, XuqingAbdulridha, JaafarLiu, YahuiAbedini Nassab, RoozbehLiu, Yan-AnAbegão, LuisLiu, YangAbel, DirkLiu, YangyangAbelmann, LeonLiu, YaolongAbhayasinghe, NimsiriLiu, YeAbir, MuhammadLiu, YiminAbonyi, JanosLiu, YingtaoAbonyi, JánosLiu, YingxiangAbot, JandroLiu, YongbinAbramovich, HaimLiu, YuAbrams, Michael J.Liu, YuanchangAbreu, AntónioLiu, YubaoAbrishambaf, RezaLiu, YufeiAbrol, AmritLiu, YuHaoAbu Ghazaleh, HaithamLiu, YumingAbu Shattal, MohammadLiu, YunAbumazwed, AhmedLiu, ZeliangAbu-Shaban, ZohairLiu, ZenghuaAbu-Siada, AhmedLiu, ZhengchunAbu-Tair, MamunLiu, ZhenguangAbuzneid, Abdel-shakourLiu, ZhentaoAcar, AbbasLiu, ZhiAccardo, DomenicoLiu, ZhiyongAccornero, FedericoLiu, ZhongAceituno, Javier F.Liu, ZhuofuAcerbi, FabioLiu, ZipingAcevedo, Rubén CarlosLiuzzi, GiulianoAcharya, U RajendraLiVoti, RobertoAchim, CristinaLivreri, PatriziaAchterberg, EricLiyanage, MadhusankaAdachi, YoshiakiLiyanapathirana, RanjithAdam, VojtechLizal, FrantisekAdamczyk, MarcinLizcano, DavidAdami, DavideLlamas-Nistal, MartínAdamiec, EwaLlaria, AlvaroAdamou Abba Ari, AdoLlera, MiguelAdams, Matthew DLlobet, EduardAdcock, ChristopherLloret, JaimeAddy, Partha SarathiLo Brutto, MauroAdegoke, OluwasesanLo, Kuang YaoAdeloju, SamuelLo, Shi-WeiAderohunmu, FemiLo, Wai LunAdhikari, Bal RamLobato-Morales, HumbertoAdhikari, KaushallyaLobo-Castañón, María JesúsAdly, NouranLobov, SergeyAdriaensen, StefanLochocki, BenjaminAdu-Gyamfi, YawŁodyga-Chruścińska, ElżbietaAfanasyev, IlyaLogozzo, SilviaAfefy, Hamdy M.Logsdon, SallyAffanni, AntonioLohr, ChristopheAfonso, ManyaLohstroh, AnnikaAfzal, Muhammad UsmanLokhorst, KeesAgafonov, VadimLolla, DineshAgarwal, AnkitLomas-Vega, RafaelAgarwal, KushLombardi, FedericoAgarwal, Nisha RaniLombardi, MaraAgarwal, RachitLombardo, LucaAgathos, AlexanderLong, BingAgati, GiovanniLong, ChengAggelis, Dimitrios G.Long, ChengjiangAggogeri, FrancescoLong, Jed A.Aggrey, JohnLong, TengfeiAghababaee, HosseinLong, XiAghajarian, MickaelLongo, DomenicoAghakhani, AmirrezaLongo, FrancescoAghayi, EmadLopes, Ana C.Aglyamov, SalavatLopes, Daniel SimõesAgrafiotis, IoannisLópez Antón, RicardoAgranov, GennadiyLópez Espí, Pablo LuisAgrawal, AnkitLópez Granado, Otoniel MarioAgrawal, RajeevLópez Lago, ElenaAgrawal, RakshitLópez Ruiz, NuriaAgresti, JuriLópez, AinaraAguasca-Colomo, RicardoLópez, AlejandroAgudo, AntonioLópez, Antonio M.Agüera-Vega, FranciscoLópez, JoaquínAguilar, LeocundoLópez, MaríaAguilar-Caballos, María PazLópez, Miguel ÁngelAguilar-González, AbielLopez-Cruz, IrineoAguilera, Cristhian A.López-Estrada, Francisco RonayAguilera, TeodoroLopez-Fernandez, JorgeAguilos, MaricarLopez-Fernandez, XoseAguinaga, IkerLópez-Gil, Juan-MiguelAhamed, NizamLopez-Higuera, JoseAhamed, Nizam UddinLópez-Huerta, FranciscoAhi, KiarashLopez-Iturri, PeioAhmad, AdeelLópez-Martínez, AlbertoAhmad, MuhammadLópez-Nava, Irvin HusseinAhmad, TanveerLópez-Nicolás, GonzaloAhmadivand, ArashLopez-Nuñez, RafaelAhmed, FaridLopez-Otero, PaulaAhmed, MohamedLópez-Sastre, Roberto JavierAhmed, MohiuddinLopomo, NicolaAhmed, QadeerLoprencipe, GiuseppeAhmed, QasimLoranger, SébastienAhmed, RajibLorences-Riesgo, AbelAhmed, RiazLorenzo-Trueba, JorgeAhmed, Syed HassanLoreti, PierpaoloAhn, HosangLorincz, JosipAhn, Jae-HyunLorussi, FedericoAhsan, Md. ShamimLosada-Perez, PatriciaAhuett Garza, HoracioLoskot, PavelAi, TinghuaLosson, EtienneAicardi, IreneLötters, JoostAiello, OrazioLou, ZhichaoAinsbury, LizLoukopoulos, ThanasisAkabane, Ademar T.Loureiro, PauloAkai, NaokiLoures, LuisAkansel, CosgunLouro, PaulaAkasiadis, CharilaosLoutas, TheodorosAkbar, FatemaLovas, TamasAkbar, SheikhLovisolo, LisandroAkca, B. ImranLow, EicherAkhavian, RezaLowe, KelseyAkhtar, ZahidLowe, ShehanAkira, HafukaLoya, J.A.Akitsu, TomokoLoyola-González, OctavioAkkaya, KemalLozano, JesusAkkaynak, DeryaLozano-Guerrero, Antonio JoséAksanli, BarisLu, BingAktas, Yasemin D.Lu, BoAl Machot, FadiLu, ChaoAl Sadsoon, MohammedLu, ChiahaoAlam, FakhrulLu, Chia-JungAlam, TauhidulLu, Dan-FengAlamaniotis, MiltiadisLu, DebiaoAl-Amri, MohammadLu, FeiAlapetite, AlexandreLu, FengAlappattu, Denny P.Lu, KelinAl-Aqrabi, HussainLu, Michael S.-C.Alazzam, AnasLu, MingyangAlba-Flores, RocioLu, MingzhouAlbakri, MohammadLu, MinjiaoAlbarracin, RicardoLu, QikaiAl-Bayati, AliLu, RongxingAlbella, PabloLu, RuochenAlbéri, MatteoLu, ShanAlbertini, Bruno De CarvalhoLu, ShuangAlberto, NéliaLu, SiliangAlbiol, AlbertoLu, TaoAlbu, FelixLu, WeidangAlbuquerque, DanielLu, WenlongAlbuquerque, Daniel FilipeLu, XinAlbuquerque, Robson De OliveiraLu, YanAlbuquerque, VictorLu, YiAlcântara, EnnerLu, YiliAlcaraz, CristinaLu, YuAlcarria, RamonLU, YuzhenAldaya, IvanLu, ZhaomingAldea, EmanuelLu, ZhiquAldrigo, MartinoLuan, EnxiaoAlegre, ThiagoLubczonek, JacekAleixandre, ManuelLuc, JéromeAlejo Eleuterio, RobertoLucas-Estañ, Maria Del CarmenAlejos, Ana VázquezLucev Vasic, ZeljkaAleksander, MucLucian Grigorie, TeodorAleksandrova, MariyaLucklum, RalfAlemayehu, Fisseha M.Luckner, MarcinAlencar, Marcelo Sampaio DeŁuczak, SergiuszAlendal, GuttormLudwig, FrankAlessi, AntoninoLudwinek, KrzysztofAletta, Francesco (Italy)Lugoda, PasinduAletta, Francesco (UK)Luh, Jer-JunnAlevras, PanagiotisLui, FaustaAlexandridis, ThomasLuis Contreras-Vidal, JoseAlexandropoulos, GeorgeLuiz, Alfredo José BarretoAlexandrov, SergeyLukač, NikoAlexiev, KirilLukomska-Szymanska, MonikaAlfano, BrigidaŁukowski, MateuszAlfian, GanjarLumentut, MikailAlgarín, Carlos RoblesLumentut, Mikail F.Algergawy, AlsayedLuna Moreno, DonatoAlgorri, José FranciscoLuna-Perejón, FranciscoAlhashimi, AnasLuo, CaiAl-Hussein, AbdullahLuo, ChangtongAli, G. G. Md. NawazLuo, ChengwenAli, HaiderLuo, GuojieAli, M. MahmoodLuo, HanjiangAli, MonsurLuo, JerryAli, MurtazaLuo, JingtingAli, NourLuo, JuanAli, RashidLuo, LihuiAlías Pujol, FrancescLuo, NingqiAlibakhshikenari, MohammadLuo, ShaAliev, KhurshidLuo, ShanAlisaac, EliasLuo, SidaAl-Jumeily, DhiyaLuo, TonyAlkhodary, MohammadLuo, WenhanAllam, ZaheerLuo, WenjieALLARD, BrunoLuo, XiangangAllen, ChristianLuo, YanhuaAlleysson, DavidLuo, YeAllken, VaneedaLupi, CyrilAllotta, BenedettoLupu, NicoletaAlluri, Nagamalleswara RaoLuque, AmaliaAlmeida Ferreira, HugoLuque, AntonioAlmeida Junior, Jose HumbertoLuque-Nieto, Miguel AngelAlmeida, AitorLuque-Nieto, Miguel-AngelAlmeida, Joao EmilioLurz, FabianAlmeida, JoseLuštrek, MitjaAlmeida, ThiagoLuttgen, AndreaAlmenárez, FlorinaLuvisotto, MicheleAl-Naji, AliLv, ChenAl-Neshawy, FahimLv, HaoAlonso, Alvaro HernandezLv, ZhaoAlonso, J. ArturoLv, ZhihuAlonso, Ricardo S.Lvova, LarisaAlonso, SerafínLyakhov, AndreyAloqaily, MoayadLye, Sun WohAl-Saadi, SaadLynn, Nicholas ScottAlshamaa, DanielLyons, AshleyAlshammari, Majid R.Lyu, Hong-KunAlsharif, Mohammed H.Lyu, HongmingAlsharoa, AhmadLyu, YongqiangAlsina-Pagès, Rosa MaM. Dakka, SamAlskaif, TarekM. Komerath, NarayananAlt Murphy, MargitMA, Andy J.Altaf, AmirMa, BingpengAltammar, HussainMa, ChengAltintas, ZeynepMa, ChristinaAl-Turjman, FadiMa, Christina Zong-HaoAluigi, LucaMa, CongcongAlvarez Alvarez, Juan CarlosMa, DandanÁlvarez López, YuriMa, DaolinAlvarez, DiegoMa, Der-MingÁlvarez, FedericoMa, GuomingÁlvarez, FernandoMa, Guo-mingAlvarez, MarMa, HanbinÁlvarez, MarMa, HongjieAlvarez, RafaelMa, JiajuAlvarez-Bermejo, Jose AntonioMa, JianguoÁlvarez-Tamayo, Ricardo I.Ma, JiayiAlves, Ana Cristina OliveiraMa, JieAlves, José CarlosMa, LeiAlves, Luis NeroMa, LihongAlves, MárioMa, LijiaAlwis, LourdesMa, MengchaoAl-Yaari, AmenMa, MingAmadori, Pierluigi VitoMa, MingshengAman, Muhammad NaveedMa, TingguangAmanatiadis, StamatiosMa, WeiAmancio, DiegoMa, XiaoleiAmani, MeisamMa, XiaolinAmano, KinjiroMa, XignpoAmaris, HortensiaMa, XikuiAmaro, José PedroMa, XinAmatya, SurajMa, YinjiAmbros, JiříMa, Yu TakAmbrožič, TomažMa, Yuan-RonAmbrozinski, LukaszMa, YufeiAmelard, RobertMa, YuqiangAmezquita Sanchez, Juan PabloMa, ZhenliangAmezquita-Sánchez, Juan PabloMa, Zhenqiang (Jack)Amezquita-Semprun, KendrickMa, ZhuoAmin, FarhanMaalek, RezaAmin, M. JunaidMabrouk, RostomAmini, M. HadiMabrouk, SamerAmirian, MohammadrezaMacedo, Carina Regina DeAmirov, AbdulkarimMacedo, EloisaAmmer, KurtMacedo, LuísAmo, DanielMacGregor, Duncan JAmpatzidis, DimitriosMach, PavelAmpatzidis, YiannisMachacek, ZdenekAms, MartinMachado, IvanAmsaad, Fathi Hassan MohamedMachado, MiguelAmundarain, AiertMachado, RenatoAmundsen Huffmaster, SommerMachaj, JurajAn, BeongkuMachida, FumioAn, Jae-SungMachikhin, Alexander S.An, SangminMachwitz, MiriamAnagnostopoulos, GeorgiosMacías, Elsa M.Anagnostopoulos, MariosMaciejewska, MonikaAnagnostopoulos, TheodorosMaciuk, KamilAnand, VijayakumarMačiulienė, MonikaAnanthakrishnan, Soundaram JeevarathinamMackay, RuthAnantrasirichai, NantheeraMackiewicz, MichalAnashkina, ElenaMacko, DominikAnastasova, SalitzaMacnae, James C.Anastassopoulos, VassilisMadamopoulos, NicholasAnbarjafari, GholamrezaMadanayake, ArjunaAncillao, AndreaMadanian, SamanehAncin-Murguzur, Francisco JavierMadeira, Rui NevesAncuti, CosminMadeira, SergioAndersen, Henning BojeMademlis, IoannisAndersen, Jens ChristianMadokoro, HirokazuAnderson, StuartMadruga, Francisco J.Andersson, BengtMadurapperuma, BuddhikaAndersson, MagnusMaehle, ErikAndo Junior, Oswaldo HideoMaeng, SunglyulAndo, NoriyasuMaestre Vidal, JorgeAndoga, RudolfMaestre, RafaelAndrade, Adriano De OliveiraMaga, DusanAndrade, CarlosMagarini, MaurizioAndrade, Francisco JavierMagee, JohnAndré, HugoMagenes, GiovanniAndreaus, UgoMaglaveras, NicosAndreone, AntonelloMagna, GabrieleAndreoni, GiuseppeMagne, SylvainAndretta, MassimoMagnusson, MartinAndrew, NGO Chun YongMagoni, DamienAndrew, Trisha L.Maguire, MarcAndrews, Aaron MaxwellMahajan, RuhiAndrey Andreevich, GolovanMahapatra, PoojaAndrianov, NikolaiMahdavi, MahboobeAndriukaitis, DariusMahdi, SamiraAndročec, DarkoMaheshwari, MuneeshAndrysek, JanMahler, RonaldAndrzej, MichalskiMahmood, AhmedAng, Chee SiangMahmoudabadi, HamidAng, Chee WeiMahmoudzadeh, AhmadrezaAng, Li-Minn Mahovic Poljaček, SanjaAng, Yee SinMaia, Marcio Espíndola FreireAngani, Chandra SekharMaier, Guenther A.Angelidis, PantelisMainini, Andrea GiovanniAngelini, MarcoMaio, LeandroAngotzi, Gian NicolaMair, DominikAngrisano, AntonioMaita, FrancescoAnicai, LianaMaiti, AnandaAnishchenko, LesyaMaiti, RishiAnitas, Eugen MirceaMaity, DebotyamAnitescu, CosminMaiuri, PaoloAnkhili, AmaleMaj, PiotrAnnamdas, Venu GMMaja, JoeAnnanouch, Fatima EzahraMajchrowicz, DariaAnnus, PaulMajd, NahidAnochi, JulianaMajda-Zdancewicz, EwelinaAnsaloni, GiovanniMajewski, LeszekAnsari, SardarMajor, ZoltanAnthony, CarlMaj-Zurawska, MagdalenaAntoce, Arina OanaMak, Pui-InAntolín, DiegoMakanae, KojiAntonakis, AristeidisMakantasis, KonstantinosAntonini, AlessioMakar, ArturAntonini, AndreaMaki, ToshihiroAntonio Costanzo, AntonioMaki, YasuyukiAntoniou, ConstantinosMakihara, KanjuroAntonopoulos, AngelosMakino, YoshioAntonopoulos, ChristosMäkitalo, NikoAntonopoulos, Christos P.Makoś, PatrycjaAnvari-Moghaddam, AmjadMakowski, ŁukaszAnwar, Syed MuhammadMakris, ChristosAnzano, Jesus M.Makris, SotirisAoni, Rifat AhmmedMaksimovic, FilipAparicio, NievesMalajner, MarkoAparicio, SofíaMalakar, NabinApolinário Jr., José AntonioMalara, PietroApostolos, XenakisMalde, KetiApplegate, Brian E.Malecha, KarolAprigliano, FedericaMałecki, KrzysztofAqueveque, PabloMalekan, MohammadAquino, AndreMalekian, RezaAquino, André Luiz LinsMalgosa-Sanahuja, JosemariaAquino-Lopez, Marco A.Malik, AdityaArafin, Md TanvirMalik, HassanArahal, Manuel R.Malinowska, AgnieszkaARAI, ShogoMalinowski, PawelArakawa, ToshiyaMalinowski, Paweł H.Aramesh, MortezaMalinverni, Eva SavinaArami, ArashMalitesta, CosiminoArana-Arexolaleiba, NestorMalka, DrorArandjelovic, OgnjenMalki Ebeid, Emad SamuelAranha, JoséMallardo, VincenzoAraszkiewicz, AndrzejMalli, RolandAraujo Dos Santos, José ViriatoMalone, BrendanAraujo, AlvaroMalvano, FrancescaAraujo, EvandoMalykin, Grigorii B.Araujo, FilipeMalyuskin, OleksandrAraujo, JeffersonMamdoh Elhalawany, BasemAraujo, William Reis DeMamrashev, AlexanderArazuri, SilviaMan, Ka LokArchambault, DominiqueManagò, StefanoArchMiller, Althea A.Manakhov, AntonArcia-Moret, AndrésManavalan, BalachandranArcudi, FrancescaMancini, AdrianoArdila-Rey, Jorge AlfredoManciola, PiergiorgioAréchiga, NikosMancisidor, AitziberArena, AntonellaMandal, BappadityaArevalo, ArpysMandal, KrishnaArgüelles, AmadeoMandal, SoumyajitArguelles-Cruz, Amadeo J.Mandal, SubhamoyArgüello, FranciscoMandigout, StéphaneAri, Ado Adamou AbbaMandolini, MarcoArias Garcia, JanierManera, Maria GraziaArias-Estrada, Miguel-OctavioManera, ValeriaAricò, PietroManeuski, DimaAristovich, Kirill Y.Manfred, KatherineAriyur, Kartik B.Manfreda, SalvatoreArmada, ManuelManfriani, LeonardoArmada, Manuel A.Mangina, EleniArmstrong, EveManhart, MichaelArmstrong, GlenMani, Ganesh KumarArmstrong, Roy A.Mani, VeerappanArnaud, GouelleManich, SalvadorArnó, JaumeManikas, AthanassiosArnold, Andrew J.Manis, GeorgeArnold, Francisco JoséManivannan, NadarajahArnoso, JoseManjakkal, LibuArora, AshishMańka, MichałArora, SiddharthMann, SteveArrebola, ManuelManogaran, GunasekaranArregui, FranciscoManojlović, Lazo M.Arribas, JavierManolakos, DimitriosArribas, Juan IgnacioManoli, KyriakiArriola, AitorMansoorzare, HakhamaneshArroyo Ohori, KenMansouri, MasoumehArroyo, PatriciaMantovani, MatteoArsenovic, PavleMantri, DnyaneshwarArtemjiew, PiotrManzanares-Lopez, PilarArtese, GiuseppeManzano, MarisaArtola, LaurentManzoor, AsifArvanitis, KonstantinosMao, BaoleiArwatz, GiladMao, HaiyangAryal, JagannathMao, HongjuArza Valdés, AdrianaMao, LeiAsadi, SomayehMao, QingzhouAsadnia, MohsenMao, XinhuaAsai, TakehikoMao, YaoAsanuma, IchioMao, ZhuAscorbe, JoaquinMaran, ViniciusAsghari, HosseinMaranesi, ElviraAshley, JonMarani, RobertoAshok, AshwinMarapareddy, RamakalavathiAshour, Ashraf A.Marasović, TeaAshraf Gandomi, YasserMarasso, Simone LuigiAshraf, ImranMarcato Júnior, JoséAshrafi, AmirmansoorMarcato, JoséAsif, RameezMarcelli, RomoloAsimellis, GeorgeMarcelot, OlivierAskraba, SretenMarchesini, IvanAsorey-Cacheda, RafaelMarchetti, DedaloAspandiar, MehroozMarchetti, MircoAspinall, MichaelMarchiori, AlanAsrar, Ghassem R.Marchuk, OleksandrAssaf, TareqMarco Gisbert, HectorAssante, DarioMarco, MauriAssi, SulafMarcolin, FedericaAssis, Karcius Day RosárioMarcon, PetrAssmann, MarcMarcos, MarcosAssunção, PedroMarcos-Pablos, SamuelAstarita, VittorioMarcotegui, BeatrizAstolfi, DavideMarechal, PierreAsua, EstibalizMarek, GancarzAsundi, SharanMarfievici, RamonaAsyhari, A. TaufiqMarghany, MagedAtamuradov, VepaMargheri, AndreaAta-Ul-Karim, Syed TahirMargheritis, EleonoraAthanasia, PrintzaMaria Simona, RaboacaAthanasiou, AlkinoosMaria, TimofeevaAthanassios, SkodrasMaria, Vila SantosAtkinson, GaryMariani, CamilloAtman, AllbensMariani, StefanoAttaran, AliMariescu-Istodor, RaduAttia, AhmedMarin, FredericAttolico, GiovanniMarin, PilarAtukeren, ErdalMarinelli, LucioAudu, Musa L.Marinelli, MarioAugust, IsaacMarinello, FrancescoAugustyniak, PiotrMarinesco, StéphaneAurangzeb, KhursheedMarin-Hernandez, AntonioAuzelle, ThomasMarini, SimoneAvanaki, Mohammad R. N.Marino, GiuseppeAvaria, GonzaloMarios, AnagnostopoulosAvdelidis, Nicolas PMarjanović, MiroslavAvery, JamesMarjańska, EwaAvila, Sergio LucianoMarkakis, Evangelos K.Avila-Paredes, Hugo J.Markelin, LauriAvilés, FrancisMarkevicius, VytautasAvina-Cervantes, Juan GabrielMarkfort, CoreyAvola, DaniloMarkiewicz, JakubAvolio, AlbertoMarkopoulos, AngelosAvram, CameliaMarkou, IouliaAvramescu, ViorelMarkov, DmitryAvramov-Zamurovic, SvetlanaMarkovic, Goran B.Avtar, RamMarkovic, MilanAw, KeanMarkowski, KonradAwad, Ali IsmailMarks, Haley L.Awad, MohamadMarotti De Sciarra, FrancescoAwin, FaroqMarozas, VaidotasAwolusi, IbukunMarpu, Sreekar BabuAwual, Md. RabiulMarques, CarlosAYADI, AbderrahmaneMarques, GonçaloAyala-Ramirez, VictorMarques, Manuel JorgeAyesta, IgorMarquez, Francisco MAyoub, NaeemMarrazza, GiovannaAyyash, MoussaMarrone, StefanoAzad, AbulMARS, KamelAzam, Saeed Eftekhar (Greece)Marsal, Lluis F.Azam, Saeed Eftekhar (USA)Marshall, Mark TAzar, MassoodMarston, Hannah R.Azaria, AmosMarszalek, ZbigniewAzinheira, JoséMarta, Ferreiro-GonzálezAzorín-López, JorgeMartalò, MarcoAzpilicueta, LeyreMartarelli, MilenaAzzouz, ImadeddineMartian, AlexandruBaala, OumayaMartin, AidaBaars, Woutijn J.Martin, FerranBabarada, FlorinMartín, FerranBabbar, RohitMartin, Francis LBabic, VesnaMartín, HonorioBacanin, NebojsaMartín, Jesús SánchezBacciocchi, MicheleMartín, Joan NavarroBacco, ManlioMartin, KeithBachler, MartinMartin, Richard K.Bachmann, MarcelMartin, VincentBačić, BorisMartín-Alcón, SantiagoBackarich, ShannonMartinčić - Ipšić, SandaBackes, André RicardoMartincic, EmileBacurau, RodrigoMartindale, Christine F.Badau, DanaMartinek, RadekBaddour, NatalieMartinelli, MassimoBadea Doni, MihaelaMartinet, JeanBadea, MihaelaMartinez Espinosa, Juan CarlosBader, SebastianMartínez Gila, Diego ManuelBadescu, GabrielMartinez Lacasa, DaniBadets, FranckMartìnez Monterrubio, Sergio MauricioBadia-Melis, RicardoMartínez Torres, JavierBae, Eui WonMartinez, AntoniBae, Hyung D.Martinez, DominiqueBae, HyungdaeMartínez, José-FernánBae, JihyunMartínez, Juan A.Bae, Tae-SukMartinez, ManuelBae, YoungchulMartinez, MarciasBaek, Kwang RyulMartinez, Maria SofiaBaek, Soo-WhangMartinez, Ramses V.Baeza, MireiaMartinez-Agirre, AlexBagherifard, SaraMartínez-Arias, RosaBagnaninchi, PierreMartínez-Barberá, HumbertoBahraminejad, BehzadMartinez-Carranza, JoséBai, GengMartínez-Carricondo, PatricioBai, LongMartínez-De-Dios, José RamiroBai, LuMartinez-de-Rioja, EduardoBai, OuMartínez-García, EdgarBai, ShaopingMartinez-Izquierdo, EstibalizBai, WeihuaMartínez-Lillo, JoséBai, XiangMartínez-López, J. IsraelBai, XianxuMartínez-Martínez, AlejandroBai, YongMartinez-Mora, Juan AntonioBaik, SungMartínez-Pellitero, SusanaBailey, DerekMartinez-Rios, AlejandroBailey, JosephMartínez-Ruiz, VirginiaBailey, SeanMartinez-Valdes, EduardoBaiocchi, ValerioMartínez-Villaseñor, LourdesBais, AbdulMartini, AlbertoBajer, JosefMartini, DougBajrami, XhevahirMartin-Lopez, SoniaBaker, BenMartino, José Mario DeBaker, BruceMartins Da Silva, MairaBaker, Kyri A.Martins, AlfredoBaker, TharMartins, Alfredo Manuel De OliveiraBakken, David E.Martins, Fernando RamosBakr, MustafaMartins, MarcosBaksi, AnanyaMartins, RodrigoBala, CameliaMartins, Verónica C.Balabas, MikhailMartins, Wallace AlvesBalachandran, SweeMartinsen, Ørjan G.Balado Frias, JesusMartin-Torres, JavierBalado Frías, JesúsMartowicz, AdamBalaguru, RajavelMartyshko, Petr S.Balali, VahidMaruda, Radosław W.Balan, OanaMaryska, MilosBalandin, SergeyMarzi, ClaudiaBalasubramanian, KannanMarzo, AsierBalasubramanian, VenkatramanMarzocca, CristoforoBalawejder, MonikaMarzullo, Vincenzo ManuelBalchanos, MichaelMasahiro, MotosukeBaldacchini, ChiaraMasamune, SadaoBaldauf, MichaelMasayuki, AnyojiBaldini, GianmarcoMasayuki, SohgawaBaldominos, AlejandroMascaro, LuciaBaldoncini, MaricaMascini, MacelloBalestra, GabriellaMašek, PavelBalitskii, AlexanderMasetti, GiuseppeBallato, JohnMashhadi, Mahdi BoloursazBallesta, MonicaMasiello, GuidoBallico, EdoardoMasiero, AndreaBallmann, ChristopherMasini, Barbara MaviBaloglu, UlasMasini, Barbara MavìBalsa-Barreiro, Jose (Switzerland)Masini, Jorge CesarBalsa-Barreiro, Jose (USA)Masini, NicolaBalss, UlrichMasip, DavidBaltazar, ArturoMaskeliunas, RytisBalzer, FrankMaskeliūnas, RytisBambling, MatthewMasquelier, TimothéeBamer, FranzMassa, DanieleBanan Sadeghian, RaminMassaglia, GiuliaBandera, JuanMassari, FrancescoBandodkar, Amay J.Massaro, AlessandroBandyopadhyay, SupriyoMassaroni, CarloBane, Susan L.Massera, EttoreBanerjee, AmitMast, T. DouglasBanerjee, PratikMastelaro, Valmor R.Banerjee, RumanMastinu, EnzoBanerjee, TanviMastmeyer, AndreBang, HyochoongMastrangeli, MassimoBani Younes, MaramMastroianni, CarloBankov, DmitryMasuda, YoichiBañón, LuisMatano, FabioBansmer, StephanMatatagui, DanielBao, JiatongMatejec, VlastimilBao, LijunMateo, DavidBao, TengfeiMateo, ReyesBao, Yi (Missouri University of Science and Technology)Mateo, TomásBao, Yi (Stevens Institute of Technology)Mateos, AlfonsoBao, Yi (University of Michigan-Ann Arbor)Mateos, CristianBao, YinyinMaterer, Nicholas F.Baraka, MoustafaMaterka, AndrzejBarakh Ali, Sogra FathimaMatetić, MajaBaran, NikolaMateus, PedroBaran, RemigiuszMathias, Mauro HugoBaranov, PavelMatikas, Theodore E.Baranova, EkaterinaMatko, VojkoBaranowska-Korczyc, AnnaMatos Mascarenhas, DalbertBaranyai, LászlóMatos, João NunoBarba, SalvatoreMatrella, GuidoBarbarin, YohanMatsuguchi, MasanobuBarbedo, JaymeMatsukura, HarukaBarber, RamónMatsumura Araujo, RicardoBarbin, DouglasMatsuura, YujiBarbon Jr, SylvioMatthias, BjörnBarbosa, Ana IsabelMatthies, Denys J.C.Barbosa, Celio FonsecaMatveev, SergeyBarbosa, Ramiro S.Matwijczuk, ArkadiuszBarbosa, Roberto SpinolaMatzner, ShariBarbosa, Talles Marcelo G De AMaulini, RichardBarceló-Ordinas, José M.Maundy, BrentBarclay, DavidMaurício, JoséBarczak, KamilMauriz, ElbaBard, DelphineMaybeck, VanessaBardall, GabrielleMayer, DirkBardas, Alexandru G.Mayer, JoceliBardera, AntonMaynard, PeterBardweel, HamzehMayor, JesúsBarek, JiriMayor, John Jairo VillarejoBarenji, Ali VatankhahMayorov, AlexanderBarettin, DanieleMažeika, DaliusBarfidokht, AbbasMazher, AbeerBarille, RegisMazuelas, SantiagoBarnes, Christopher FMazuera, Sandra CanoBaron, CoreyMazur, RadosławBaron, RonanMazurek, PawełBarone, Pier MatteoMazurek, PrzemysławBarrado, CristinaMazzeo, Pier LuigiBarralon, PierreMazzotti, DavideBarreca, SalvatoreMcCamley, JohnBarrera, EduardoMcClean, SallyBarresi, GiacintoMcClincy, Michael P.Barrientos, AntonioMcCool, ChrisBarrientos, CarolinaMcCoy, Mark D.Barrile, VincenzoMcDonald, AnthonyBarriot, Jean-PierreMcDonald, TimothyBarros, Michael TaynnanMcEwan, AlistairBarros, NelsonMcFarlane, NicoleBarski, MarekMcgibbon, ChrisBarsocchi, PaoloMcGibney, AlanBarthel, FrankMcGiffen, Tom GlennBarthelemy, JohanMcGinnis, RyanBartlett, Michael D.McGinnis, Ryan S.Bartolomeo, Antonio DiMcGonigle, AndrewBartolomeu, Paulo C.Mcgookin, EuanBarton, Michael (Israel)McGough, Robert J.Barton, Michael (Spain)McGrath, DanielBartonova, AlenaMcGrattan, Kevin B.Bartsch, HeikeMcGraw, Christina M.Bartyś, MichałMcGrory, Brian J.Bartzis, JohnMcGuire, MichaelBarua, ShaibalMcGuire, PatrickBarukčić, MarinkoMcheick, HamidBaryannis, GeorgeMcIntyre, Dustin L.Baryshnikova, EkaterinaMcKee, KristofferBarz, CristianMcKeever, SusanBarzilov, AlexanderMcKenna, Peter EdwardBaś, BogusławMcKerrow, AlexaBasarab, Miklhail AlekseevichMckinna, LachlanBasbas, SocratesMcLauchlan, LiffordBasford, Philip J. McLennan, KristaBasha, ElizabethMcLeod, EuanBashkatov, Alexey NikolaevichMcLeod, Robert D.Bašić, TomislavMcManus, KillianBasit, Munshi MahbubulMcNamara, ShamusBassani, CristianaMcneill, StephenBassett, ChristopherMcPeak, Kevin M.Bassler, MichaelMcPheron, Benjamin D.Bassoli, RiccardoMeagher, RobertBasteris, AngeloMeattini, RobertoBastidas, David M.Medeiros, HenryBastide, RémiMedhat, FadyBastos, Guilherme S.Medina Quero, JavierBastos, JulioMedina, DanielBastos, Teodiano FreireMedina, RubenBasu, AnirbanMedley, AnnBasu, DebrajMedola, FaustoBaszanowska, EmiliaMedrano, CarlosBatagelj, BostjanMedrano, NicolásBateman, MartinMeduri, AlessandroBatina, NikolaMedvidović-Kosanović, MartinaBatista, Arnaldo GuimarãesMeek, SanfordBatra, A. K.Meenakshi Sundaram, BalamuruganBatsakis, SotirisMegill, William M.Battistel, AlbertoMegret, PatriceBattistelli, GiorgioMeharouech, AmiraBattistini, SimoneMehdipour, ImanBatule, Bhagwan SahebraoMehmood, RashidBaturone, IluminadaMehrandezh, M.Baty, FlorentMei, HaiboBau Macià, JosepMei, HanfeiBaù, MarcoMei, LeiBauer, DietmarMei, LiangBaumann, BerndMeikle, WilliamBausells, JoanMeisel, ChristianBausys, RomualdasMejia-Beltran, EfrainBawaneh, KhaledMejias, LuisBax, CarmenMejía-Salazar, J. RicardoBayon, YvesMekhalfi, Mohamed LamineBazazian, DenaMekki, KaisBazydło, GrzegorzMelchor, JuanBazzi, Alessandro (Consiglio Nazionale delle Ricerche)Meldrum, A.Bazzi, Alessandro (University of Bologna)Meli, EnricoBeach, KevinMelkonian, Jean-MichelBeach, TomMellado, NicolasBeard, James D.Mellin, PatriciaBearzotti, AndreaMello, LuizBęben, AndrzejMellone, SabatoBec, KrzysztofMelnykowycz, MarkBec, Krzysztof B.Melo, Ana Claudia Do AmaralBecek, KazimierzMelvin, AdamBechelany, MikhaelMemisoglu, GorkemBechhoefer, EricMemmi, GerardBecker, Leandro BussMemmolo, VittorioBecker, RichardMemon, ImranBecker, RolfMena, EduardoBecucci, MaurizioMenachery, AnoopBednar, SlavomirMenard, DavidBedon, ChiaraMenchaca-Méndez, RolandoBeer, MartinMendecki, LukaszBeghi, RobertoMendes, JérômeBegušić, DinkoMendes, RenatoBeheshti, ImanMendes, Virgilio BritoBehjat, VahidMéndez, AlexisBeilina, LarisaMendez, Martin OswaldoBeirnaert, CharlieMendez, SimonBekas, Dimitrios G.Mendicute, MikelBeko, MarkoMendikute, AlbertoBel, AlbertMendonça, LaércioBelagiannis, VasileiosMendoza, DanielBelavič, DarkoMenegaldo, Luciano L.Belayat, HossainMeneguette, RodolfoBelBruno, JosephMenendez, J. M.Belenguer, TomásMeneses, FilipeBelfiore, Nicola PioMenezes, PauloBelgiu, MarianaMeng, FanbenBeliatis, Michail J.Meng, FanliBell, KevinMeng, FanyuBell, TylerMeng, GangBellekens, XavierMeng, GuowenBelli, LauraMeng, JihuaBellido Outeiriño, Francisco JoséMeng, MengBellizzi, Gennaro G.Meng, RanBellman, C. J.Meng, WeiBellone, MauroMeng, WeinaBellos, EvangelosMeng, WeizhiBellucci, PatriziaMeng, XiangyuBeltrame, GiovanniMeng, YuanBen Haj Frej, MohamedMeng, ZhaozongBen Ishai, PaulMeng, ZiboBen, YueyangMengarelli, AlessandroBenalia, SourayaMenna, FabioBenavent-Climent, AmadeoMensi, MounirBenavente, CésarMentiplay, BenjaminBenbouzid, MohamedMenzel, Siegfried BernhardBenca, EmirMeola, CarosenaBender, PhilippMerad, DjamalBenedetti, IvanoMerazzo, Karla J.Benedetto, AlexandreMercado, Jesús M.Benedetto, Jose I.Mercante, LuizaBenedicenti, LuigiMercier, DavidBeneš, PetrMercorelli, PaoloBenetti, MassimilianoMeredith, NathanBeni, ValerioMerenda, MassimoBenini, AlessandroMerlino, GiovanniBenitez-Perez, HéctorMerlo, SabinaBennasar, MohamedMerola, Simona SilviaBennett, Mia M.Meron, MosheBennett, MichaelMerriaux, PierreBen-Othman, JalelMerrick, TrinaBenseman, Timothy M.Mesas-Carrascosa, Francisco JavierBenson, LaurenMeschino, Gustavo JavierBenz, HeatherMeseguer, RocBenzerrouk, HamzaMeshgi, KouroshBeranek, LadislavMesodiakaki, AgapiBerardi, Valentino PaoloMessier, Kyle P.Berardo, AliceMessina, MarcoBerbakov, LazarMestdagh, HélèneBerbecaru, DianaMeštrović, AnaBerder, OlivierMethapettyparambu Purushothama, JayakrishnanBereś-Pawlik, ElżbietaMeulenberg, ElineBergamasco, FilippoMeyer, SarahBergamasco, LucianaMeynants, GuyBergamini, ElenaMeyyappan, MeyyaBerger, CyrilleMeza-Ruiz, Iván VladimirBerger, KatjaMezei, JózsefBergillos, Rafael J.Mezzanotte, PaoloBergin, MichaelMhlongo, NH3 SensoHlengiweBerglund, RobinMiani, Rodrigo SanchesBergmann, OlafMiao, CunxiaoBeritelli, FrancescoMiao, FenBerkovic, GarryMiao, PengBerkvens, RafaelMiao, QiangBerman, MichaelMiccadei, EnricoBermudez-Cameo, JesusMichael Ho, Siu ChunBernacki, BruceMichael, AronBernard, BrianMichael, BrendanBernard, PaulineMichaelides, Michalis P.Bernardi, GiulioMichalak, SławomirBernardini, RiccardoMichalas, AntonisBernardini, SandrineMichalis, PanagiotisBernardino, JorgeMichanowicz, Drew R.Bernardo, LuisMichel, Carlos R.Bernards, MatthewMicheli, LauraBernaś, MarcinMichelini, ElisaBerni, RossellaMichieletto, StefanoBerrett, ThomasMichihata, MasakiBerretti, StefanoMichna, MichałBertemes-Filho, PedroMicic, MiodragBertini, LucianoMiclaus, SimonaBertini, MarcoMico Amigo, EncarnaBertling, KarlMidtiby, Henrik SkovBertocci, FrancescoMiękus, NataliaBertolo, EmíliaMiele, GianfrancoBertoncello, PaoloMieloszyk, MagdalenaBertrand, Olivier J.NMiettinen, JariBertuccio, GiuseppeMietzner, JanBerveglieri, AdilsonMigallon, HectorBerzi, LorenzoMigdalski, JanBesada, JuanMigliorati, PierangeloBesagni, GiorgioMiguel, Angel ClimentBessa, Iury ValenteMihailescu, Marius IulianBessone, VeronicaMihajlović, VojkanBessonov, Vladimir O.Mihara, YujiBestak, RobertMihaylova, LyudmilaBétaille, DavidMihelj, MatjažBetelu, StéphanieMikhaylov, KonstantinBevly, DavidMikhelson, KonstantinBeyerer, JurgenMikita, TomášBeyerer, JürgenMikkelsen, KaareBeyerle, GeorgMiklic, DamjanBezerra, Eduardo AugustoMiklos, ZoltanBhadra, SharmisthaMikó, BalázsBhagat, YusufMikolajczyk, TadeuszBhandari, Manohar PrasadMikrut, SlawomirBhandari, SantoshMikš, AntonínBhargava, KritiMikulka, JanBharti, PratoolMikulski, JerzyBhatta, MadhavMilad Ramezani, MiladBhattacharya, DevanjanMilanic, MatijaBhattarai, Jay K.Milanova, MariofannaBhounsule, Pranav A. (University of Illinois)Milde, MoritzBhounsule, Pranav A. (University of Texas at San Antonio)Milecki, AndrzejBhuiyan, Md YeasinMilej, DanielBhuiyan, Mohammad Zahidul H.Milella, AnnalisaBhuiyan, YeasinMilenko, KarolinaBhutta, RaheelMiles, DavidBia, PietroMiles, VictoriaBiagetti, GiorgioMileti, IlariaBialas, KatarzynaMilham, PaulBianchi, ValentinaMilicevic, MarioBianchini, MonicaMilik, AdamBianchini, SilviaMiljković, NadicaBianconi, FrancescoMillán, BorjaBiasiucci, AndreaMillard, KoreenBiaz, SaâdMiller, AlexanderBicen, OzanMiller, Benjamin L.Bichurin, MirzaMiller, BorisBie, RongfangMiller, JayBielecka, ElżbietaMiller, ThomasBień, AndrzejMills, DavidBienias, JaroslawMiluski, PiotrBieniek, TomaszMin, Jun-HongBifulco, PaoloMin, MartBigdeli, SiavashMin, RuiBiglia, AlessandroMin, Se DongBikakis, AntonisMinaee, ShervinBilas, VedranMinamiki, TsukuruBilasco, Marius IoanMinardo, AldoBilbao, JuliaMinas, GraçaBilén, Sven G.Minati, LudovicoBilík, PetrMinerva, RobertoBilleci, LuciaMinet, PascaleBillerbeck, SonjaMING, ZuhengBimbo, JoaoMingesz, RobertBinietoglou, IoannisMinglei, TongBiondi, FilippoMinguillon, JesusBirgand, FrancoisMiniaci, MarcoBirk, AndreasMinin, IgorBirlan, MirelMinjarez-Sosa, CarlosBiro, IstvanMinussi, Carlos RobertoBisaglia, CarloMiralles-Ricós, RamónBiscaia, Hugo C.Miramini, SaeedBischof, Hans-PeterMirauda, DomenicaBishop, GregMirbozorgi, AbdollahBisio, IgorMirea, TeonaBisogni, Maria GiuseppinaMirica, KatherineBiswas, DwaipayanMiridonov, SergueiBitar, Ahmad W.Mirsayar, MirMiladBithas, PetrosMirsky, Vladimir M.Bithas, Petros S.Mirzaei, AliBittencourt, ChristianoMirzavand, RashidBittencourt, Luiz FernandoMischler, StefanoBjerrum, Esben JannikMiscourides, NicholasBjorklund, RobertMishin, Alexander S.Bjorklund, Robert B.Mishne, DavidBjörling, Elin A.Mishra, AmitabhBjorninen, ToniMishra, DeependraBjörninen, ToniMishra, Geetesh KumarBłachowicz, TomaszMishra, Geetesh K.Blachowski, BartlomiejMishra, Kumar VijayBlack, Bryan JamesMishra, Rupesh K.Blaimer, MartinMishra, Yogendra KumarBlanch, JuanMišić, JelenaBlanchon, MarcMiska, MarcBlanco Rojas, Maria DoloresMiskowicz, MarekBlanco-López, María CarmenMiskulin, IvanBlanes, CarlosMissell, FrankBlanes, JorgeMissell, Frank P.Blankenbach, JörgMissinne, JeroenBlanloeuil, PhilippeMisu, ShogoBłaszczak-Bąk, WioletaMitani, AtsushiBlau, WernerMitchell, JohnBlazejcyk-Majka, LucynaMitchell, KennethBleichner, MartinMitishita, EdsonBlekas, KonstantinosMitka, BartoszBlesa Izquierdo, JoaquimMitra, SrinjoyBlin, StéphaneMitrovic, VesnaBlume, HolgerMitrovic-Minic, SnezanaBlumenschein, Laura H.Mitryukovskiy, SergeyBlumrosen, GaddiMitsubayashi, KohjiBoada, Beatriz L.Mitsukura, YasueBoada, Maria Jesus LopezMittal, SudipBoaventura, ThiagoMiura, HiroyukiBoavida, FernandoMiyamoto, KoichiroBobda, ChristopheMiyamoto, TomoyukiBobek, SzymonMiyazaki, HiroyukiBobinger, MarcoMizoshiri, MizueBobulski, JanuszMizuyama, TakahisaBochenek, DariuszMlejnek, PavelBochicchio, MarioMłyńczak, MarcelBochtis, DionysisMo, YuBocicor, IulianaMoazeni, SajjadBocicor, Maria LulianaMobasheri, AminBoden, StephanMobilio, MarcoBodini, MatteoMocanu, Irina GeorgianaBodkhe, SampadaMoczko, PrzemyslawBogaerts, JanModica, GiuseppeBogdan, MihaiModoni, GianfrancoBogdan, PaulMøgelmose, AndreasBogdan, RazvanMoglie, FrancoBogdanowicz, KrzysztofMogo, SandraBogdanski, PawelMoh, SangmanBohak, CirilMohammad, MahdaviBohm, EmanueleMohammad, ShaqfehBohorquez, JorgeMohammed, ZakriyaBoiko, DmitriMohapatra, JeotikantaBojic, IvaMohd-Yasin, FaisalBolea, JuanMohorcic, MihaelBolic, MiodragMohri, KaneoBollella, PaoloMöhring, Hans-ChristianBologa, Ana-RamonaMois, GeorgeBolomey, Jean-CharlesMoiseev, SergeyBolotin, Yu V.Moisescu, Mihnea AlexandruBolshov, MikhailMokhlespour Esfahani, Mohammad ImanBolten, AndreasMokhtarzadeh, HosseinBombek, GorazdMolardi, CarloBonaci, TamaraMolin, Jose PauloBonafoni, StefaniaMolina López, JoseBonastre Pina, Alberto MiguelMolina Martínez, José MiguelBonastre, AlbertoMolina, José-LuisBonci, AndreaMolina-Solana, MiguelBond, JasonMoll, JochenBONDOC, IonelMolleda, JulioBondorf, SteffenMolnar, ArthurBone, GaryMon, Yi-JenBonenberg, LukaszMonasse, PascalBongioanni, CarloMondal, Tapas KBoni Vicari, MatheusMonekosso, DorothyBonì, RobertaMongiardo, MauroBoniface, KarenMongus, DomenBonizzoni, MarcoMonica, RiccardoBonomo, Anthony L.Monica, StefaniaBonomolo, MarinaMoñino, AntonioBonyár, AttilaMonkawa, AkiraBoori, Mukesh SinghMonnai, YasuakiBora, Ganesh C.Monno, YusukeBoragno, CorradoMonroy, JavierBorboni, AlbertoMonsivais-Huertero, AlejandroBorges, JoelMontalvo, CarlosBorghi, AlessandraMonte Lima, João Paulo Silva DoBorghi, GuidoMontecchi, LeonardoBorghini, GianlucaMonteiro Gonçalves, José ManuelBorhani, AlirezaMonteiro, FranciscoBoric-Lubecke, OlgaMonteiro, JoãoBorisov Todorov, IvanMontejo Sánchez, SamuelBorja, Alejandro LucasMontenegro, DavisBorkowski, PiotrMonteriù, AndreaBörner, AnkoMontero Quispe, Kevin G.Borrego, CarlosMontero, Rocío SánchezBorsi, HosseinMonterroso-Checa, AntonioBorza, Paul NicolaeMontes-García, VerónicaBos, MachielMontgomery, JackBosch, StephanMontinaro, NicolaBosché, FrédéricMontisci, AugustoBoschetti, LuigiMontoliu, RaúlBoschi, LapoMontrésor, SilvioBoškoski, PavleMontrucchio, BartolomeoBossavit, BenoitMontuori, AntonioBostater, Charles R.Montusiewicz, JerzyBotta, MarcoMonzo, CarlosBoubchir, LarbiMonzón-Hernández, DavidBoudaden, JamilaMoo Lee, ByungBouffanais, RolandMoo, PeterBougas, LykourgosMoon, HyeonjoonBouguila, NizarMoon, Kee S.Bouhamed, AydaMoon, WonkyuBouk, Safdar H.Moore, NathanBoukhechba, MehdiMoore, PhilipBoukouvalas, ZoisMoorman, ValerieBoulogeorgos, Alexandros-Apostolos A.Moosavi-nasab, MarziehBourdoux, AndreMoosazadeh, MahdiBourne, RogerMoosmuller, HansBourqui, JeremieMora, HiginioBousquet, Jean-FrancoisMorabito, Andrea FrancescoBoutaayamou, MohamedMorabito, Francesco CarloBoutami, SalimMoraes, Ricardo Alexandre Reinaldo DeBoutayeb, HalimMora-Gimeno, Francisco JoseBouteiller, Jean-Marie C.Morago, BrittanyBoutejdar, AhmedMoragrega, AnaBoutet, AntoineMorais, Jorge E.Boutteau, RémiMorais, RaulBouvet, Pierre-jeanMorales Maqueda, Miguel ÁngelBouwmans, ThierryMorales Narváez, EdenBouziane, BrikMorales, NéstorBovenga, FabioMorales-Luna, GuillermoBowen, ChrisMorales-Ponce, OscarBowland, Christopher C.Moran, JamesBowles, Jeffrey H.Morán, LaraBoya, CarlosMorandi, FedericaBoybay, Muhammed SaidMorar, AncaBoyd, AnthonyMorassi, AntoninoBoyd, DoreenMorasso, CarloBoyko, OlgaMorávková, ZuzanaBozomitu, Radu GabrielMoreau, JulienBraasch, KatrinMoreira Gonçalves, LuísBraasch, MichaelMoreira, AdrianoBračun, DragoMoreira, Adriano Jorge CardosoBrad, RemusMoreira, Felismina T.C.Bradbury, MatthewMoreira, Rui SilvaBradley, JonathanMorell, AntoniBradley, Patrick ErikMoreno, JaimeBradley, Robert SMoreno, Miguel ÁngelBraga, Guilherme S.Moreno-Hernández, DavidBragança, LuísMoret-Tatay, CarmenBragazzi, Nicola LuigiMoretti, GiacomoBragheri, FrancescaMoretti, LuigiBrajard, JulienMorettin, PedroBranch, PhilipMorettini, MicaelaBranco, FredericoMorgera, Alessandro FraleoniBrandão, AlexandreMori, SaverioBrandão, Lincoln CardosoMoriello, Rosario Schiano LoBrandl, MartinMorillo, Daniel SánchezBrandusoiu, IonutMorimoto, YoshiharuBrás, SusanaMoriñigo, DanielBratashov, Daniil N.Moríñigo-Sotelo, DanielBratasz, ŁukaszMorioka, KazuyukiBratcu, Antoneta IulianaMorishita, YuBraud, TristanMoritz, Guilherme LuizBrauer, HartmutMoriuchi-Kawakami, TakayoBraun, SebastianMoriya, HirokazuBraun, UlrikeMornieux, GuillaumeBravo, IgnacioMoro, ArturBray, JoeyMoro, ChristianBrcic, DavidMoron, CarlosBreaz, Radu EugenMoroni, DavideBrehar, RalucaMoroni, MonicaBrekke, EdmundMorosi, SimoneBrembeck, JonathanMorotti, ElenaBremer, KortMorozov, OlegBrena, Ramon F.Morozov, Oleg G.Brennan Jr., Thomas M.Morozs, NilsBrescia, MassimoMorshed, Bashir I.Brestic, MarianMorsy, SalemBrethé, Jean FrançoisMortari, DanieleBreuil, PhilippeMortazavi, JackBreuß, MichaelMosa, IslamBrezočnik, LucijaMosallam, AhmedBridgelall, RajMosavi, AmirBright, Frank V.Moschitta, AntonioBrisaboa, Nieves R.Moschopoulou, GeorgiaBrisolara, Lisane Brisolara DeMoseke, ClausBrisset, HuguesMoshou, AlexandraBrito, AlissonMoshou, DimitriosBrocherie, FranckMosko, MartinBrock, StefanMossi, KarlaBrockherde, WernerMössner, MartinBrodsky, LukasMostafaei, HabibBrognara, LorenzoMostarac, PetarBromuri, StefanoMota, BernardoBronson, KevinMota, Rafael Perazzo BarbosaBrook, AnnaMota, Virgínia FernandesBröring, ArneMotamedi, AliBrosed, Francisco JavierMotta, GianmarioBroumandan, AliMouapi, AlexBrown, Lyndon J.Moubayed, AbdallahBrown, StephenMouchet, SébastienBrown, Tim W. C.Moudry, VítezslavBrown, TimothyMoukadem, AliBrščić, DraženMoulas, EvangelosBruce, Neil C.Moulick, AmitavaBruder, StephenMoullec, Yannick LeBruggemann, Troy SterlingMoumen, Ahmed ElBruna, ArcangeloMourad-Chehade, FarahBrundage, Cord M.Mourtzios, ChristosBrunelli, DavideMourtzis, DimitrisBruneo, DarioMousas, ChristosBrunet, Anton GuimeràMousavi Kahaki, Seyed MostafaBrunete, AlbertoMousavi Lajimi, S. AmirBrunn, AnsgarMousavi, ZekraBrunner, GinoMoussa, MohamedBruno, FabioMoutinho, LuisBrunone, BrunoMoyà Alcover, GabrielBrus, JanMoya-Albor, ErnestoBruschi, PaoloMoyne, JamesBrydegaard, MikkelMozerov, Mikhail G.Brzostowski, KrzysztofMrissa, MichaelBu, XiongzhuMróz, MarekBubola, MarijanMrugalska, BeataBucchiarone, AntonioMrukiewicz, MateuszBuchecker, MichaelMu, XiaojingBuciakowski, MariuszMuanenda, YonasBuckley, ChristopherMucchi, LorenzoBuckley, JohnMück, MichaelBucur, BogdanMudie, KurtBudge, Scott E.Mudigonda, SandeepBudidha, KarthikMuehlsteff, JensBudinski, VedranMuhammad Arslan, UsmanBudwig, RalphMuhammad, KhanBudzan, SebastianMuhammad, Mannan SaeedBueno, JulianMuheidat, FadiBuescher, WolfgangMühlberg, Jan TobiasBuffi, AliceMuhle, PaulBugaric, MarinMujica, GabrielBuhot, ArnaudMukherjee, MithunBui, Dieu Tien (Ton Duc Thang University)Mukin, RomanBui, Dieu Tien (University of South-Eastern Norway)Mula, GuidoBui, NgocMulas, MarcoBuis, Ernst JanMulcahy, BenBujnowski, AdamMulla, YusufBukin, OlegMüller, Fabian De PonteBulić, PatricioMuller, GerhardBulteel, OlivierMüller, GerhardBulushev, DmitriMüller, JoachimBuonocore, FrancescoMüller, Julian MariusBurattini, LauraMuma, MichaelBurbey, ThomasMummolo, CarlottaBurchett, Bradley T.Mumolo, EnzoBurdziakowski, PawełMumtaz, ShahidBurgdorf, MartinMunafo, AndreaBurgués, JavierMunaretto, AneliseBuriro, AttaullahMunir, Kashif M.Burkholder, Robert J.Muñoz Organero, MarioBurlacu, AdrianMuñoz, AdolfoBurmistrova, Natalia A.Munoz-Aguirre, SeverinoBurrascano, PietroMunoz-Organero, MarioBurri, SamuelMunroe, Jeffrey S.Burstein, FradaMunschy, MarcBurtscher, HeinzMunster, PetrBury, WojciechMunteanu, Florentina-DanielaBuscaglia, MarcoMunz, MichaelBusch, CostasMuraccini, MarcoBush, NathanMurawski, LechBussmann, HansMurayama, RiichiBusson, AnthonyMuri, Harald IanBustamante, H.A.Muriel, Jorge BarriosBustin, AurelienMurillo-Escobar, Miguel AngelButka, BrianMurillo-Fuentes, Juan JoséButler, HansMurphy, James M.Butnariu, SilviuMurphy, MichaelButnaru, Gina IonelaMurray-Bruce, JohnButta, MattiaMurrieta-Mendoza, AlejandroButtafuoco, GabrieleMurtagh, FionnButtazzoni, GiuliaMurvay, Pal-ȘtefanBüttgen, BernhardMusacchio, MassimoButun, IsmailMusci, MirtoBuxi, DilpreetMusić, JosipBuzna, LubosMuskett, Reginald R.Buzzi, MarinaMusleh, BasamBykhovsky, DimaMust, IndrekByrne, HughMustafine, JamilaByrski, AleksanderMuszyński, ZbigniewByun, JunghwanMuthanna, AmmarByun, Yong-HoonMutlu, MeteC Jones, ErickMutschler, ChristopherC. Balan, MugurMuukkonen, PetteriC. Neves, JoãoMyasoedova, Tatiana N.Caballero-Mora, Maria AngelesMycielski, A.Cabal-Yepez, EduardoMyers, MikeCabboi, AlessandroMyers, SaraCabestany, JoanMyllymaa, SamiCabral, JaderMylonas, GeorgiosCabral, JorgeMystkowski, ArkadiuszCabrera, Wellington M.Na, In SeopCabri, GiacomoNa, Wongi SCaccavale, MauroNa, ZhenyuCaciolli, AntonioNabajeet, BarmanCadatal-Raduban, MarilouNabil, MahmoudCadore, Alisson RNabki, FredericCaesarendra, Wahyu (Australia)Nabok, AlexeiCaesarendra, Wahyu (Singapore)Nadeau, PhillipCaffarena, GabrielNadeem, MuhammadCai, BaopingNadig, SachinCai, ChenzhiNadolny, KrzysztofCai, FeiNadolny, ZbigniewCai, GuofaNafkha, AmorCai, HaipengNag, AnindyaCai, HaiwenNag, AvishekCai, JinguangNagahage, IsuraCai, JunNagahara, LarryCai, QingNagai, KoheiCai, WeiNagamine, KuniakiCai, WenyuNaganawa, JunichiCai, WuNagano, HanatsuCai, YimaoNagaraju, ManoharCai, YiyuNagel, James R.Caiani, Enrico GNaghar, AzzeddinCailean, Alin-MihaiNaghshvarianJahromi, MahdiCain, StephenNagi, ŁukaszCaizzone, StefanoNagles, EdgarCakmak, OnurNah, WansooCalabretta, Maria MaddalenaNahab, FattaCalado, João M. F.Naik, GaneshCalafate, Carlos TavaresNaik, Ganesh RCalbimonte, Jean-PaulNaik, Ganesh R.Calbó, JosepNajibi, NasserCaldas, Paulo (Instituto Politécnico de Viana do Castelo)Nakagawa, ShigekiCaldeira, João M. L. P.Nakamiya, ToshiyukiCaldeirinha, RafaelNakamoto, TakamichiCaldelli, RobertoNakanishi, YoshitakaCăleanu, Cătălin - DanielNakarmi, UkashCalegari, RobertaNakashima, HiroshiCalì, MicheleNakashima, ShotaCaliano, GiosueNakayama, KohCaliendo, CinziaNakayama, TomokiCaligiuri, Maria EugeniaNakayama, YuCaligiuri, Michael P.Nalepa, GrzegorzCalinescu, IoanNalepa, JakubCalisti, MarcelloNalewajko, Joanna SekulskaCalleja, Javier FernándezNallal, MuthuchamyCalleja-Arriaga, WilfridoNam, JeonghunCalvini, RosalbaNam, Yong UnCalvino, CélineNam, YunyoungCalvo, BelenNamazi, HamidrezaCalvo, HiramNamgaladze, AleksandrCalvo, IsidroNamiesnik, JacekCamacho, JorgeNamiot, DmitryCámara-Chávez, GuillermoNamuduri, KameshCamarena-Martinez, DavidNan, ZhuotongCamargo, Sandro Da SilvaNangia, VinayCamas Anzueto, Jorge LuisNanni, LorisCambiaso, EnricoNanni, MarcosCambou, BertrandNanver, Lis K.Cambria, ErikNapa, Sae-BaeCamerlingo, CarloNapieralski, PiotrCameron, NoelNapiórkowska-Krzebietke, AgnieszkaCamerota, FilippoNapoli, ChristianCamilli, LucaNappi, CiroCaminero Torija, Miguel ÁngelNar, FatihCamino, CarlosNaranjo, Jose EugenioCammarata, AlessandroNaranjo-Hernández, DavidCammarota, RosarioNarayanan, Ram MCampanella, Carlo EdoardoNarayanan, Ram M.Campbell, Joe C.Nardini, GiovanniCampbell, KathleenNardone, RobertoCampbell, KrisNarducci, FabioCampeau-Lecours, AlexandreNarman, Husnu SanerCampo Vázquez, CelesteNarracott, Andrew J.Campo, CelesteNasahara, Kenlo NishidaCampolo, ClaudiaNascimento Jr., Cairo L.Campos Souza, Paulo VitorNascimento, Daniel Chagas DoCampos, RicardNascimento, MicaelCampos, RuiNascimento, TiagoCampuzano, SusanaNaseer, Prof. NomanCamurri, MarcoNasello, CarmeloCancila, DanielaNaser, M. Z.Candefjord, StefanNasiri, NoushinCanedo, Edna DiasNasri, NadiaCanessa, AndreaNassar, SamehCanet Ferrer, JosepNassiopoulou, AndroulaCanetta, ElisabettaNassour, JohnCannata, GiorgioNastasi, BenedettoCaño-García, ManuelNastro, AlessandroCánovas, JoseNatale, EmanuelaCantatore, ValentinaNatarajan, AnnamalaiCantrak, DjordjeNath, ChandraCantzos, DemetriosNatkaniec, MarekCao, BiaoNatsuaki, RyoCao, ChangNattkemper, Tim W.Cao, ChuanhaiNauber, RichardCao, HaishiNaumova, ValeriyaCao, Hoang-LongNaus, KrzysztofCao, HuiNava, OmarCao, HuiliangNavaei, MiladCao, JianNavarro, ElenaCao, JianyunNavarro, GabrielCao, PengNavarro, Juan MiguelCao, RuiNavarro-Camba, Enrique A.Cao, WeiNavarro-Serment, Luis ErnestoCao, WenwuNavickas, RomualdasCao, YangNavikas, DangirutisCao, YanpengNaya Villaverde, Miguel ÁngelCao, YingchunNazarahari, MiladCao, YueNdehedehe, ChristopherCao, ZhangNDIAYE, Amadou LatyrCao, ZhiqiangNdieyira, JosephCao, ZhixingNeagu, GabrielCao, ZongjieNebel, Jean-christopheCaon, MaurizioNečas, DavidCao-Paz, Ana MaríaNedergaard, Niels J.Cap, SébastienNedoma, JanCapdeboscq, YvesNędzarek, ArkadiuszCapdevila, Daiana ANee, Jan BCapel, Manuel I.Nef, TobiasCapella, Juan VicenteNegru, MihaiCapineri, LorenzoNeilson, Peter D.Capolongo, DomenicoNele, LuigiCaponero, Michele ArturoNelson, Alexander H.Caporali, StefanoNelson, BrianCaporaso, NicolaNelson, JakeCappelletti, ChantalNelson, Jill K.Cappon, GiacomoNepomuceno, Erivelton GeraldoCappuzzello, FrancescoNeri, GiovanniCapra, AlessandroNeri, PaoloCapraro, FlavioNesterova, Irina V.Capriotti, MargheritaNestle, NikolausCapuano, RosamariaNestorović, TamaraCarabias-Orti, Julio J.Netto, Roberto S.Carballal, AdriánNeubert, JeremiahCarbon, Claus-ChristianNeuenschwander, JuergCarbonari, SandroNeumann, IngoCarbonaro, AntonellaNeumann, Patrick P.Carbonaro, NicolaNeves, Leandro A.Carbone, EmileNewaz, S.H. ShahCarbone, PaoloNewe, ThomasCárdenas, CésarNewman, Kimberly E.Cardenas-Juarez, MarcoNežerka, VáclavCardona-Morales, OscarNg, Alex Ching TaiCardone, DanielaNg, Derrick Wing KwanCardoso, Filipe ArroyoNg, JosephCardoso, JaimeNg, MarkCardoso, Jaime S.Ngo, Ha DuongCardoso, Rafael C.Ngo, Ha-DuongCarey, BenjaminNgo, Trung ThanhCarey, LawrenceNgo, Vuong M.Cariou, ClaudeNgomo, SuzyCarleer, RobertNguyen Gia, TuanCarlos, VâniaNguyen Tien, DatCarmen Pérez, MaríaNguyen, BinhCarminati, MarcoNguyen, Binh P.Carneiro, GlaucoNguyen, Binh VanCaroppo, AndreaNguyen, Duc PhucCarotenuto, FedericoNguyen, GiangCaroti, GabriellaNguyen, HoangCarpenter, DavidNguyen, HungCarra, DamianoNguyen, KienCarrasco, AlejandroNguyen, LinhCarrasco, MiguelNguyen, Minh TuanCarrascosa, FranciscoNguyen, Ngoc A.Carratù, MarcoNguyen, Ngoc TuCarreno-Luengo, HugoNguyen, NhaCarrera, José LuisNguyen, PhucCarretero, JesusNguyen, Quang NgocCarrillo Calleja, Juan ManuelNguyen, Tam Q.Carrion, DanielaNguyen, Thanh D.Carro Fernandez, GermánNguyen, Thien D.Carroll, MarkNguyen, Thien-MinhCarruth, DanielNguyen, ToanCartagena-Rivera, AlexanderNguyen, TrangCaruso, DavidNguyen, TrieuCarutasu, GeorgeNguyen, Trong-NguyenCarvajal Rodriguez, Migue AngelNguyen, TrungCarvajal-Ramírez, FernandoNguyen, TuCarvalho, DiegoNguyen, Tuan T.Carvalho, LídiaNguyen, Van DungCarvalho, PauloNguyen, Viet DungCasaca, WallaceNguyen-Thanh, NhonCasaccia, SaraNi, JunCasado-Gavalda, Maria P.Ni, ZhijiangCasado-Vara, RobertoNiazi, Imran KhanCasals Terre, JasminaNichol, CarolineCasals, AlíciaNico, GiovanniCasanella, RamonNicodemo, GianfrancoCasanova, RaimonNicol, John C.Casari, PaoloNicolae, MaximilianCasas Rius, Joan RamonNicolau, Maria JoãoCasas, OscarNicolay, PascalCasavola, AlessandroNicoletti, SergioCascini, Giuseppe LucioNicolò, AndreaCasiraghi, ElenaNicoloiu (Stefanescu), AlexandraCaspary, ReinhardNie, DongHuCasquel del Campo, RafaelNie, LanshunCass, TonyNie, LimingCassano, FabioNie, MengyanCassidy, John F.Nie, ZedongCasson, AlexanderNied, HermanCastada, HardyNiederleithinger, ErnstCastagno, JeremyNiekiel, FlorianCastañeda, Miguel A. PadillaNielsen, Jimmy JessenCastanheira, DanielNielsen, JohnCastaño, FernandoNiemczewska-Wojcik, MagdalenaCasteleiro-Roca, Jose LuisNieto, MarcosCastellano Paulis, JulenNifli, Artemissia-PhoebeCastellano, AnnaNiforatos, EvangelosCastellano, Javier GarcíaNiggemann, OliverCastellanos Garzón, José A.Niguès, AntoineCastellanos-Ramos, JuliánNiitsu, KiichiCastelli-Dezza, FrancescoNikitin, AlexeyCastellini, PaoloNikitin, PetrCastelló, Ramon CostaNikodem, JanCastiglione, ArcangeloNikolaev, DmitryCastillejo Parrilla, PedroNikolaev, ViacheslavCastillejo-Gonzalez, Isabel LuisaNikolaidis, AthanasiosCastillo Garcia, PedroNikolakopoulos, KonstantinosCastillo, EncarnaciónNikolaos Pagonis, DimitriosCastillo-Soria, Francisco RubénNikolaou, SymeonCastro Do Amaral, GustavoNikoleli, Georgia-ParaskeviCastro, Bruno A.Nikolka, MarkCasu, MarioNikolova, NataliaCasula, GiuseppeNikonorov, ArtemCataldi, PietroNiku, SaeedCataldo, AndreaNikulin, AlexCATALDO, RosellaNilfouroushan, FaramarzCatalfamo, PaolaNilghaz, AzadehCatalina, FernandoNilsson, TobiasCatarino, SusanaNimma, Kutaiba SabahCatena, RobertNimmagadda, Shastri L.Catherwood, Philip A.Nina, AleksandraCattani, CarloNing, BodaCatuogno, LuigiNing, PuqiCausa, FlaviaNing, ZhaolongCavaco, SofiaNino, PasqualeCavagnaro, MartaNiotaki, KyriakiCavalieri, Daniel CruzNishiguchi, HajimeCavallari, Marco RobertoNishino, HideoCavalli, MatthewNishiyama, AkikoCavallo, EugenioNistazakis, Hector ECaven, BarnabyNisticò, NicolaCavenaghi, MarcosNITTA, NaotakaCavoretto, RobertoNitti, MicheleCazorla, MiguelNiu, QinfenCecilio Da Silva Jr, DiógenesNiu, YongCecílio, José (Universidade de Coimbra)Niu, YulingCecílio, José (University of Lisbon)Nivedita, NiveditaCedomir, MilosavlevicNiwano, MichioCegla, FredericNizamov, ShavkatCelaya-Padilla, José M.Njegovec, MatejCelentano, MaurizioNkenyereye, LewisCelesti, MarcoNnaji, ChukwumaCeliktutan, OyaNobile, RiccardoCellini, AntonioNoborio, KosukeCellini, FilippoNobre, Jéferson CamposCellini, NicolaNocerino, EricaCenek, MartinNoels, LudovicCennamo, NunzioNoga, MarcinCenten, PeterNoghanian, SimaCenteno, AnthonyNoh, Yeon SikCenteno, Jorge Antonio SilvaNohama, PercyCenturelli, FrancescoNombona, NolwaziCercenelli, LauraNomikos, NikolaosCerdà-Alabern, LlorençNomura, Ken-ichiCernadas, EvaNonaka, KenichiroCerovski-Darriau, CorinaNones, MichaelCerqueira, EduardoNoori, MohammadCerra, DanieleNorek, MałgorzataCerro, GianniNorgia, MicheleCervantes, JairNorman, RosemaryCesarini, MirkoNosrati, MehdiCespedes, SandraNouretdinov, IliaCestari, ManuelNouri, HassanCetó, XavierNoussan, MichelCeyssens, FrederikNovac, Bucur-MirceaCha, Shi-ChoNovak, EdCha, Sung WoonNovák, JiříCha, Young-JinNovelli, AntonioCha, YoungsuNovellino, AlessandroChadli, MohammedNóvoa, X. RamónChai, DengfengNowak, AleksanderChai, QuanNowak, MateuszChai, Young HoNowakowski, ArturChakraborty, ImonNowicki, MichalChakrapani, SunilNowicki, MichałChalah, Moussa AntoineNowicki, Michał R.Chalioris, ConstantinNowosielski, AdamChalopin, ClaireNozick, VincentChamoso Santos, PabloNozzoli, FrancescoChamoso, PabloNtalampiras, StavrosChamroukhi, FaïcelNtotsios, EvangelosChan, Chia-TaiNtouskos, ValsamisChan, K.L.Nugent, KennethChan, Kam Wai CliffordNugroho, Ferry Anggoro ArdyChan, Keith C. C.Nunes, FernandoChan, PakNuñez, NeftaliChan, RosaNuñez-Andrés, AmparoChan, Yu-WeiNúñez-Valdez, Edward R.Chander, HarishNuno M, GarciaChandrasekaran, ArvindNunziata, FerdinandoChang, Chao-TsunNurmi, JariChang, Cheng-YuanNurse, Jason R. C.Chang, Chia-ChenNussbaumer, AlainCHANG, Chia-MingNuster, RobertChang, Chih-WeiNuti, CamilloChang, Chih-YuanNwaboh, Javis AnyangweChang, Chih-YungNyka, KrzysztofChang, Ching-LungNykiel, GrzegorzChang, GuobinO.V., KononenkoChang, Guo-EnO’Byrne, MichaelChang, Hsiu-WenO’Loughlin, DeclanChang, Hsueh-HsienO’Rorke, RichardChang, IPinOberweger, MarkusChang, Jen-Yuan (James)O'Brien, Megan K.Chang, Ju YongObrzut, JanChang, JunObst, MarcusChang, Kai-ChunOchieng, Washington YottoChang, Kang-MingOchoa, XavierChang, Kuo-JenOchoa-Brust, AlbertoChang, LingOchoa-Ruiz, GilbertoChang, Min-KuanOchoa-Zezzatti, AlbertoChang, Pao-ChiO'Connell, Jessica L.Chang, Pei-ZenOdelius, JohanChang, Ren-JungOdijk, DennisChang, T.COdille, FreddyChang, TianyingOdone, FrancescaChang, VictorOdziemczyk, WaldemarChang, Wan-JungOgashawara, IgorChang, Won-DuOgras, UmitChang, XiaojunOguchi, TakashiChang, Yeong-HwaOgundile, Olayinka O.Chang, Yuan-Jen (Feng Chia University)Oguntala, GeorgeChang, Yuan-Jen (National Yunlin University of Science and Technology)Oh, Beom-SeokChang, Yu-ChungOh, HoonChang, ZhengOh, Je HoonChangqing, ShenOh, Jeong-HoChanover, NancyOh, JuntaekChao, Calvin Yi-PingOh, Ki YongChao, HaiyangOh, MinseokChapeleau, XavierOh, SeungminChapman, RyanOh, TaekeunChappel, EricOh, Tong InChapron, BertrandOh, YohanCharlton, Peter ChristopherOhashi, HirokiCharrier, JoelÖhberg, FredrikCharvátová, HanaOhno, SeigoChatterton, StevenOhta, MaiChaturvedi, PracheeOhtani, ToshihiroChatzichristidi, MargaritaOikonomou, KonstantinosChatzigiannakis, IoannisOjarand, JaanChau, Kwok-WingOjaroudi Parchin, NaserChaudet, ClaudeOjeda, Dora LuzChaudhary, AnkitOjo, MikeChauhan, MunishOjovan, Michael IChauhan, VedangOkabe, Eduardo PaivaChauhan, Vedang DilipkumarOkamoto, HiroyukiChaumette, EricOkamoto, ShogoChauvet, MathieuOkarma, KrzysztofChava, Rama KrishnaOkazaki, ToshiyaChavez, MarioOkeyo, George OnyangoChawathe, Sudarshan S.Okochi, MinaChe, EzraOku, HiromasaChe, TaoOku, YoshitakaChe, WenjieOkumura, SusumuChehri, AbdellahOkunkova, Anna A.Chemura, AbelOláh, LászlóChen (Richard), LiOlaru, AndreiChen, AlbertOlasupo, Tajudeen O.Chen, AssafOlatunbosun, Oluremi AyotundeChen, BadongOlazagoitia, José LuisChen, BaiOlesberg, JonathonChen, BaifanOletic, DinkoChen, BaojunOliphant, AdamChen, BinlingOliva, GabrieleChen, BinqiangOliveira Santos, Lino DeChen, Bo-HaoOliveira, AnabelaChen, Chao (China)Oliveira, Andre Schneider DeChen, Chao (USA)Oliveira, HorácioChen, Chen (Chongqing University)Oliveira, João FradinhoChen, Chen (USA)Oliveira, José M.Chen, Chen (Xidian University)Oliveira, LuísChen, ChiOliveira, RicardoChen, ChiachungOliveira, Sérgio C.Chen, Chi-ChangOliver, SergioChen, Chi-ChihOliveri, GiacomoChen, Chien-ChangOlivieri, LorenzoChen, Chi-Hua (Fuzhou University)Oller, JoaquimChen, Chin-LingOlmi, RobertoChen, ChunO'Loughlin, DeclanChen, Chun-ChiOloumi, DanielChen, Chung-HaoOlsen, Michael J.Chen, CunjianOlsen, SorenChen, DechaoOlson, Michael W.Chen, DeJiuOltean, MihaiChen, Der-ChinOlthuis, WouterChen, Dyi-ChengOlvera-López, ArturoChen, FangO'Mahony, ConorChen, FangjiongOmanović, DarioChen, FengOmar, Mohammed A.Chen, FulongOmetov, AleksandrChen, GaojieOmura, YasuhisaChen, HaiguangOn, Byung-WonChen, HanchiOnaka, SusumuChen, HaoyaoO'Neil, GlenChen, HongyangO'neil-Dunne, JarlathChen, HouyangO'Neill, ShalephChen, Hsing-ChungOng, Keat GheeChen, I-TeOniga, FlorinChen, Jen-JeeOniga, Valeria-ErsiliaChen, JiaOnireti, OluwakayodeChen, JiahongOno, KanjiChen, JianOno, KazuoChen, JianhuiOnofri, Fabrice R.A.Chen, Jian-JiaOñoro-Rubio, DanielChen, Jian-LianOo, Thant ZinChen, JiawangOommen, ThomasChen, JiaweiOoms, MatthewChen, JiayuOpara, KarolChen, JieOpromolla, RobertoChen, JimingOprzędkiewicz, KrzyszofChen, JiucunOrciuoli, FrancescoChen, Jun (China)Ordoñez Morales, Francisco JavierChen, Jun (USA)Ordóñez, CamiloChen, Jung-HsuanOrdoñez, CelestinoChen, JunpingOrduña, FelipeChen, KaifeiOrglmeister, ReinholdChen, Kai-ShengOrio, NicolaChen, KeOrjales, FelixChen, KongzheOrlandi, AntonioChen, Ko-ShaoOrlandi, Marcelo OrnaghiChen, Kuang-ChiOrlandi, SilviaChen, KunshanOrlando, CalogeroChen, LeiOromiehie, EbrahimChen, Liang (Canada)Orosz, GyörgyChen, Liang (China)Orosz, TamásChen, Liang-BiO'Rourke, SharonChen, Lien-WuOrović, IrenaChen, LinglingOrozco Barbosa, LuisChen, LingxinOrozco, JahirChen, LiwenOrozco, UlisesChen, Min (Canada)Orrite-Uruñuela, CarlosChen, Min (China)Orso, ValeriaChen, Mu-SongOrtega, Andrés MuñozChen, PengOrtega, JulioChen, PengpengOrtega, SamuelChen, PinyuOrtega-Zúñiga, Carlos A.Chen, RanOrtis, AlessandroChen, RogerOrtiz Salazar, AndresChen, RuiminOrtiz, AlfredoChen, Shi-HuangOrtiz, JesúsChen, Shih-YuOrtiz, JordiChen, ShuisenOrtiz, JorgeChen, Shuo-HanOrtolani, MarcoChen, SiOrtolani, MicheleChen, SongyueOrts-Escolano, SergioChen, TaoOrue, AmaliaChen, Tei-ChenOrus Perez, RaulChen, TiOsaba, EnekoChen, Tsung-ChiaOsadebey, MichaelChen, Tsung-LinOsipov, VladimirChen, WeiOsório, Jonas HChen, WeiminOssa, Luis Fernando CastilloChen, Wei-TingOsseiran, AdamChen, WenxiOstapowicz, KatarzynaChen, Whai-EnOstasevicius, VytautasChen, WuÖsterberg, PatrikChen, XianfengOstermann, JörnChen, XianzhongÖstlund, BrittChen, XiaoliangOstovar, SaeidehChen, XiaolongOstrowska, KseniaChen, XiaomingOsuagwu, BethelChen, XigaoOsuch, TomaszChen, XiyuanOsuna-Coutiño, J. A. De JesúsChen, XuefengOszczak, BartlomiejChen, XuejinOszust, MariuszChen, XueminOszutowska-Mazurek, DorotaChen, Xun (Canada)Otamendi, Francisco JavierChen, Xun (China)Otebolaku, AbayomiChen, YanOtero, JorgeChen, YangOtero, PabloChen, YaofeiOton, ClaudioChen, Yen-DaOton, Claudio J.Chen, Yen-LinOtoum, SafaChen, Yeou-JiunnOtremba, ZbigniewChen, YewangOtto, TaunoChen, YingOttosen, Carl-OttoChen, YingnongOu, JianzhenChen, YinshengOu, JinpeiChen, YizhengOu, Ting-ChiaChen, Yuan-HoOu, WeihuaChen, YueOuahabi, AbdeldjalilChen, Yu-HsuanOUAKAD, HASSENChen, YunhanOuali, MohammedChen, YuwenOuda, AbdelkaderChen, ZengpingOudre, LaurentChen, ZengshunOueslati, WidedChen, ZeyuOuhyoung, MingChen, ZhengchuanOuyang, BinChen, ZhenghuaOvaliadis, KyriakosChen, ZhigangOviedo-Trespalacios, OscarChen, ZhiminØvstedal, OlaChen, ZhiqiangØvsthus, KnutChen, ZhitongOwerko, TomaszChen, ZongHaiOwn, Chung-MingCheneler, DavidOzen, MehmetCheng, BinOzer, EkinCheng, ChengOzkan-Aydin, YaseminCheng, Chen-YangPaasio, AriCheng, ChiPabinger, ChristofCheng, Chia-Chi ChengPabst, OliverCheng, Chia-HsinPachauri, VivekCheng, Chiang-HoPacheco, Jorge FuentesCheng, Chih-ChiaPaciello, VincenzoCheng, Chin-ChiPadalkar, MugdhaCheng, EnPaderewski, PatriciaCheng, Fang (China)Padilha Lanari Bo, AntonioCheng, Fang (Singapore)Padilla, PabloCheng, GongPadois, ThomasCheng, Hsin-Yi KathyPadua, LuisCheng, HuanyuPaduan, JeffCheng, Huanyu (Larry)Paek, JeongyeupCheng, JianhuaPaeng, Dong-GukCheng, JunPagano, GuidoCheng, Jun-HuPage, AlexCheng, LishengPagna Disso, JulesCheng, NanPaipulas, DomasCheng, PengPaiva, CassioCheng, QiangPaiva, SaraCheng, ShiPaixão, Thiago R.L.C.Cheng, Shing ShinPajor, MirosławCheng, ShunfengPak, SoojongCheng, Wei ChenPakrashi, VikramCheng, WeiyingPal, AmitangshuCheng, William Y. Y.Pal, KamalenduCheng, Wood-HiPal, SudeshnaCheng, XianghongPalacios, AntonioCheng, YongqiangPalacios-Laloy, AgustinCheng, ZhenzhouPalacz, MagdalenaCheng, ZhiqiangPalade, AndreiCheong, Joon WaynPalanisamy, SelvakumarChernyi, SergeiPalattella, Maria RitaChessa, ManuelaPalazzi, ValentinaChessa, StefanoPalczynska, BeataCheung, WaimingPalego, CristianoCheval, SorinPalella, Boris IgorChew, Zheng-JunPalermo, EduardoChhatkuli, AjadPalestra, GiuseppeChhetri, Parveen KumarPaleu, ViorelChi, Yung-WeiPalinko, IstvanChiadò, AlessandroPałka, NorbertChiang, C. C.Palla, MirkóChiang, Chen-KuoPalleschi, VinceChiang, Chia-ChinPallotta, LucaChiang, Hai-PangPallottino, FedericoChiang, Kai-WeiPalma, DavidChiariotti, FedericoPalma, LorenzoChiariotti, PaoloPalmeri, RobertaChiavaioli, FrancescoPalmerini, GiovanniChiba, TakashiPalneedi, HaribabuChicea, DanPalombi, LorenzoChien, Feng-TsunPalus, HenrykChien, Hung-YuPalys, BarbaraChien, Jun-ChauPalzer, StefanChien, Nguyen BaPamies, RamonChien, Ying-RenPamplona Segundo, MaurícioChikurtev, DenisPamučar, DraganChileshe, NicholasPamuła, TeresaChillara, Vamshi KrishnaPamula, V RajeshChilson, PhilPamuła, WieslawChin, Cheng SiongPan, Ci-LingChindamo, DanielPan, CunhuaChing, Congo Tak ShingPan, DongChing, Tak-ShingPan, DonghuaChinnadayyala, Somasekhar R.Pan, Huang-HsingChintakunta, HarishPan, JiayiChinthammit, WinyuPan, PengminChiodini, SebastianoPan, ShijiaChiorcea-Paquim, Ana-MariaPan, TianhongChiriacò, Maria SerenaPan, WeifengChis, VasilePan, YunChitale, KedarPan, ZhaoqingChiti, FrancescoPan, ZhigangChiu, Chien-KuoPan, ZhiwenChiu, Nan-FuPan, ZongxuChiu, Tai-ChiaPanagiotakis, SpyrosChiu, Te-WeiPanagopoulos, AthanasiosChiu, Wing KongPanchal, SatyamChizari, HassanPancheri, LucioCho, ByungjinPanda, ManojCho, ChungyeonPander, TomaszCho, ChunheePandey, GauravCho, Dang-ilPandith, AnupCho, Dong-HoPanduro, Marco A.Cho, Eun JeongPandya, AbhilashCho, GihwanPaneiro, GustavoCho, Ho-ShinPanero, ElisaCho, MinkyuPang, GuangchangCho, SoohyunPang, YoonsooCho, SooyeonPang, ZhiboCho, Sung-BaePangaluru, KishoreCho, Young ImPani, DaniloCho, YoungjunPannek, CarolinChoi, AhyoungPannocchia, GabrieleChoi, ByungjooPanowicz, RobertChoi, Cheon WonPantano, PaulChoi, Han-LimPantazi, Xanthoula EiriniChoi, HeungjaePantazis, GeorgeChoi, Heung-JinPantea, CristianChoi, HojongPantelic, JovanChoi, Hyouk RyeolPanthi, DhrubaChoi, JaehyukPantoli, LeonardoChoi, JaesoonPanzardi, EnzaChoi, JaewanPaolanti, MarinaChoi, Jane RuPaolicelli, FedericaChoi, Jee WoongPaolini, ChristopherChoi, Jeong-WooPaolini, RiccardoChoi, JihoonPaolo, BollellaChoi, Jin-GhooPaolo, FavaliChoi, Jin-KyuPaolozzi, AntonioChoi, Ji-WoongPaone, NICOLAChoi, JiyeonPap, LászlóChoi, JungHunPapa, PaoloChoi, JungilPapadakis, Nikolaos M.Choi, KyoungahPapadimitriou, KimonChoi, Kyoung-HoPapadopoulos, TheofilosChoi, MinhaPapageorgas, PanagiotisChoi, MyungjoonPapakostas, George A.Choi, OukPapari, Gian PaoloChoi, Seung-bokPapathanassiou, ApostolosChoi, SeungkeunPapatsimpa, CharikleiaChoi, SungpillPapi, EnricaChoi, SunwoongPappalardo, LucaChoi, Woo JunePappas, NikolaosChoi, WooyeolParada Medina, RaúlChoi, YongkiParaforos, Dimitrios S.Choi, YosoonParaschiv-Ionescu, AnisoaraChoi, YoungcholParedes-Madrid, LeonelChoi, YukyungParedes-Trejo, FranklinChoisne, JulieParente, ClaudioChomiak, TaylorPareschi, FabioChong, IlyoungParia, DebadritaChong, Kwen SiongParian, MehdiChong, PeterParida, Bikash RajanChong, See YennParigger, ChristianChoo, Pei LingPariota, LuigiChorsi, Meysam T.Parisotto, SimoneChortos, AlexPark, ByeongjinChou, Hsi-TsengPark, Byung-WookChou, Jung ChuanPark, ByungwoonChou, Tien-YinPark, ChansikChou, Tse HengPark, Choon-SuChou, Tzu-ChiehPark, Deog-SuChou, WushengPark, DongwookChoudhury, SalimurPark, HanjungChouhan, Shailesh SinghPark, HeunChoukulkar, AdityaPark, Ho-HyunChow, Chi-WaiPark, HongsikChowdhury, DhimanPark, Hyun JinChowdhury, MorshedPark, JaehyunChowdhury, SourangsuPark, Jee WoongChristian, John A.Park, JeonghunChristodoulos, ChamzasPark, JinhyoungChristopoulos, GeorgiosPark, Jong-JinChristopoulou, EleniPark, JongwoongChronopoulos, DimitriosPark, JoonChrpa, LukasPark, JooyoungChrysikou, LoukiaPark, Jung-DongChu, HairongPark, Kang RyoungChu, JinkuiPark, KeeHyunChu, Shu-ChuanPark, KeunhyunChua, BeeleePark, Kwan KyuChuang, Cheng-HsinPark, Kwi-IlChuang, Chun-HsiangPark, Kwon GyuChuang, Kuo-ChihPark, Kyoung YoulChuang, Yung-ChungPark, KyungWonChui, Hsiang-ChenPark, Man-BokChui, Kwok TaiPark, MinChukin, VladimirPark, MyunghwanChum, JaroslavPark, PangunChun, HongguPark, SangjoonChun, SejongPark, SehyunChunchuzov, IgorPark, Seong-OokChung, Hyun-JoongPARK, SEUNGHEEChung, IlyongPark, SeungkyungChung, Ren-JeiPark, SimonChung, SungwonPark, SookukChung, Tein-Yaw DavidPark, Soon-YongChung, Vincent PeyPark, SuhyunChung, Yao-LiangPark, SukyungChung, YongwhaPark, UnsangChurch, IanPark, Won KwangChvojka, PetrPark, Yeong DonChybowski, Leszek MarekPark, YeonjeongCiabatta, LucaPark, YongbaeCiaccheri, LeonardoPark, YongKeunCiampa, FrancescoPark, Young IlCiampalini, AndreaPark, YoungsooCiampi, SimoneParmehr, Ebadat GhanbariCiampolini, PaoloParnandi, AvinashCianca, ErnestinaParra Boronat, LorenaCianflone, GiuseppeParra Cuentas, Edwin RogerCiarfuglia, Thomas A.Parra, LorenaCicchetti, RenatoParrein, BenoîtCiccognani, WalterParrington, LucyCiccone, MarcoParšeliūnas, EimuntasCicconetti, ClaudioPartovi-Nia, RachelCichella, VenanzioParts, LeopoldCicirelli, FrancoPartsinevelos, PanagiotisCidronali, AlessandroParunov, JoskoCiecholewski, MarcinParupudi, TejasviCieplak, MaciejParvez, ImtiazCięszczyk, SławomirParviainen, JaanaCifredo-Chacón, María-ÁngelesParziale, AntonioCifuentes, Carlos A.Pascal, CarlosCigáň, MarekPascali, Maria AntoniettaCigna, FrancescaPascoli, Stefano DiCilfone, AntonioPascucci, SimoneCillo, FabrizioPashami, SepidehCimalla, VolkerPasinszki, TiborCiminelli, CaterinaPasluosta, CristianCinel, CaterinaPasolini, GianniCiobanu, Radu IoanPaspallis, NearchosČiplys, DaumantasPassalis, NikolaosCiprian, DaliborPasserone, RobertoCipriani, ChristianPassian, AliCirillo, FlavioPassos, DiegoCisneros, RafaelPasternak, GrzegorzCiuborauru, FlorinPasternak, MateuszCiuc, MihaiPastor, DanielCiuciu, IoanaPastore, PaoloCiuffoletti, AugustoPastorelli, StefanoCiuonzo, DomenicoPatanè, FabrizioCivitarese, GabrielePatchay, SandhiranClaesen, LucPatel, PankeshClaeys, KurtPatel, RikeshClamor, AnnikaPaternò, FabioClare, Richard MichaelPati, AshisClark, JasonPatias, PetrosClarke, ThomasPatil, DevendraClaudio, SavaglioPatil, MadhavClaussen, JonathanPatra, Hirak KCleland, IanPatra, SubirClement, PierrickPatriciu, Victor-ValeriuClemente, Carmine StefanoPatrick, ErinClemente, FrancescoPatroi, DeliaClementi, FrancescoPatrone, FabioClementini, EliseoPatterson, MatthewCleveland, Robin O.Patwardhan, AmolClevenger, Kimberly AnnPatzold, StefanClimente-Alarcon, VicentePau, AnnamariaCliment-Pérez, PauPau, GiovanniClose, Dan M.Pau, Louis-FrancoisCmiljanic, NikolaPaudel, BasantaCobianu, CornelPauk, JolantaCoburn, Craig A.Paul, AnandCocovi Solberg, David JaimePauliukaite, RasaCocuzza, SilvioPaulo, João RuivoCodetta Raiteri, DanielePaulus, StefanCodetta-Raiteri, DanielePaunescu, GabrielaCoelho Silva, MateusPavan, Esteban EnriqueCoelho, JoãoPavan, GabrieleCoelho, João PintoPavan, Theo ZeferinoCoelho, Luís CarlosPavanello, FabioCoelhoso, IsabelPavelyev, VladimirCoenen, SebastianPavesi, MauraCoffetti, DennyPavlidis, IoannisCogato, AlessiaPavon-Djavid, GracielaCognetti, MarcoPAW, Yew ChaiCohen, Jason BlakePawlak, RyszardCohen, RamiPawłowicz, BartoszCointault, FrédéricPawłowski, EligiuszCoisson, MarcoPawłowski, PawełCojocaru, AncaPayá, LuisCola, GuglielmoPayandeh, ShahramColella, RiccardoPayo, IsmaelCollart-Dutilleul, Pierre-YvesPayrebrune, KristinCollazos, CesarPeacock, Oliver J.Collins, CassandraPecorella, TommasoColombatti, GiacomoPecori, RiccardoColombo, ClaudioPecunia, VincenzoColonnese, StefaniaPeddigari, MaheshColosi, FrancescaPedersen, SimonColosimo, GabrielePedersini, FedericoColuccia, AngeloPediaditis, MatthewColusso, ElenaPedreño-Molina, Juan L.Comai, SaraPedrero González, AntonioComan, CristinaPedrielli, FrancescaCombrisson, ÉtiennePedrosa, Eurico FarinhaComeau, FrankPeerTole, PeterComi, AntonioPegorini, ViníciusCominelli, LorenzoPeham, ChristianComisso, MassimilianoPehl, MichaelComite, DavidePei, WeihuaConceição, RaquelPei, ZhichaoConci, NicolaPei, ZingwayConcu, GiovannaPeimankar, AbdolrahmanCondino, SaraPeiner, ErwinConforti, MassimoPeirano, AndreaConmy, RobynPeixeiro, CustodioConnolly, JamesPeláez, GerardoConoci, SabrinaPeláez-Moreno, CarmenConsel, CharlesPelegrí, JoséConteduca, DonatoPelivanov, IvanConti, VincenzoPellegrini, NicolaContreras Castillo, JuanPellenz, Marcelo EduardoContreras-Medina, LuisPelton, Joseph N.Contreras-Ortiz, Sonia HelenaPeltoniemi, JouniConzuelo, FelipePelz, Jeff B.Cooke, CoreyPeña Pitarch, EstebanCooper, Matthew A.Penate-Sanchez, AdrianCoors, VolkerPeng, ChuangCopic, DavorPeng, DengfengCopot, CosminPeng, GaoliangCoppens, BartPeng, JianCoppola, SaraPeng, JianqingCoraddu, AndreaPeng, LihuiCorchado Rodríguez, Juan ManuelPeng, LinningCorchia, LauraPeng, PinganCorcoran, PeterPeng, XiCorcovilos, Theodore A.Peng, XingchaoCordella, FrancescaPeng, YankunCordera, RubénPeng, Yan-TsungCordioli, JulioPeng, Ying-JieCordoba, RicardoPeng, YongCorigliano, AlbertoPeng, ZhengCorke, PeterPeng, ZhengyuCornish, SimonPeng, ZhenmingCorrales Ramon, Juan AntonioPennazza, GiorgioCorrea-Pugliese, Claudia V.Pensieri, SaraCorreia, SérgioPepe, AntonioCorsi, Marie-ConstancePepe, GianlucaCorsini, MassimilianoPepe, MassimilianoCortés, Araceli GonzálezPepe, MonicaCortez González, JoaquinPepe, SusiCoscetti, SimonePeppas, KostasCosentino, LuigiPeppel, TimCoskun, M. BulutPerazzo, PericleCosoli, SimonePercus, AllonCossel, KevinPereira, AntónioCossu, MarcoPereira, FelisbertoCosta Ramos, DanielPereira, LucasCosta, AngeloPereira, Luiz Fernando RibeiroCosta, CorradoPereira, NunoCosta, Daniel G.Pereira, SandraCosta, JoaoPerelli, AlessandroCosta, Manuel Filipe P. C. M.Peremans, HerbertCostantino, DomenicaPérennou, M. AndréCostanzo, AmbrogioPerera, RicardoCostanzo, CarmelinaPerera, SudanthaCostanzo, SandraPerera, ThusharaCosta-Rama, EstefaníaPeres, António M.Costa-Requena, JosePeres, EmanuelCoste, JérômePeres, Henrique E. M.Costello, Ben De LacyPereverzyev, SergeiCostin, AaronPérez de Prado, Rocío JosefinaCostin, Andrei (Finland)Perez Herrera, Rosa AnaCostin, Andrei (France)Pérez Jiménez, RafaelCota, GiuseppePérez Menéndez, Ramón JoséCoto García, BaudilioPérez Oria, JuanCouper, KeithPérez Rodríguez, NicolásCourjal, NadègePérez Turiel, JavierCourtier-Murias, DenisPérez, Agustın-AgüeraCoutrot, AntoinePérez, Alfredo J.Coviello, GiuseppePérez, Eduardo CasilariCovington, JamesPérez-Grande, IsabelCozza, AndreaPérez-López, CarlosCozzolino, DanielPérez-Martín, EnriqueCraciunescu, RazvanPérez-Meana, Héctor ManuelCraddock, MattPerez-Navarro, AntoniCragg, PeterPérez-Ocón, FranciscoCrescentini, MarcoPerez-Ramirez, CarlosCrescitelli, AlessioPérez-Rodríguez, FernandoCrespo Bofill, PedroPérez-Serrano, AntonioCrespo, Gastón APérez-Suay, AdrianCrespo, JonathanPergola, NicolaCrespo, Rubén GonzálezPerikos, IsidorosCretaux, Jean-FrancoisPerilli, StefanoCretu, Ana-MariaPeris-Lopez, PedroCrétual, ArmelPerkins, NoelCrippa, PaoloPerković, ToniCrisan, Gloria CeraselaPernau, Hans FridtjofCristea, CeciliaPeron, RobertoCristea, Mihaela DanaPerotoni, Marcelo BenderCristiani, PierangelaPerra, CristianCristina, Gaitan NicoletaPerret, EtienneCrivelli, Davide (Italy)Perrone, GuidoCrivelli, Davide (UK)Perry, PhilipCrivello, AntoninoPerry, StuartCrocco, LorenzoPerš, JanezCroce, Anna CletaPersad, Ravi AncilCroce, PierpaoloPersaud, ArunCrooks, GeorgePersico, RaffaeleCross, AdamPersijn, StefanCrouter, Scott E.Persson, AndersCrovetti, Paolo StefanoPertile, MarcoCruciani, FedericoPerton, MathieuCruvinel, Paulo E.Pertusa, AntonioCruz, Homero ToralPesavento, MariaCruz, PedroPescapè, AntonioCruz, SandraPescaru, DanCruz-Delgado, Víctor JavierPesce, GiuseppeCruz-Villar, Carlos-AlbertoPesch, DirkCsapo, AdamPescosolido, LoretoCsősz, ÉvaPessin, GustavoCsurgai-Horváth, LászlóPetcu, DanaCtistis, GeorgiosPeter, LukášCuartero, MaríaPeter, SteffenCuccoli, FabrizioPeters, KaraCuesta, EduardoPetersen, JanCuevas Martínez, Juan CarlosPeterson, JohnCui, ChangcaiPetit, NicolasCui, HuaqingPetkie, DouglasCui, JingnanPetko, MaciejCui, JinhuaPetlicki, MichalCui, JiwenPetošić, AntonioCui, LaizhongPetraglia, Mariane R.Cui, LongjiPetraki, ErmioniCui, RongxinPetráková, VladimíraCui, XiaohuiPetrellis, NikosCui, YaoyaoPetrescu, Florian Ion TiberiuCui, YuPetrich, JanCui, ZhaopengPetropulu, AthinaCui, ZhengPetrosanu, Dana-MihaelaCui, ZhihuaPetrov, DmitryCui, ZhisongPetrović, JovanaCui, ZiqiangPetrovski, AndreiCuiñas, IñigoPetrů, MichalCukurova, MutluPetrucha, VojtěchCullins, Miranda J.Petry, MarceloCulshaw, BrianPetry, MirtaCulver, R. LeePetsa, ElliCummins, NicholasPeyrodie, LaurentCunha, AntónioPeysakhovich, VsevolodCunha, MarioPeyskens, FrédéricCuriac, Daniel-IoanPezzimenti, FortunatoĆurković, Helena OtmačićPezzo, GiuseppeCvelbar, UrosPezzuolo, AndreaĆwiąkała, PawełPfattner, RaphaelĆwiklak, JanuszPfeffer, KarenCyganek, BogusławPfitzner, ChristianCzaja, ZbigniewPflanz, MichaelCzajewski, WitoldPham, ChuanCzaplewski, KrzysztofPham, Duc TruongCzaplik, MichaelPham, Quoc-VietCzyżewski, AndrzejPham, Thinh HungD. Engeberg, ErikPham, Tien DatD’Amico, ArnaldoPham, Tuyen DanhDa Costa, CesarPhan, Hoang-PhuongDa Cunha, Felipe DomingosPhilippe, JulienDa Gama, BernardoPhilipsen, Mark PhilipDa Silva Barros Allil, Regina CéliaPhung, ToanDa Silva Camargo, SandroPiacentini, DanielaDa Silva E Silva, Francisco JoséPianini, Daniloda Silva, Jean Carlos CardozoPiano, MartinaDa Silva, José MachadoPiano, SamantaDa Silveira Petruci, João FlávioPiao, XianjiDa Xu, LiPiasecki, MichaelDabove, PaoloPiccioni, Mario DanieleDąbrowska, DominikaPiccoli, FlavioDabrowski, JoelPicerno, PietroDabrowski, Pawel S.Picó, RubénDabrowski, PiotrPicollo, FedericoD'Addona, Doriana MarilenaPicone, MarcoDaeyoung Kim, DaeyoungPiekarz, IlonaDagdeviren, CananPieraccini, MassimilianoDagdeviren, OmurPieralice, FrancescaDagliati, AriannaPierdicca, RobertoDai, DongkaiPieren, RetoDai, HaipengPieri, GabrieleDai, Henry Hong-NingPierleoni, PaolaDai, HoudePierściński, KamilDai, Jian-GuoPierucci, LauraDai, JianmingPietrosemoli, ErmannoDai, RuiPietrusewicz, KrzysztofDai, WeiPilan, LuisaDal Ben, DiegoPiłat, AdamDalcé, RéjanePilát, ZdeněkDaliakopoulos, IoannisPiletsky, SergeyDalla Betta, Gian-FrancoPilla, VivianeD'Alonzo, MarcoPillai, PadmanabhanD'Alvia, LivioPillet, HeleneDam, BernardPilot, RobertoDam, Van Anh T.Piltan, FarzinDamasevicius, RobertasPimenta, Tales CleberDamaševičius, RobertasPina, PedroDamiani, LeonardoPinchera, DanieleD'Amico, FabrizioPineda-Sanchez, ManuelDamjanović, DomagojPing, JianfengDandois, Jonathan P.Pingarrón, JoséD'Andon, Odile FantonPinhasi, YosefD'Andrea, AndreaPinho, DianaD'Andreagiovanni, FabioPinho, HenriqueDaneault, Jean-FrancoisPinho, PedroDanescu, RaduPinho, Tatiana M.Danezis, ChrisPinotti, Cristina MariaDang, Thanh DucPinto, Alex Sandro RoschildtDang, TuanPinto, JamesDang, VinhPinto, MónicaDaniel, BillPintore, GiovanniDaniele, MichaelPintus, RuggeroDanieletto, MatteoPio Belfiore, NicolaDanielli, AmosPiotr, JaworskiDanner, MartinPiotrowski, ZbigniewDanoy, GrégoirePiotto, MassimoDantan, AurélienPirbhulal, SandeepDantas, Mario A.R.Pires, GabrielDao, Nhu-NgocPires, IvanDao, Phong B.Pires, Ricardo HugoDao, Thanh-PhongPirinen, PekkaDaout, SimonPirjan, AlexandruDapino, MarceloPirmagomedov, RustamDarabi, AliPiro, BenoitDardano, PrincipiaPirouz, MatinDardari, DavidePirri, FioraDarkó, ÉvaPisana, SimoneDarrah, MarjoriePischiutta, MartaDarrozes, JoséPishchalnikov, Roman Y.Darsena, DonatellaPislaru-Danescu, LucianDarties, BenoîtPisoni, EnricoDarvishian, AliPitarch, JaimeDas Bhattacharjee, SreyaseePitarma, Rui AntónioDas, AbhijitPitchappa, PrakashDas, AnirbanPiteľ, JánDas, JagotamoyPitkethly, AmandaDas, PradipPitla, SantoshDasanna, Anil K.Pitombeira-Neto, Anselmo RamalhoDasgupta, AnuragPittino, FedericoDaszczuk, Wiktor B.Piuzzi, EmanueleDatta, DebopamPivarčiová, ElenaDatta, KaustuvPivonka, PeterDatta, RahulPiyare, RajeevDatta, RupsaPiyathilaka, LasithDatta, SumonPizarro, FranciscoDatta, SuprakashPizkozub, JacekDau, Van ThanhPizzeghello, DiegoDaus, AlwinPizzi, SaraDautov, RustemPizzolante, RaffaeleDavalli, AngeloPizzonia, MaurizioDávid, BencePizzotti, MatteoDavid, SchugPizzuti, StefanoDavids, CorinePłaczek, BartłomiejDavidson, AndrewPlaczek, MarekDavidson, DrewPlatero, Carlos ADavis, ClairePlatil, AntoninDavoli, LucaPławiak, PawełDavoudi, AnisPletersek, AntonDawe, Robert J.Pletl, SzilveszterDawidowicz, KarolPlets, DavidDawn, ArnabPleva, MatúšDawy, ZaherPlusquellic, JimDe Aguiar, Claudinei RodriguesPlutino, Maria RosariaDe Almeida Teixeira, Gustavo HenriquePnevmatikos, NikosDe Almeida, José Manuel M.M.Pôças, IsabelDe Angelis, Alessio (Italy)Podd, Frank J.W.De Angelis, Alessio (Sweden)Podnar, SimonDe Angelis, GuidoPodobnik, JanezDe Battista, NicholasPodoliak, NinaDe Benedictis, AlessandraPodpečan, VidDe Berardis, DomenicoPodržaj, PrimožDe Beule, PieterPodsędkowski, Leszekde Blasio, Gabriele S.Poel, MannesDe Boer, LisannePoenar, DanielDe Castro Andrade, Rossana MariaPoggi, ValerioDe Castro, AlbertoPohanka, MiroslavDe Castro, Miguel FranklinPohjankukka, JonneDe Celis, RáulPoint, DavidDe Cesare, FabrizioPokorný, PetrDe Cooman, ThomasPolak, LadislavDe Diego, Isaac MartínPolanský, RadekDe Farias, Claudio M.Połap, DawidDe Fátima Domingues, MariaPolatidis, NikolaosDe Freitas, Edison PignatonPolese, Janaíne CunhaDe Gaetani, Carlo IapigePolguj, MichalDe Gracia, PabloPolicelli, FrederickDe Grande, RobsonPolishchuk, YuryDe Iacovo, AndreaPolivka, MilanDe La Cruz, ErnestoPollari, FrancescoDe La Escosura-Muñiz, AlfredoPolley, NabarunDe La Fuente, Jesús GrajalPołom, MarcinDe La Iglesia, Daniel H.Poltorak, LukaszDe La Prieta Pintado, FernandoPolycarpou, Irenede la Torre Diez, IsabelPomante, LuigiDe la Torre Díez, IsabelPomar, JesúsDe La Torre Ibarra, Manuel H.Pomares, SaúlDe La Torre, Gabriel G.Pomorski, DenisDe Lacerda, RaulPonce, HiramDe Lima Monteiro, Davies WilliamPoncela, JavierDe Luca, Anna ChiaraPonchak, George E.De Luca, MichelePoniszewska-Maranda, AnetaDe Maio, VincenzoPonomaryov, VolodymyrDe Marchi, LucaPons, AlexanderDe Medeiros, RicardoPons, EnricoDe Meij, Tim G.J.Pons, PatrickDe Melo, Igor DelgadoPonterio, Rosina CelesteDe Miguel, SilviaPonti, CristinaDe Mingo López, Luis FernandoPontikos, NikolasDe Morais Amory, AlexandrePontin, AntonioDe Moura Ramos, José JoaquimPonzoni Carvalho Chanel, CarolineDe Müllenheim, Pierre-YvesPooranian, ZahraDe Munari, IlariaPop, ClaudiaDe Nardis, LucaPOPA, Călin-AdrianDe Oliveira, Mario AndersonPopa, DanielDe Pauw, EdwinPopa, Gabriel NicolaeDe Poorter, EliPope, Gunnar C.De Sanctis, MauroPopelka, StanislavDe Santis, AngeloPopescu, DanDe Santis, MichelePopescu, Daniela-ElenaDe Silva, VarunaPopescu, DorinDe Simone, Marco ClaudioPopescu, Ionel CatalinDe Smet, Timothy SPopescu, VladDe Sousa Jr., Rafael T.Popielarczyk, DariuszDe Souza Gil, EricPoplawska, MagdalenaDe Souza, Éfren LopesPopleteev, AndreiDe Souza, Eniuce MenezesPorcu, FedericoDe Stefano, LucaPorée, FabienneDe Vito, SaverioPortela, FilipeDe Vos, Bob D.Portelinha, Francisco ManuelDe, SuparnaPorter, EmilyDeakin, MarkPortugal, PauloDean Ben, Xose LuisPosada-Quintero, HugoDean, TimPosada-Quintero, Hugo FernandoDeans, CameronPosada-Roman, Julio E.D'Eath, RickPospichal, BernhardDebela, Ahmed MehdiPospíšilová, MarieDebliquy, MarcPossan, EdnaDebnath, MadhuriPostacchini, MatteoDebono, Carl JamesPostigo, Pablo AitorDebroy, SaptarshiPostnikov, PavelDecroze, CyrilPostolache, OctavianDefraigne, PascalePoston, BrachDegler, David (France)Potamitis, IlyasDegler, David (Germany)Potenza, FrancescoDehais, FrédéricPotter Van Loon, Bert JanDehe, AlfonsPotter, MarkDehghani, MiladPottier, BernardDehghan-Niri, EhsanPotyrailo, RadislavDei, MichelePoulakis, NikolaosDeigner, Hans PeterPound, MichaelDeilami, KavehPourrahimi, Amir MasoudDejam, MortezaPoursanidis, DimitrisDeka, DeepjyotiPourzolfaghar, ZohrehDekorsy, ArminPoux, FlorentDel Barrio, JesúsPovinelli, RichardDel Campo, Francisco JavierPovolotskiy, Alexey V.Del Gaudio, CostantinoPowell, Samuel BearDel Hougne, PhilippPowrie, HonorDel Jesus, ManuelPowrie, WilliamDel Peral-Rosado, José A.Pozo, FrancescDel Pozo, SusanaPozzebon, AlessandroDel Río Celestino, MercedesPozzi Mucelli, StefanoDel Rio Fernandez, JoaquinPrabhakar, AmlenduDel Valle Soto, CarolinaPrabhu, RadhakrishnaDel Valle, ManelPrabowo, Aditya RioDel-Blanco, Carlosr R.Praczyk, TomaszDelcea, CameliaPrajzler, VaclavDelebarre, ChristophePrakash Kottapalli, Ajay GiriDeleforge, AntoinePrakht, VladimirDelepine-Lesoille, SylviePrandi, CatiaDelfino, InesPrasad, AbhnilDelgado Prieto, MiguelPrasad, Dilip K.Delgado, CarmenPrasad, MukeshDelgado, JorgePrasad, ShaliniDelgado-Gonzalo, RicardPrasad, ShitalaDelgado-Pinar, MartinaPrashanth, Konda GokuldossDelibasis, Konstantinos K.Prasser, Horst-MichaelDelicato, FlaviaPrates, GonçaloDelis, AlexPrati, RonaldoDell’Olio, FrancescoPrats Boluda, GemaDelmonico, LucasPredoi, Mihai V.Demartini, MelissaPreethichandra, Daluwathu Mulla GamageDementyev, ArtemPrehofer, ChristianD'Emilia, GiulioPreiner, ReinholdDemir, BilalPremachandra, ChinthakaDemir, NusretPremanode, BhusanaDemirci, StefaniePremaratne, PrashanDemontoux, FrançoisPresto, AlbertDemyanov, VladislavPreuveneers, DavyDen, WalterPrevitali, MattiaDeng, FangPrezioso, GiuseppinaDeng, LeiPribičević, BoškoDeng, QiliangPřibilová, AnnaDeng, QinghaiPricop, EmilDeng, QingxuPridmore, TonyDeng, ShuoPriefer, RonnyDeng, TongPrieto Guerrero, AlfonsoDeng, WuPrieto, CarlosDeng, XianjunPrieto-Castrillo, FranciscoDeng, XinweiPrijić, AnetaDeng, YangfanPrikulis, JurisDeng, YongPrimak, SergueiDeng, ZhiguoPrimpke, SebastianDeng, ZhihongPrince, Gregary B.Deniziak, StanisławPrisacariu, VasileDenoual, MatthieuPriye, AashishDentico, DanielaProcek, MarcinDenton, AnneProchazka, AlesDeo, Randhir P.Prochazka, DavidDepari, AlessandroProchazka, IvanDepolli, MatjažProchazka, MarekDercas, NicholasProcter, JonathanDerhamy, HasanProestos, CharalamposDerlatka, AnnaProfico, AntonioDerlatka, MarcinProkeš, AlešDerrien, MorganeProkhorov, Mikhail Evgen'evichDerrode, StéphanePronello, CristinaDeshpande, KshitijaPronost, GuillaumeDesmaële, DenisProsa, MarioDesmulliez, MarcProto, AndreaDesolda, GiuseppeProvenzano, Frank A.DeSouza, GuilhermeProwell, StacyDestelle, FrancoisPrudenzano, FrancescoDetrembleur, ChristinePRUNEANU, Stela-MariaDeufemia, VincenzoPryamikov, AndreyDevadas, Mary SajiniPryss, RüdigerDevaney, NicholasPrzyborski, MarekDevaurs, DidierPrzybylek, PiotrDevkota, JagannathPrzybyło, JaromirDevost, DominicPrzybyt, MałgorzataDewan, Ashraf M.Psannis, KostasDey, ShuvashisPsarobas, IoannisDhaene, TomPsarras, Georgios C.Dhakal, AshimPsarrou, AlexandraDhakal, RajendraPsimoulis, PanosDhakshinamoorthy, AmarajothiPsota, EricDhaliwal, Gurpinder SinghPsuj, GrzegorzDhanjai, DhanjaiPsychalinos, CostasDhaou, Imed BenPu, CongDherbecourt, Jean-BaptistePu, HaihuiDi Angelo, LucaPu, LinaDi Bartolomeo, AntonioPu, ShengliDi Donato, LoretoPu, XiongDi Flumeri, GianlucaPuchades, IvanDi Leo, GiuseppePuche-Panadero, RubenDi Maio, PaolaPudelko, RafalDi Marco, AlessandroPuente, SantiagoDi Marco, PiergiuseppePuig-Bargués, JaumeDi Marco, RobertoPuiu, MihaelaDi Nardo, FrancescoPujol, ToniDi Natale, GiorgioPukalchik, MariiaDi Nunzio, LucaPuletti, NicolaDi Nuovo, AlessandroPuliafito, AntonioDi Pietrantonio, FabioPulido Fernández, ManuelDi Pino, GiovanniPulinets, SergeyDi Risio, MarcelloPullanagari, ReddyDi Sante, RaffaellaPullano, SalvatoreDi, WangPunch, JeffDiaconita, VladPundlik, ShrinivasDiallo, DembaPunpongsanon, ParinyaDianat, PouyaPuschita, EmanuelDiao, JunmingPuskar, MichalDiao, XiuminPustan, MariusDias, AndrePustišek, MatevžDias, PauloPuthal, DeepakDíaz Nafría, José MaríaPutilov, ArcadyDíaz Redondo, Rebeca P.Puton, JarosławDíaz, CristinaPyattaev, AlexanderDiaz, Jose ManuelPyo, SukhoonDíaz, Julia CalderónPyromalis, DimitriosDíaz, Pilar GarcíaPytharouli, StellaDíaz-Alonso, DanielaPytka, JarosławDiBenedetto, FrancescoPyun, Jae-YoungDiCarlofelice, AlessandroQasha, RawaaDiciotti, StefanoQi, GuanqiuDickert, Franz L.Qi, LongDickey, MichaelQi, XiaozhiDietzel, StefanQi, YanfeiDiez Blanco, Luis EnriqueQian, WeiDíez, Luis FranciscoQian, XinwuDiez, YagoQian, ZhengDigulescu, AngelaQiao, GangDilo, ArtaQiao, JianzhongDilonardo, ElenaQiao, LiangDimitrievski, MartinQiao, YuansongDimitrios, KogiasQiao, ZhijunDimitrios D. Alexakis, Dimitrios D. AlexakisQin, DanyangDinardo, SalvatoreQin, FeiDinc, ErginQin, HongdeDing, BaiyuanQin, JieDing, EnjieQin, LingqiaoDing, FengQin, ShiqiaoDing, GuoruQin, TaoDing, JianguoQin, XuleiDing, JianliQin, YiDing, MengQin, YihengDing, ShihongQingyuan, ZhuDing, WenboQiu, SenDing, XiaorongQiu, TieDing, YongQiu, XianboDing, YuanQiu, YongqiangDing, YueminQiu, ZhenDing, ZhaoyangQu, FeifeiDing, ZhenyangQu, FengzhongDingler, TilmanQu, HangDingwell, JonathanQu, HongweiDinh, AnhQu, XiaoleiDinh, Ngoc ThanhQu, YonghuaDinh, ToanQuan, LiDinis, Maria Alzira PimentaQuan, XingwenDinis, RuiQuang Trung, TranDinulovic, DraganQue, LongDionysopoulos, DimitriosQuéau, YvainDios, FedericoQueiruga-Dios, AraceliDiraco, GiovanniQueluz, Maria PaulaDittmeier, SebastianQuer, StefanoDixit, Chandra K.Quero, GiuseppeDixon, SteveQuero, José ManuelDixon, TimothyQuerol, JorgeDiykh, MohammedQuevedo, Ivan R.Dizo, JanQuevedo, MiguelDjavadian, ShadiQuigley, AaronDJEBBARI, AbdelghaniQuijano, LauraDjenouri, YoucefQuinlan, JasonDjeziri, Mohand ArabQuiñones, BeatrizDjordjevic, Goran Lj.Quintana, MarcosDmitri, VoronineQuintas, JoãoDobbins, ChelseaQuiros, EliaDobinski, WojciechQuoc Viet, PhamDobos, RobinQuynh Hoa, LeDobosz, MarekR. Shamshiri, RedmondDobre, CiprianRa, In-hoDobre, OctaviaRabadán Borges, José ADobre, Robert-AlexandruRabah, HassanDobrea, Dan-MariusRabelo, RicardoDobrescu, LidiaRabiee, RamtinDobrev, YassenRabuffetti, MarcoDobrowolski, AndrzejRackauskas, SimasDobrzycki, ArkadiuszRackus, Darius G.Dochshanov, AldenRackwitz, FrankDocoslis, ArisRácz, AnitaDodds, ChrisRadac, Mircea-BogdanDogan, HakanRadadia, Adarsh D.Dogdu, ErdoganRadhakrishna, UjwalDoheny, Emer P.Radhakrishnan, SivaprakasamDolabdjian, ChristopheRadić, JoškoDolan, DanielRadicioni, FabioDolatabadi, ElhamRadoi, AnamariaDolenec, RokRadosinski, LukaszDolezel, PetrRadovic, MatijaDolgikh, Grigory IvanovichRadu, AleksandarDöllner, JürgenRadu, Gabriel LucianDolman, Bronwyn K.Raducanu, Bogdan C.Domaszewicz, JarosławRafique, WaqasDombek, GrzegorzRafsanjani, HamedDomingues, Maria FátimaRagaglia, MatteoDomingues, PatricioRaghavan, AswinDominguez, ManuelRahaim, MichaelDominguez, Rocio B.Rahimpour, AlirezaDominguez, SergioRahman, AshaqurDomínguez-Morales, Manuel JesusRahman, AshfaqurDomino, Krzysztof A.Rahman, HafizurDonadello, SimoneRahman, MoshiurDonaldson, NickRahman, MuhibDonati, ElisaRahman, MuhibUrDonati, MassimilianoRahman, MusfiqDonati, SilvanoRahmati, OmidDondrup, ChristianRahmatian, ArashDonelli, MassimoRaimondi, ValentinaDong, DuRainieri, CarloDong, ErbaoRaissouni, NaoufalDong, HaiRajabali, MustafaDong, HaifengRajan, GinuDong, JunliangRajapandiyan, PanneerselvamDong, LiRajaraman, SivaramakrishnanDong, LongjunRajasekaran, VijaykumarDong, QiuchenRajbhandari, SujanDong, ShaochunRajchowski, PiotrDong, ShurongRajeev, PatDong, Suh-YeonRajmic, PavelDong, TaifengRakoczy, AnnaDong, WenRakotondrabe, MickyDong, XichaoRaksincharoensak, PongsathornDong, YingRakun, JurijDong, ZhuxinRamalingame, RajarajanDong, ZiqianRamamurthy, ChandarasekaranDonlagic, DenisRamanaviciene, AlmiraDonlagić, DenisRamanavicius, ArunasDonnelly, RyanRamapriyan, Hampapuram K.Donskoy, IgorRamaswamy, ArunselvanDorafshan, SattarRamella, GiulianaDorazco-González, AlejandroRamezani, RaminDoria, AlbertoRamil, CarloD'Oro, SalvatoreRamirez Miquet, EvelioDoronzo, Domenico M.Ramirez, Juan ManuelDoroz, RafalRamirez-Gonzalez, GustavoDorri, AliRamirez-Paredes, Juan-PabloDos Reis, Catarina Isabel Ferreira Viveiros TavaresRamos Cózar, JuliánDos Santos, Eduardo NunesRamos, AntónioDos Santos, Filipe NevesRamos, VictorDosen, StrahinjaRamos-Ortiz, GabrielDospinescu, NicoletaRampa, VittorioDossi, CarloRamser, KerstinDou, ShanRamsköld, DanielDouik, AhmedRan, GuangzhaoDoulamis, AnastasiosRan, QiongDoulamis, NikolaosRana Muhammad Asad, KhanDoulos, Lambros T.Rana, DipakDovì, VincenzoRana, MdDovis, FabioRana, Soumya PrakashDow, DouglasRangel Magdaleno, José De JesúsDowling, Thomas P.F.Rangel, PabloDownes, JonRaniszewski, GrzegorzDowney, AustinRanjan, AlokDowrick, ThomasRanjan, RajeevDragojević, TanjaRanjan, RishabhDragomir, AndreiRanjkesh, AmidDragomir, FlorinRantalainen, TimoDrap, PierreRantos, KonstantinosDremin, ViktorRao, AshwinDresp-Langley, BirgittaRaoof, KosaiDrewniak, SabinaRaparelli, TerenzianoDrewnowski, JakubRapinski, P. JacekDrexler, PetrRaposo, CarolinaDreżewski, RafałRaposo, DuarteDrigas, Athanasios S.Rapp, MichaelDrira, HassenRaptis, TheofanisDrissi-Habti, MonssefRascon, CalebDroguett, Enrique LópezRashedi, EhsanDronova, IrynaRasheed, TahirDrossos, KonstantinosRashetnia, RezaDroz, ChristopheRashid, ZulqarnainDrusa, MarianRashkovska, AleksandraDu, DaweiRashwan, HatemDu, E(Sarah)Rasmussen, JesperDu, GuanglongRasmussen, MichelleDu, Jiang BingRaspopoulos, MariosDu, JianhaoRasskazov, IliaDu, JinyangRassõlkin, AntonDu, Li-DongRastegari, ElhamDu, QifeiRasti, PejmanDu, QingheRastogi, AnshuDu, ShuangRatassepp, MadisDu, XinRathod, VivekDu, XinliRatner, AlbertDu, YegangRattani, AjitaDu, Yi-ChunRatzke, MarkusDu, YongRaul, Sanchez-ReilloDu, ZhenhuiRaúl, SuárezDua, VivekRavankar, AbhijeetDuan, BenchunRavankar, AnkitDuan, FengRavi, RadhikaDuan, GuotaoRavikumar, NishantDuan, Huan-FengRavindra (Ravi), Nuggehalli M.Duan, LianRawassizadeh, RezaDuan, QiangRaz, AliDuan, WeiliRazaque, AbdulDuan, XiyuRazi, AbolfazlDuan, XuechaoRe, RebeccaDubbini, MarcoReale, FerdinandoDubey, HarishchandraReale, Michael JDubey, RameshwarReali, GianlucaDubey, RichaRealpe, MiguelDubey, VenkyRebelatto, João LuizDubreil, LaurenceRebordão, José ManuelDubuis, GuyReboud, JulienDucharne, BenjaminRecord, PaulDuchateau, NicolasRedding, BrandonDuchesne, MarcReddy Chittamuru, Sai VineelDucournau, GuillaumeRedon, NathalieDuda, SławomirRedweik, PaulaDudczyk, JanuszReed, William R.Dudescu, Mircea CristianReese, HeatherDudley, SandraRefice, AlbertoDudojc, BoleslawReggiani, LucaDudycz, HelenaRegnard, Jean-LucDuffy, JamesRego, GasparDugonjić Jovančević, SanjaRego, PaulaDuguleană, MihaiRegoutz, AnnaDuke, Daniel J.Rehman, Saeed UrDulińska, Joanna MariaReich, ChristophD'Ulizia, AriannaReich, SiegfriedDuma, LuminitaReichardt, ChristianDuma, Virgil-FlorinReichert, ChristophDumas, BrunoReid, Russell C.Dumciene, AudroneReiff-Marganiec, StephanDumitriu, MădălinaReig, CàndidDumoulin, CédricReimer, UlrichDunai, LarisaReina, Daniel GutiérrezDunea, DanielReinoso, OscarDung, Le TheReis, ArsénioDunn, JessilynReis, Manuel J.C.S.Dunn, MarkReiser, DavidDuong, Chi NhanReiser, RaoulDuplaga, MariuszReising, Donald R.Duplančić Leder, TeaReisis, DionysiosDupre, MatthieuReiter, AustinDupuis, PascalRejer, IzabelaDuque Méndez, Néstor DaríoRekas, MieczyslawDuque, OscarReljin, NatasaDuquennoy, MarcRemke, AnneDuraibabu, Dinesh BabuRen, BaiyangDurán Acevedo, Cristhian ManuelRen, GuanghuiDurand, SylvainRen, GuobinDurbin, Thomas D.Ren, Hai PengĎuriš, StanislavRen, HongliangDuro Carralero, NatividadRen, MingDuro, RichardRen, MingrongDurón, Sergio M.Ren, ShangjieDurrani, SalmanRen, Shun-qingD'Urso, GuidoRen, WeiDutoit, BertrandRen, WenqiDutta, AyanRen, XimingDutta, GaurabRen, YiDutton, NealeRen, YukunDyke, Shirley J.Ren, ZhileDylan, KobsarREN, ZhiyuanDymińska, LucynaRenard, FlorentDzantiev, B. B.Renard, Jean-BaptisteDziadak, BogdanRenault, EricDziong, ZbigniewRenevey, PhilippeDziri, AzizReni, SaumyaDziuban, JanRenna, FrancescoDziuda, ŁukaszRenner, Bernd-ChristianDziurdzia, PiotrRennie, AllanE Alahi, Md EshratRente, BrunoE. De Grande, RobsonRenzaglia, AlessandroEasley, ReginaRenzoni, FerruccioEastman, Roger D.Repa, Kristen StojakEbert-Uphoff, ImmeRerucha, SimonEbrahimi, AmirReschke, PeterEbrahimkhanlou, ArvinRescio, GabrieleEbrahimzadeh, AminRevel, Gian MarcoEbralidze, Iraklii I.Reverter, FerranEccleston, KimberleyRey, GaëtanEdo, Maria BermudezReyes Vera, ErickEdwards, AlasdairReyes, Bersain A.Edwards, GeoffreyReynolds, MarkEdwards, KeithRezaee, MohammadEdwards, VinceRezaei, MahdiEersels, KasperRezapour, MahdiEfremenko, DmitryRezazadegan, DanaEfthimiou, EleniRezk, AmgadEftimov, TomeRHEE, CHAE EUNEgarievwe, Stephen U.Rhee, HuinamEgea-Roca, DanielRhee, Jong IlEguchi, KeiRhee, WoogeunEhsani, HosseinRho, Hoon SukEibert, ThomasRhodin, HelgeEibl, GüntherRhudy, MatthewEid, NabilRi, ShienEilertsen, GretheRibas, Salvador J.Eisank, ClemensRibeiro Costa, Nuno AlexandreEisencraft, MarcioRibeiro Pinto, JoãoEitzinger, ChristianRibeiro, Bruno TeixeiraEiza, Mahmoud HashemRibeiro, HelenaEjaz, WaleedRibeiro, Jean-MarcEklund, AndersRibeiro, JoséEkonomou, LambrosRibeiro, Luís FrölénEkpo, SundayRicci, StefanoEkstrom, MikaelRicciardella, FilibertoEl ASSAF, AhmadRicciardi, ArmandoElafandy, RamiRicciardi, GiuseppeElahi, HassanRicciuti, ManolaElaissari, AbdelhamidRichard, NoëlElbaz, JohannRichelli, AnnaElble, Rodger J.Richiedei, DarioElbouchikhi, ElhoussinRichtera, LukasElbuken, CaglarRichtera, LukášEldefrawy, MohamedRida, ImadEler, Danilo MedeirosRidolfi, ElenaEleuch, HichemRiedel, TillElfergani, IssaRieger, RobertElfring, JosRies, MichalEl-Gendy, Ahmed A.Rifà-Pous, HelenaElgered, GunnarRighi, MarcoElhabian, Shireen Y.Righi, RodrigoElhajjar, RaniRigo, TomeuElias, PanagiotisRiguidel, MichelElings, AnneRijal, SantoshEliopoulos, Panagiotis A.Rijo, PatríciaElisa, KallioniemiRim, MinjoongElMasry, GamalRim, You SeongEl-mezyani, TouriaRimeika, RomualdasElmogy, MohammedRiminesi, CristianoEl-Osery, Aly I.Rinaldi, StefanoElrefai, Ahmed LotfyRincon, Jaime A.El-Sankary, KamalRingler, AdamEL-Sappagh, ShakerRinkevich, AnatolyElsayed, MohannadRiordan, JamesEl-Shafie, AhmedRiordon, JasonElshaw, MarkRios-Figueroa, Homero V.Elsts, AtisRioual, S.Elvidge, Christopher D.Ripardo De Alexandria, AuzuirElvira Segura, LuisRipka, PavelEl-Zubir, OsamaRipon, ShamimEmadi, ArezooRishpon, JudithEmanuele, TavantiRisojevic, VladimirEmeršič, ŽigaRissolo, DominiqueEminoglu, BurakRistic-Durrant, DanijelaEmmer, AdamRitrovato, PierluigiEmmert-Streib, FrankRitz, ChristianEnami, YasufumiRiul, AntonioEnder, FerencRivadeneyra, AlmudenaEndo, HiroyukiRivas, Carol A.Endo, PatriciaRivenson, YairEngel, JonathanRivera, DiegoEngelbrecht, RainerRivera-Armenta, José LuisEnguita, José MaríaRivero, Cristian RodriguezEnikov, Eniko T.Rivero-Angeles, Mario EduardoEnright, BernardRivolta, Massimo WalterEnríquez, J.G.Rizal, ConradEnser, HerbertRizwan, MuhammadEnsworth, JoshuaRizzardi, AlessandraEom, Joo BeomRizzi, MariaEom, KilhoRizzo, John-RossEon, RehmanRizzolo, SerenaEpifani, Dr. MauroR-Moreno, María D.Eramo, VincenzoRo, Jong-SukErdem, ArzumRobbins, KayErden, FatihRobert, Brzoza-WochErdogmus, EceRobert, JérémyEremin, ArtemRobert, TenzerErenas, Miguel MaríaRobert, ThomasEriksen, René LyngeRoberto, Sergio RuffoEriksson, MatsRobichaud, AlainErkaya, SelçukRobinson, MartinErmini, Maria LauraRobles, GuillermoErmoshkin, Alexey V.Robles, RamiroErol-Kantarci, MelikeRobles-Gómez, AntonioErra, UgoRoca, JosepErro Betrán, María JoséRocca, FabioErtman, SławomirRocca, PaoloEscalona, OmarRocha, Ana MariaEscamilla-Ambrosio, Ponciano JorgeRocha, AtslandsEscobar-Jiménez, RicardoRocha, JorgeEscobedo, CarlosRodehorst, VolkerEscobedo, PabloRodič, BlažEscorcia Carranza, IvonneRodrigo Mor, ArmandoEscudero, Carlos J.Rodrigo-Comino, JesusEskofier, BjoernRodrigues, AlexandreEsmeryan, KarekinRodrigues, AntonioEspinosa Zapata, FelipeRodrigues, JoelEspinosa, Hugo G.Rodrigues, José MiguelEspinosa, JuliánRodríguez Baena, LuisEspinoza, Carlos ZúñigaRodríguez Bolívar, Manuel PedroEspirito-Santo, AntónioRodriguez Diaz, Camilo ArturoEsposito, ChristianRodríguez Díaz, FranciscoEsposito, FlavioRodríguez Pérez, MiguelEsposito, GiuseppeRodriguez, AlejandroEsrafilzadeh, DornaRodríguez, AlfonsoEstay, HumbertoRodriguez, AngelEstêvão, João M.C.Rodriguez, J. Apolinar MuñozEsteves Da Silva, Joaquim C.G.Rodriguez, Juan ManuelEstrada-López, Johan J.Rodriguez, JuvenalEstrela, Vania VieiraRodriguez, ManuelEstudillo-Ayala, Julian M.Rodríguez, Manuel DíazEtien, ErikRodriguez, NancyEtoh, Takeharu GojiRodriguez, NibaldoEubank, Jarrod F.Rodríguez, Orlando CamargoEudes, AlexandreRodríguez, PatriciaEugenio, FazioRodriguez, SergioEvans, MatthewRodríguez-Delgado, MelissaEvans, NathanielRodríguez-Gonzálvez, PabloEven-Tzur, GiladRodriguez-Hernandez, Miguel A.Evtugyn, GennadyRodriguez-Lorenzo, LauraEwertowski, MarekRodriguez-Mendez, Maria LuzExertier, PierreRodriguez-Navas, GuillermoExpert, FabienRodriguez-Perez, DanielEyer, KlausRodriguez-Pino, MarcosEykyn, ThomasRodriguez-Pulido, Francisco J.Fu, Roger R.Rodriguez-Resendiz, JuvenalFabbiano, LauraRodriguez-Sanchez, CristinaFabelo, HimarRodruiguez, Luiz Carlos EstravizFabian, MatthiasRodway, JamesFabian, RalfRoh, HeejunFabianowski, WojciechRoh, YongraeFaccini, FrancescoRohac, JanFaccio, GretaRöhrig, ChristofFadda, MauroRoig, Ignacio BoschFadlallah, YasserRojas, CamiloFaes, LucaRojas, ElisaFagadar-Cosma, EugeniaRojhani, NedaFahad, HossainRojo-Álvarez, José LuisFahmy, YasmineRoldán Gómez, Juan JesúsFahn, Chin-ShyurngRoldán Pérez, JavierFaia, PedroRoldán-Blay, CarlosFaia, RicardoRomain, OlivierFain, BrunoRomanchik Kriuchkova, EugenioFaisal Haider, MohammadRomano, GiovanniFaisal, Shaikh NayeemRomanowski, AndrzejFajardo, MarioRomdhani, ImedFalade, Oluyemi PeterRomeo, LucaFalchi, FabrizioRomeo, StefaniaFalco, GianlucaRomero, Fernando CastañoFalk, ThorstenRomero-Cano, VictorFaller, Lisa-MarieRomero-Sanchez, MartinFallows, RichardRomero-Sanchez, Martin EnriqueFallucchi, FrancescaRomine, WilliamFamaey, JeroenRomolo, Francesco SaverioFamiano, MichaelRondanelli, RobertoFan, BinRonen, ShukiFan, Chih-PengRong, YuFan, DongruiRonga, DomenicoFan, FengleiRonningen, TJFan, HongqiRonnow, DanielFan, JinlongRonzani, DanieleFan, KaiRoose, PhilippeFan, KangqiRopelewska, EwaFan, Kuang-ChaoRopero, JorgeFan, LeiRoriz, PauloFan, PingyiRosa Da Silva, RobsonFan, ShuangshuangRosa, Bruno M.G.Fan, WenhuiRosado Muñoz, AlfredoFan, XingwangRosales, Alberto J.Fan, Yu-ChengRosario, DenisFan, ZhaoyanRosário, DenisFan, ZhengRosas, FernandoFandango, ArmandoRosati Papini, Gastone PietroFanfani, MarcoRosati, SamantaFang, ChengRösch, PetraFang, Da-GangRoscher, RibanaFang, HongliangRoselli, IvanFang, HuiRosen, CarlFang, JianRosenberg, LukeFang, PengRosenberger, ChristopheFang, ShiboRosendo, AndreFang, Shih-HauRosenstein, JacobFang, XudongRosenthal, AmirFang, Yi (China)Rosi, AscanioFang, Yi (USA)Rosina, ElisabettaFang, YihaiRosinová, DanicaFang, YinfengRos-Lis, JoseFang, ZhixiangRosner, DanielFanghella, PietroRosolem, Joao BatistaFanjul Bolado, PabloRossella, FrancescoFanjul-Bolado, PabloRossi, LucileFanti, AlessandroRossi, René M.Fapojuwo, AbrahamRossi, StefanoFara, LaurentiuRossignol, JeromeFarago, PaulRosso, CarloFarahani, BehzadRosso, ValeriaFarahani, Behzad VasheghaniRossomando, FranciscoFaramondi, LucaRostovtsev, YuriFareed, NadeemRosu, Stefan GeorgeFaria, Brígida MónicaRotariu, CristianFarlik, JanRotaru, MihaiFarooq, AamirRotella, NicholasFarroni, FlavioRoth, Bernhard WilhelmFaruolo, MariapiaRoth, ZviFasano, AndreaRothkrantz, LeonFasel, BenediktRoths, JohannesFateixa, Sara Isabel AugustoRothwell, Edward J.Faupel, FranzRotter, PawelFaust, OliverRotunno Filho, Otto CorrêaFaustino, AnaRouco, JoséFavaro, MarcoRouillard, JoséFawole, Olutosin CRoupioz, YoannFay, AlexanderRousakis, TheodorosFazenda, BrunoRoussey, CatherineFazzolari, RoccoRout, BhimsenFedder, Gary K.Rouzbahani, Hamid KarimiFedele, RosarioRoveri, MarcoFedeli, AlessandroRovini, ErikaFederic, MichardRovira-Garcia, AdriàFedorko, GabrielRoy, AbhishekFedorov, AlexanderRoy, DhruvajyotiFedorov, Fedor S.Roy, Jean-SébastienFedotov, DmitriiRoy, MohendraFei, LiguoRoy, SayanFei, TaiRoy, SpandanFei, YiyanRoyston, ThomasFei, ZesongRóżyło, KrzysztofFeichtner, ThorstenRuan, JunhuFeiertag, GregorRuano, Juan ChiachíoFekete, ZoltánRuat, MarieFelber, WolfgangRuban, DmitryFeld, SebastianRubins, UldisFelici-Castell, SantiagoRubio Loyola, JavierFelis Enguix, IvanRubio, HiginioFelis, IvanRubio, Jose De JesusFelisberto, PauloRuby, RukhsanaFelix, Anderson AndréRucco, RosariaFeliziani, MauroRucka, MagdalenaFeller, Jean-FrancoisRudnitskaya, AlisaFelski, AndrzejRueda-Roa, DignaFelt, WyattRuelas, AdolfoFemminella, MauroRuffin, PaulFeng, ChuangRügamer, AlexanderFeng, DongmingRühmer, DennisFeng, Guang-LiangRui, KunFeng, HuichengRuiperez-Valiente, José A.Feng, JianRuiz Armenteros, Antonio MiguelFeng, LangRuiz Zamarreño, CarlosFeng, PhilipRuiz, CarlosFeng, QiboRuiz, Vicente GonzálezFeng, QingshanRuiz-Díez, VíctorFeng, RuyiRuiz-Olaya, Andres F.Feng, YaokaiRullo, AntoninoFeng, YaozeRumyantseva, MarinaFeng, YihengRundo, FrancescoFeng, YuhongRundo, LeonardoFeng, YunfeiRunnerstrom, EvanFengchun, TianRunte, ChristophFeofilov, ArtemRuotsalainen, LauraFereidouni, FarzadRusinek, Cory A.Ferentinos, DinosRusinek, RobertFerhan, Abdul RahimRussell, BrandonFerin, GuillaumeRusso, MariateresaFerlin-Oliveira, SimoneRusso, MladenFernandes, FábioRustioni, LauraFernandes, MarceloRusu, CorneliuFernandes, StevenRusu, CristinaFernandes, TelmoRutkowski, LucileFernández Arguedas, VirginiaRuz Ruiz, Mario LuisFernández Fernández, JesúsRuz, Jose JaimeFernandez- Lopez, AntonioRužić, IgorFernandez Lozano, JesusRyan, EdmondFernandez Oro, Jesus ManuelRybak, JarosławFernández Ramos, María DoloresRybakowska, IwonaFernández, Evelio Martín GarcíaRybarczyk, YvesFernández, Óscar BelmonteRydosz, ArturFernandez, RoemiRyndin, Eugeny A.Fernández, SuselRyoo, IntaeFernández, TomásRyoo, Young-JaeFernández-Caramés, Tiago M.Ryu, HyejeongFernandez-Carmona, ManuelRyu, JongbinFernandez-Garcia, RaulRyu, Ju SeokFernandez-Granero, Miguel AngelRyzhii, Maxim V.Fernández-Lozano, JavierRzepecka, ZofiaFernández-Montes, AlejandroRzhanov, YuriFernandez-Prieto, ArmandoRzucidlo, PawelFernández-Romero, Juan ManuelRzymoeski, MateuszFernandez-Veiga, ManuelSaad, WalidFernández-Vilas, AnaSaadane, RachidFernando, NiroshinieSaadaoui, MohamedFerracuti, FrancescoSABBAN, ALBERTFerrández-Pastor, Francisco JavierSaberioon, MohammadmehdiFerrandis, Jean-YvesSabri, YliasFerrando, IlariaSabsabi, MohamadFerrando-Villalba, PabloSaccomandi, PaolaFerrante, CamillaSachs, JürgenFerrara, Maria AntoniettaSadati, S.M.HadiFerrari, ClaudioSadeghi Esfahlani, ShabnamFerrari, PaoloSadeghian, Ramin BananFerrari, SilviaSadeghi-Tehran, PouriaFerrario, Maria FrancescaSadhu, AyanFerraris, ClaudiaSadjadi, FiroozFerraro, AngeloSadok, Djamel F. HadjFerrein, AlexanderSadowski, ŁukaszFerreira, AnaSadre, RaminFerreira, André G.Sadreazami, HamidRezaFerreira, Artur J.Saeed, AdilFerreira, BrunoSaeed, KhalidFerreira, Daniel BassoSaeed, NasirFerreira, JoaoSaeedi, RamyarFerreira, LucasSaeedifar, MiladFerreira, Marta S.Saez De Adana, FranciscoFerreira, Nuno M. F.Saez, YagoFerreira, Paulo Victor RodriguesSafdar, Ghazanfar AliFerrer, BelenSaffell, JohnFerrer, GonzaloSaffih, FaycalFerrero Martín, Francisco J.Safian, RezaFerretti, LucaSafonov, AlexanderFerri, GiuseppeSaga, SatoshiFerruz, JoaquinSaggiomo, VittorioFerus, MartinSagheer, AlaaFeteira, AntonioSagiya, TakashiFiala, PavelSaha, Jhantu KumarFialho, LuisSaharan, LalitaFialho, MarcioSahin, Furkan EFiandrino, ClaudioSaho, KenshiFiandrotti, Attilio (France)Sahore, VishalFiandrotti, Attilio (Italy)Sahu, JagajjitFidalgo, EduardoSahu, SushantFidan, BarisSahuguede, StephanieFidanova, StefkaSaia, RobertoFidge, ColinSaijo, YoshifumiFiebig, Uwe-CarstenSailhan, FrancoiseFiedler, GoeranSaini, RajkumarFigiel, AdamSainz De Abajo, BeatrizFiglus, TomaszSainz, NekaneFigueira, Rita BacelarSajjanhar, AtulFigueiredo, LinoSakai, KenjiFigueras, EduardSakai, TakeyasuFigueroa Lorenzo, SantiagoSakaino, ShoFigurski, MariuszSakamoto, João Marcos SalviFihey, Jean-LucSakellariou, MichaelFilchev, LachezarSakkalis, VangelisFilin, SagiSaku, TakashiFilip, PetrSakumura, YuichiFilipescu, AdrianSakuno, YujiFilipovic, LadoSalach, JacekFilippeschi, AlessandroSalagean, TudorFilippini, MauroSalah, KhaledFiljar, RenatoSalam, AdulFilos, Jasonsalama, Khaled NabilFindeiß, SvenSalamone, FrancescoFingas, MervSalanova Grau, Josep MariaFink, OlgaSalas, RodrigoFinkler, AmitSalata, StefanoFino, PeterSalazar, AddissonFiore, UgoSalazar, SergioFiorentino, CostanzaSalceanu, AlexandruFioretti, SandroSalceda-Delgado, GuillermoFiorillo, AntoninoSalcedo, Walter J.Fiorini, LauraSalcic, ZoranFirebaugh, SamaraSaldias, GonzaloFiroozabadi, RezaSaleh, TawfikFischer, AndreasSalehizadeh, HamedFischer, AndrewSalerno, Valerio MarioFischer, GeorgSalewski, MirkoFischer, IngaSalgueiro, José RamónFleischmann, FriedrichSalguero, Alberto G.Flemmer, ClaireSalice, FabioFletcher, KennethSalichs, MiguelFlieger, JolantaSalido, JesúsFlorea (Voicu), CarmenSalido-Monzú, DavidFlorea, AdinaSaligane, MehdiFlorea, CorneliuSalk, CarlFlorea, LauraSallam, Khaled AFlorent, NolotSalm, CoraFlores Tapia, DanielSalman, EmreFlores-Fuentes, WendySalmani, HassanFlorindo, João BatistaSalmeron-Sanchez, ManuelFloros, GeorgeSalski, BartłomiejFlowers, GeorgeSaltaren, RoqueFodor, MariettaSaltas, VassilisFoguel, Marcos ViniciusSalvador, GilFoi, MarcoSalvador, PabloFolea, SilviuSalvador, RenanFolke, MiaSalvati, DanieleFonod, RobertSalvatore, SurdoFonseca, AnaSalvatori, RosamariaFonseca, JoséSalvetti, OvidioFontgalland, GlaucoSalvo, PietroFoong, ShaohuiSam, LydiaFøre, MartinSama, MichaelForehead, HughSamadian, KavehFormaggio, EmanuelaSamama, NelFornaini, CarloSamanta, Pralok KumarFornaro, ClaudioSamanthula, Bharath KumarForner-Cordero, ArturoSamaras, NicholasFörster, AnnaSamardžić, NatašaFort, AdaSamat, AlimFortes, SergioSamavedham, LakshminarayananFortuna, LuigiSamczyński, PiotrFortunati, StefanoSamek, OtaFosalau, CristianSamiappan, SathishkumarFosson, SophieSamie, FarzadFoster, ChristopherSampalli, SrinivasFoster, JamesSampaolo, AngeloFotia, LidiaSampathkumar, ArunFotiadis, DimitriosSampietro, DanieleFotiou, NikosSample, DavidFotouhi, AzadeSamtani, SagarFotouhi, HosseinSan, HaishengFoudeh, AmirSánchez Montero, DavidFourati, HassenSanchez, AlejandroFourati, NajlaSánchez, Borja BordelFournier, GeorgesSánchez-Cambronero, SantosFowler, MichaelSanchez-Iborra, RamonFox, Neil I.Sanchez-Lopez, Jose LuisFőző, LadislavSanchez-Lopez, Juan De DiosFraboni, BeatriceSánchez-Molina, Jorge AntonioFraccaro, PaoloSánchez-Rodríguez, DavidFraga, Francisco J.Sanchez-Rojas, Jose LuisFraga, Mariana AmorimSánchez-Somolinos, CarlosFragou, OlgaSánchez-Soto, Luis L.Fraile Marinero, Juan CarlosSancho Bru, Joaquín LuisFrancese, RitaSancho-Gómez, José LuisFrancés-Soriano, LauraSanda, SureshFrancik, SławomirSandner, ThiloFrancisco Jose, Torcal MillaSandoval Goes, Luiz CarlosFranco, Diego LeoniSandoz, PatrickFranco, LeonardoSanduleac, MihaiFrangi, AttilioSandulescu, RobertFranke, JörgSandwall, PeterFrappart, FrédéricSang, JunFrasca, FrancescaSang, MeiFrassetto, FabioSang, Tzu-HsienFraternali, FrancescoSangeux, MorganFratini, AntonioSankowski, DominikFratu, OctavianSanmartin, PaulFrazão, LuísSan-Miguel, AdrianaFredianelli, LucaSanna, AndreaFreedman, KevinSan-Segundo, RubenFreiherr Von Lukas, UweSan-Segundo, RubénFreire, André PimentaSantamaria, Amilcare FrancescoFreire, Roberto ZanettiSantamaría-Peña, JacintoFreitas, Anderson ZanardiSantana, JoseFreitas, Edison Pignaton DeSanthanam, KSVFrense, DieterSanthi, B.Frese, UdoSanti, FabrizioFrew, EricSantiago-Aviles, Jorge J.Freymueller, JeffSantiago-Rodríguez, EfraínFrias, Octavio GutierrezSantin, Altair OlivoFrick, ManfredSantiranjan, ShannigrahiFrick, VincentSantorelli, AdamFricke, HartmutSantoro, CarmenFriederich, FabianSantoro, CorradoFriedrich, BernhardSantoro, EugenioFriedrich, Christoph M.Santoro, FrancescoFriedrichs, GernotSantoro, MattiaFriedt, Jean MichelSantos, AbelFriedt, Jean-MichelSantos, Auteliano AFriesen, MarenSantos, Daniel Rodrigues DosFrigo, GuglielmoSantos, Jesús DanielFRIGURA-ILIASA, Flaviu MihaiSantos, José PedroFrincu, MarcSantos, Juan M.Frindel, CaroleSantos, PauloFrisch, BenjaminSantos, RicardoFrizera-Neto, AnselmoSantos, RodrigoFrnda, JaroslavSantos-García, GustavoFröhlich, Antônio AugustoSäntti, TeroFromenteze, ThomasSanwar Hosen, A. S. M.Frontoni, EmanueleSanz-Pascual, María TeresaFroriep, Ulrich P.Sapena-Baño, AngelFrovel, MalteSapienza, MichaelFrüh, SusannaSara, RadimFrustaci, FabioSarac, MineFryskowska, AnnaSarangapani, JagannathanFryśkowska, AnnaSardi, Giovanni M.Fu, ChunyunSardini, EmilioFu, HailingSareen, HarpreetFu, HaiqiangSargano, Allah BuxFu, HongyanSarghini, FabrizioFu, JianyuSargolzaei, ArmanFu, JihuaSarigiannidis, PanagiotisFu, LiSarjas, AndrejFu, LongshengSarkar, SoumicFu, PengSarraguça, MafaldaFu, ShuSarría, Francisco AlonsoFu, XiaoSarwar Rana, Abu ul HassanFu, XiaomeiSarwar, YusufFu, XinwenSasagawa, KiyotakaFu, YuSasaki, KenFu, YuchengSasayama, TeruyoshiFu, YunqiSassa, ShinjiFu, ZhihongSastry, Shankar P.Fuchs-Kittowski, FrankSathyachandran, Sanath KumarFudickar, SebastianSatici, AykutFuentes, RamónSato, RyutaFuentes, SigfredoSato, ToshihisaFuentes-Fernández, RubénSatori, ShinFuentes-Pineda, GibranSatorres, Silvia MartínezFujdiak, RadekSatrapinskyy, LeonidFujii, MasahikoSauermann, StefanFUJII, SatoshiSaukh, OlgaFujimura, AtsushiSaura, José RamónFujinami, KaoriSavaglio, ClaudioFujiwara, EricSavastano, MatteoFujiwara, KoheiSavazzi, PietroFujiwara, KoichiSavazzi, StefanoFukuda, TakeshiSaveliev, AnatolyFukushima, NorishigeSavi, PatriziaFunatsu, EiichiSavic, StevanFurfaro, FilippoSavin, IgorFurferi, RoccoSavino, RaffaeleFurnari, AntoninoSavoia, Alessandro StuartFurtak, JanuszSavvaidis, ParaskevasFurusawa, HiroyukiSawacha, ZimiFurutani, RyoshuSawada, TadamasaFusaglia, MatteoSawicki, DariuszFusco, FrancescoSawicki, PiotrFusco, GiovanniSayed-Ahmed, Ezzeldin YazeedFuse, TakashiSazhin, MikhailFusiek, GrzegorzSberveglieri, VeronicaFuss, Franz KonstantinScalenghe, RiccardoFuster-Guilló, AndrésScalera, LorenzoFutagawa, MasatoScalise, LorenzoFuzzo, DanielaScandurra, AntoninoG Menon, VarunScannapieco, AntonioG. Anagnostopoulos, GrigoriosScano, AlessandroGaber, JaafarScappaticci, LorenzoGabr, MoustafaScaradozi, DavidGabriel-Alexandru, ConstantinScaradozzi, DavidGabrielli, AlessandroScaramuzza, MatteoGachagan, AnthonyScarpato, NoemiGaddam, AnuroopScarpiniti, MicheleGadjanski, IvanaScarselli, GennaroGadsden, S. AndrewSchafrik, Steven J.Gaetani, IsabellaSchall, Mark C.Gaft, MichaelSchartner, ErikGaggi, OmbrettaSchasfoort, RichardGagneja, KanwalinderjitSchauer, Caroline L.Gai, KekeSchauer, ThomasGai, ShaoyanSchazmann, BenjaminGaillot, Davy P.Scheeren, EduardoGaitan, Vasile GheorghitaScheers, BartGaitas, AngeloScheicher, Ralph H.Gajda, JanuszScheller, Frieder W.Gajic, DragoljubSchenato, LucaGalanis, NikolaosSchepers, JamesGalante, FrancescoSchetinin, VitalyGalan-Vidal, Carlos AndresSchiano Lo Moriello, RosarioGalas, RomanSchiavon, MicheleGalati, GaspareSchiefler, Nivaldo T.Galatus, RamonaSchilberg, DanielGaláž, ZoltánSchilling, MeinhardGaldames, PatricioSchillinger, BurkhardGalea, ChristianSchinazi, VictorGaleana Zapién, HiramSchirrmann, MichaelGalehdar, AmirSchlachetzki, JohannesGalembeck, FernandoSchlaepfer, DanielGalgalikar, RohanSchlenstedt, ChristianGaliatsatos, NikolaosSchlicke, HendrikGalić, ZdravkoSchlippert, DennisGalinetto, PietroSchmauss, BernhardGallacher, DavidSchmeink, AnkeGallagher, JohnSchmid, MaurizioGallego, AntolinoSchmid, Moritz S.Gallego-Elvira, BelenSchmidt, AdamGallo, MarianoSchmidt, JochenGallo, PierluigiSchmidt, MichaelGalo, MauricioSchmitt, CorinnaGalopin, NicolasSchmitt, KatrinGalstyan, VardanSchmitt, RobertGalvan-Tejada, Carlos EricSchmitz, AlexanderGalve, Joan MiquelSchmitz, RolandGálvez-García, GermánSchnakenberg, UweGamage, KelumSchneider, BertrandGambarova, Pietro G.Schneider, Fabio KurtGambi, EnnioSchneider, MartinGamboa, HugoSchneider, MichaelGamella Carballo, MariaSchneider, TizianGan, DianeSchniepp, RomanGan, FengSchoedel, AlexanderGandhi, VaibhavSchofield, JonathonGandolfi, StefanoScholkmann, FelixGanesan, BaskaranScholl, Philipp M.Ganesana, MallikarjunaraoScholten, KeeGang, TingtingSchubert, Anna-LenaGanim, ZiadSchuss, ChristianGanser, ChristianSchüßler, MartinGao, FeiSchwarz, BenediktGao, FengSchwarz, Ulrich TheodorGao, HongboSchwarzbach, PaulGao, HuiSchwertfeger, SörenGao, JingxiangSchwickert, Sportwiss. LarsGao, KaiSciarrone, AndreaGao, KeScionti, AlbertoGao, LianliScioscia, FlorianoGao, LianruScorza, AndreaGao, RuipengScotney, BryanGao, ShangceScribante, AndreaGao, ShaobingSeal, AyanGao, WeiSebastian, Jose M.Gao, XiangSebestyen, GheorgheGao, XiaofengSedky, MohamedGao, XiaomengSedláček, PetrGao, XiaomingSedlák, PetrGao, XinSedláková, VlastaGao, XuefeiSee, ChanGao, ZhenzhenSeel, ThomasGao, ZhiSeeling, PatrickGao, ZhiyuanSegawa, HiroyoGarau, AlessandraSegoni, SamueleGarces, HugoSegovia Vargas, DanielGarcia Gonçalves, Luiz MarcosSegovia-Fernandez, JeronimoGarcía Sánchez, ManuelSegreto, TizianaGarcía, Alicia CarriónSegura, JaumeGarcia, Angel PontinSeichepine, FlorentGarcía, AntonioSeidlitz, HolgerGarcía, CarmeloSeita, MatteoGarcía, Carmelo R.Seitz, JochenGarcia, CeciliaSeitz, W. RudolfGarcia, Diana VilelaSekretaryova, AlinaGarcia, EmiSelesnick, IvanGarcia, Jaume SeguraSellami, AkremGarcia, Jose Manuel CanoSelva, MassimoGarcía, Juan C.Selyanchyn, RomanGarcía, Marcelo V.Semancik, StephenGarcia, MissaelSemkov, KrumGarcía, TaniaSemmling, MaximilianGarcia, VictorSemprini, MariannaGarcia-Alegre, MariaSen Gupta, SouravGarcia-Alonso, JoséSen, DebarshiGarcia-Aracil, NicolasSen, SatyabrataGarcía-Cámara, BraulioSenger, MoritzGarcia-Ceja, EnriqueSengupta, DebarunGarcía-Cortés, SilverioŞenyürek, Volkan YusufGarcía-Cuesta, EstebanSeo, Byung-KukGarcía-Diego, Fernando J.Seo, Dong-HoanGarcía-Fernández, Ángel F.Seo, DongmahnGarcia-Font, VictorSeo, HogeonGarcia-Garcia, LeonardoSeo, JiwonGarcía-Herrero, JesúsSeo, JungmokGarcia-Lagos, FranciscoSeo, JunwonGarcía-Lamont, FaridSeo, Na JinGarcia-Leon, ManuelSeo, ToruGarcía-Magariño, IvánSeoane, FernandoGarcía-Massó, XavierSeok, SenhoGarcía-Mateos, GinésSeow, CheekiatGarcía-Miquel, HectorSepasgozar, SamadGarcía-Morales, VladimirSepman, AlexeyGarcia-Naya, Jose A.Sepulcre, MiguelGarcia-Palacios, EmilianoSepuru, Krishna MohanGarcía-Peñalvo, Francisco JoséSequeira Gonçalves, Paulo JorgeGarcia-Perez, ArturoSequeira, JeanGarcía-Pineda, MiguelSerackis, ArturasGarcía-Ramos, Francisco J.Serafin, PiotrGarcía-Rubio, CarlosSerafín, VeronicaGarcia-Saavedra, AndresSerafín, VerónicaGarcia-Sanchez, Antonio-JavierSerban, AdrianaGarcía-Sánchez, José RafaelSerikov, ArkadyGarcía-Varea, IsmaelSerodio, CarlosGarcía-Vázquez, Juan-PabloSerpelloni, MauroGardel-Vicente, AlfredoSerpi, AlessandroGarg, HarishSerrà, AlbertGarg, LalitSerradilla, FranciscoGargiulo, Gaetano D.Serrano, BereniceGarmatyuk, DmitriySerrano, João Manuel Pereira RamalhoGarnica, OscarSerrano-García, David IgnacioGarnier, VincentSerrano-Pertierra, EstherGaroli, DenisSerres, JulienGarrido, PiedadSerry, MohamedGarriga, RosaServati, AmirGarza, Horacio AhuettServin, ManuelGašinec, JurajSesay, AbuGąska, AdamSešek, AleksanderGaspar, SzilveszterSeshadri, PranayGašparović, MateoSeung-Hoon, HwangGasparri, RobertoSevic, DragutinGastal, Eduardo Simoes LopesSevil, Hakki ErhanGastaldi, LauraSeweryn, KarolGatesman, Andrew J.Seyedi, MirHojjatGatteschi, ValentinaSeymour, JohnGaur, GirijaSfarra, StefanoGautam, DeepakSferrazza, CarmeloGautam, RekhaSgobbi, Lívia FlorioGavazzi, BrunoSgora, AggelikiGaviña, PabloSha, ChaoGavrilova, MarinaSha, ZongyaoGawhary, O. ElShabbir, Syed-AbdulGawronek, PelagiaShafiee, MahmoodGazquez Parra, Jose AntonioShafiq, OmairGazzoni, MarcoShafique, MuhammadGbadebo, AdenowoShafiullah, GMGe, SamShah, AhmarGe, XiaohuShah, JainilGe, XinminShah, MilinkumarGe, XudongShah, PuravGe, YufengShah, Syed TariqGe, ZhiqiangShah, TejalGe, ZongYuanShah, VaibhavGebicki, JacekShahaboddin, ShamshirbandGębicki, JacekShahbazi, MozhdehGedikli, Ersegun DenizShahidi, RezaGeints, Yurii EShahpari, MortezaGeist, JonShahriari, YaldaGellini, CristinaShahrour, IsamGeman, OanaShakeel, HamzaGemba, Kay L.Shakev, NikolaGenders, WadeShakthivel, DhayalanGendron, PaulShamir, EylonGenest, MarcShamonin, MikhailGenet, HeleneShan, HangguanGenez, Thiago LopesShan, LinGeng, ChunhuaShan, MaoGeng, GuangchaoShanafield, MargaretGeng, JianghuiShang, FangGeng, KunShang, PengjianGeng, TaoShang, WeiweiGeng, WeidongShang, YilunGengheng, ZhouShanjani, YaserGennarelli, GianlucaShanmugam, BharanidharanGenovese, AngeloShannigrahi, SusmitGenovese, MarcoShao, DangdangGenovese, MariaShao, HaidongGenovesi, SimoneShao, HaoGentili, RodolpheShao, LeiGentner, ChristianShao, PengGeorgakopoulos, StavrosShao, ShihaiGeorganos, StefanosShao, WeimingGeorge, AnjithShao, XinxingGeorge, ChatzigeorgiouSharif-Khodaei, ZahraGeorge, DeepuSharma, LakeshGeorge, Thomas F.Sharma, MrigankGeorgiadis, CharalamposSharma, NitinGeorgieva, BilianaSharma, PradipGeorgieva, TsvetelinaSharma, Ram KrishanGeorgopoulos, AndreasSharma, SanjivGeorgousis, GeorgiosSharma, VishalGeradts, ZenoSharp, MaryGerasimaitė, RūtaSharpe, ShaunGerasimova-Meigal, Liudmila I.Shaw, HarryGerber, PaulShaw, KirstyGerber, PhilippeShe, BingGerbino, SalvatoreSheen, Horn-JiunnGerman-Sallo, ZoltanShehata, NaderGeruo, A.Sheidaei, AzadehGeza, HusiSheinker, ArieGföhler, MargitShekaramiz, MohammadGhafar-Zadeh, EbrahimShekarpour, SaeedehGhahramani, AliShell, JethroGhahremani, KasraShellaiah, MuthaiahGhaleb, BaraqShemer, AmirGhalichechian, NimaShemer, LevGhannoum, AbdulRahmanshen, chongGhareh Baghi, ArashShen, DanyuGhasemkhani, MohammadrezaShen, DazhongGhavidel, AliShen, HuanfengGhayoumi, MehdiShen, Hui-LiangGhayvat, HemantShen, JianGhemari, ZineShen, JianbingGheorghiu, Razvan AndreiShen, JunGherbi, AbdelouahedShen, LiGherlone, MarcoShen, MeiGhibaudo, GérardShen, QiangGhidoni, StefanoShen, QingGhilain, NicolasShen, QingmingGhisi, AldoShen, RuiqingGhodake, GajananShen, WenGholamalizadeh, EhsanShen, WenlongGhommam, JawharShen, YanfengGhoreyshi, Seyed MohammadShen, YaochunGhosal, SutapaShen, YonghangGhosh, ArijitShen, YuGhosh, AritraShen, YuanGhosh, ChiranjitShen, YuechengGhosh, DebanjanaShen, ZhigangGhosh, SohamSheng, JianxiongGhosh, SubrataSheng, JunGia, Tuan NguyenSheng, QiwenGiachero, AndreaSheng, WenyiGiachetti, AndreaSheng, ZhichaoGiacomini, RenatoShepelev, VladimirGialampoukidis, IliasShepherd, JonathanGialelis, JohnSherman, PaulGianella, MicheleSherratt, R. SimonGiannakeas, NikolaosSheta, BassemGiannakis, IraklisSheu, Ruey-KaiGiannakis, KonstantinosShevkunov, IgorGiannelli, CarloShi, ChengzhiGianniou, MichailShi, Hong-LingGiannoulis, SpiliosShi, JunboGiardina, GiorgiaShi, JunhuiGiarre, LauraShi, KeGibbins, David R.Shi, KunGibbons, Steven J.Shi, LeiGiberti, HermesShi, PengGibson, ChristopherShi, QiongfengGibson, DesmondShi, RonghuaGibson, LesleyShi, TielinGienko, GennadyShi, WenboGiergiel, MariuszShi, XiuzhiGierz, LukaszShi, YeyinGiggins, Oonagh M.Shi, YiweiGiglio, MarilenaShi, YunboGijón-Nogueron, GabrielShi, ZhiguoGil, Arturo AparicioShi, ZhiyongGil, DavidShibata, NaokiGil, GustavoShibayama, JunGil, PabloShibuya, KyukiGilaberte, Raquel LacuestaShieh, Chin-ShiuhGill, Stephen D.Shieh, Hsin-JangGill, Sukhpal SinghShieh, Wann-YunGillich, Gilbert-RainerShields IV, C. WyattGilly, KatjaShigeta, RyoGilmore, ColinShigeyasu, TetsuyaGimeno Blanes, Francisco JavierShih, Ching-longGini, FulvioShih, Mei-JuGini, GiuseppinaShihavuddin, ASMGinis, PieterShiki, Sidney BruceGinzburg, BorisShikida, MitsuhiroGinzburg, PavelShilov, NikolayGiofrè, Vincenzo PasqualeShim, Duk-SunGioia, CiroShim, JiwookGioli, BeniaminoShimabukuro, Milton HirokazuGiordano, Maria CaterinaShimabukuro, YosioGiorgetti, AndreaShimada, KunioGiorgi, GiadaShimizu, FlavioGiorgini, AntonioShimizu, Flavio MakotoGiosan, IonShimizu, YouichiGiosan, Ion-AugustinShin, Bo-SungGioux, SylvainShin, Chow-ShingGirelli, Valentina AlenaShin, DongjunGiri, ParitoshShin, HangsikGirolami, MicheleShin, JaeyoungGissot, SamuelShin, JitaeGiudice, NicholasShin, Jong-HoGiudice, OliverShirvaikar, MukulGiungato, PasqualeShirvani Moghaddam, ShahriarGiurgiutiu, VictorShiryayev, OlegGiusti, ElisaShiskin, SergeiGiusto, EdoardoShiu, Jau-YeGiwa, AdewaleShiu, Yi-ShiangGjevestad, Jon GlennShlyagin, MikhailGjoreski, HristijanShoaib, SultanGkoupidenis, PaschalisShoaran, MahsaGkritzalis, ThanosShokri-Ghadikolaei, HosseinGlas, DylanShokrolah Shirazi, MohammadGlavin, MartinShoombuatong, WatsharaGleich, DusanShorokhov, AlexanderGligor, AdrianShort, MichaelGlinšek, SebastjanShpacovitch, VictoriaGlisic, BrankoShrestha, Anish PrasadGlonek, GrzegorzShrestha, NamitaGlösekötter, PeterShrestha, RajuGłowacki, MichałShrestha, SudhirGlowacz, AdamShrirao, AnilGłowiński, SebastianShrivastava, AatmeshGnade, BruceShtenberg, GiorgiGnade, Bruce E.Shtrepi, LouenaGnyp, Martin L.Shu, LeiGobo, João Paulo AssisShu, LiangGockenbach, ErnstShu, LinGodina, RaduShuangguan, DonghuiGodoy, EduardoShue, SamGoes, JoãoShukla, Sudheesh K.Goga, NicolaeShults, RomanGogneau, NoelleShumyantseva, Victoria V.Goh, Shu TingShurpik, DmitriyGoicoechea, JavierSi, DongGoldoni, AndreaSi, MingsuGoleva, RossitzaSibilski, KrzysztofGollee, HenrikSiddiqui, Salman I.Gollmann, DieterSidike, PahedingGołofit, KrzysztofSieben, VincentGolston, LeviSieradzki, RafałGoltz, Douglas M.Sierociuk, DominikGoluch, Edgar D.Sierra, BasilioGomba, GiorgioSierra, Jesus EnriqueGomede, EvertonSierra-Hernandez, Juan MGomes, Anderson S. L.Sierra-Rosales, PaulinaGomes, DiogoSiewert, SamGomes, Guilherme FerreiraSignoroni, AlbertoGomes, Ruan DelgadoSigolaeva, Larisa V.Gomes, TeresaSihvola, AriGómez Aguilar, José FranciscoSiirtola, PekkaGomez Balderas, Jose ErnestoSikdar, ShirsenduGomez Deniz, LuisSikirzhytski, VitaliGómez Déniz, LuisSik-Lanyi, CeciliaGómez Fernández, Juan F.Sik-Lányi, CecíliaGómez Muñoz, Carlos QuiterioSikora, JanGómez Polo, CristinaSikorska, EwaGomez, CécileSikorski, WojciechGómez, LuisSila-Nowicka, KatarzynaGómez, MarisolSilina, YuliyaGómez-Aguilar, José FranciscoŠiljeg, AnteGomez-Barrero, MartaSilva, AdãoGómez-de-Gabriel, Jesús M.Silva, Agnelo R.Gómez-Espinosa, AlfonsoSilva, BhagyaGómez-Herrero, J.Silva, FilipeGómez-Ortiz, DavidSilva, Gabriel A.Gomez-Pau, AlvaroSilva, Hugo MiguelGómez-Pavón, Luz Del CarmenSilva, IvanovitchGomez-Ruiz, Jose AntonioSilva, LucianoGomez-Tornero, Jose LuisSilva, RGGomis Bresco, JordiSilva, Rone Ilídio DaGonçales, Vinicius R.Silva, Saimon MoraesGonçalves, Eder Mateus NunesSilva, SusanaGonçalves, GilSilva, WashingtonGonçalves, José AlbertoSilvera-Tawil, DavidGonçalves, PedroSilverio, VaniaGoncu-Berk, GozdeSilvestre, MiguelGonenc, BerkSim, Min KyuGong, Cihun-SiyongSim, Sung-HanGong, KuangSima, ChaotanGong, Nan-WeiSimani, SilvioGong, ShuSimas Filho, Eduardo F.Gong, SueSimeonov, VasilGong, WenlinSimion, CristianGong, WenyuSimion, Cristian E.Gong, ZijunSimonetta, FedericoGonizzi Barsanti, SaraSimonis, IngoGonzález De Santos, Luis MiguelSimonov, NikolaiGonzález Manzano, LorenaSimova, PetraGonzález Pérez, IsaíasSinapius, MichaelGonzalez Vila, AlvaroSing, Swee LeongGonzalez, AlfredoSingh, AdityaGonzález, Amador M.Singh, AmardeepGonzález, ApolinarSingh, AmitGonzalez, FernandoSingh, DhananjayGonzalez, IvanSingh, GautamGonzález, Luis CrovettoSingh, Kamal DeepGonzalez, RodrigoSingh, MadhusudanGonzález, Sara RodríguezSingh, RaghurajGonzález., Luis C.Singh, ShaktimanGonzález-Ayestarán, RafaelSingh, UdayGonzález-Briones, AlfonsoSingh, YogangGonzalez-Carrasco, IsraelSinha, RajuGonzález-Cortés, AraceliSinha, SunilGonzález-de-la-Rosa, Juan-JoséSinibaldi, AlbertoGonzalez-Landaeta, RafaelSinibaldi, EdoardoGonzález-López, AntonioSiniscalchi, Sabato MarcoGonzalez-Ortega, DavidSioma, AndrzejGonzález-Parada, EvaSipavičienė, SaulėGonzález-Sánchez, M. I.Šipoš, MartinGonzalez-Silveira, MartaSips, PatrickGonzalez-Silvera, AdrianaSiqueira Dias, José A.González-Teruel, Juan D.Sirmaçek, BerilGonzalvez, Pablo RodriguezSisinni, EmilianoGoodall, NoahSistla, SrinivasGoodchild, M. S.Sitányiová, DanaGoodin, ChristopherSitnik, RobertGoodin, DouglasSivasubramanian, KathyayiniGoodney, AndrewSiverns, JamesGooneratne, Chinthaka P.Skaggs, SheldonGoossen, KeithSkalský, MichalGopalakrishnan, KothandamSkarlatos, AnastassiosGopalan, SaianandSkarlatos, DimitriosGopalsami, NachappaSkeie, Nils-OlavGórak, RafałSkibicki, JacekGorawski, MichalSkic, AnnaGorcin, AliSkidanov, RomanGordon, TimothySkierucha, WojciechGórecki, TomaszSkilodimou, Hariklia D.Gorgoglione, AngelaSkinner, JackGorodkiewicz, EwaSkládal, PetrGorricho, Juan-LuisSklivanitis, GeorgiosGorski, ŁukaszSkočaj, DanijelGórski, MarcinSkočir, PavleGosalvez, JorgeSkordos, AlexGoshtasby, ArdeshirSkrickij, ViktorGostar, AmiraliSkrzypczyński, PiotrGosztolya, GáborSkwor, TroyGotchev, Atanas P.Slámová, MartinaGotor, Alicia AyerdiSlavič, JankoGotsis, KonstantinosSleewaegen, Jean-MarieGottardi, MassimoSlouka, ChristophGottwald, AlexanderSlusanschi, EmilGötzelmann, TimoSlyne, FrankGou, JianpingSmagowski, PiotrGoubej, MartinSmal, IhorGoud, K YugenderSmall, ChristopherGoushcha, OlegSmall, DavidGouveia, Élvio RúbioSmaragdakis, CostasGouwanda, DarwinSmetana, MilanGowda, Siddabasave B.Smeu, EmilGozalo, Guillermo ReySmida, BesmaGozdowski, DariuszSmirnov, AlexanderGrabham, NeilSmith, BethGrace, PaulSmith, Cory M.Grachev, AndreySmith, GraemeGraczykowski, BartlomiejSmith, Scott R.Grafton, MilesSmith, StewartGragnani, Gian LuigiSmith, ZacharyGraham, E. ScottSmoląg, KlaudiaGranda, Juan CarlosSmulders, KatrijnGrangeat, PierreSmulko, JanuszGrasa, EduardSmutny, VladimirGrassi, MarcoSmyl, DannyGrassi, SilviaSnoeys, WalterGRASSO, NIVESSnow, NicholasGratkowski, StanislawSo, JaewooGrattieri, MatteoSo, JungminGrau, AntoniSo, Kwok KanGravina, RaffaeleSoar, JeffreyGray, AndrewSoares Teles, ArielGray-Miceli, DeannaSoares, FabrízzioGrazia, Carlo AugustoSoares, Vasco N. G. J.Grbovic, DragoslavSoares, Vasco N.G.J.Greco, AlbertoSobaszek, MichałGreco, AntonioSobieranski, Antonio CarlosGreen, BrendaSobral, Andrews CordolinoGreen, David R.Sobrino, Xosé Antón VilaGreene, Brian R.Sobron, IkerGreener, JesseSobue, ShinichiGreenman, JohnSobus, JanGregor, CarolaSocoró Carrié, Joan ClaudiGregoretti, CarloSodhro, Ali HassanGrela, JakubSodnik, JakaGrella, MarcoŠofer, MichalGreminger, MichaelSohaib, MuhammadGric, TatjanaSohel, FerdousGriffin, JamesSo-In, ChakchaiGriffin, RobertSoininen, Juha-PekkaGriffith, HenrySöken, Halil ErsinGriffiths, ErinSokolov, OlegGriffiths, PeterSola-Guirado, Rafael RubenGriggio, FlavioSolanas, AgustiGrigorie, Teodor LucianSolar, HectorGrill, RomanSolari, FabioGrippo, ValentinaSolari, LorenzoGrisel, MichelSoldatenko, SergeiGrivokostopoulou, FoteiniSoldatos, JohnGrobelnik, MarkoSoleimani, ManuchehrGrobnic, DanSoler, MariaGrochla, KrzysztofSolhusvik, JohannesGroen, PimSoliman, HamdyGrolinger, KatarinaSolis Alfaro, JorgeGromek, DamianSolitro, GiovanniGrondin, FrancoisSolmaz, GürkanGrosch, AnjaSolodov, IgorGross, BarrySolovev, Denis B.Grossi, GiancarloSoltani, AminGrossi, MarcoSoltani, FouziGrosso, MichelangeloSoman, RohanGroves, RogerSomma, RobertaGroza, RobertSomnath, SuhasGruber, Allison H.Somov, YevgenyGrubić Kezele, TanjaSon, DongheeGrudzień, KrzysztofSon, JongsangGruev, ViktorSon, JunggabGruić, IgorSon, Tae YunGrulkowski, IreneuszSong, AiguoGrum, JanezSong, CaixiaGruppioni, EmanueleSong, ChaolongGrzechca, DamianSong, ChaoyunGrzes, TomaszSong, ChengGu, BoSong, ChunqiaoGu, ChenSong, FeiGu, FuqiangSong, GangbingGu, FuxingSong, HominGu, HaichangSong, HoubingGu, JingjingSong, Hyun SeokGu, KaiSong, Hyun-CheolGu, QingSong, Jae-bokGu, ShengfengSong, Jin WooGu, WentingSong, JizhouGu, YingkuiSong, JunesolGU, YUSong, Jung-IlGu, ZhaoquanSong, KaichenGualandi, IsaccoSong, KechenGualtieri, GiovanniSong, MeipingGuan, HaiyanSong, MinkyuGuan, LeSong, RanGuan, LianwuSong, Sa-KwangGuan, QuanshengSong, ShaleiGuan, YongSong, ShuangGuan, YuSong, Sung-JinGuaragnella, CataldoSong, Taek LyulGuarnieri, AlbertoSong, WeiGuascito, Maria RacheleSong, WuzhouGuccione, PietroSong, XiaoyuGucma, MaciejSong, YafeiGudimetla, PrasadSong, YongzeGudoshnikov, SergeySong, YoujianGüemes, AlfredoSong, You-jinGuerra, AnnaSong, Young MinGuerra, Elidia MariaSong, YuGuerra, Maria LetiziaSong, YuanpingGuerra, VictorSong, ZhanGuerrero, CarlosSonkar, KanchanGuerrero, Esteban MartínezSonobe, ReiGuerrero-Castellanos, FermiSood, KeshavGuerrero-Castillanos, FermiSookhak, MehdiGuerrero-Ginel, José EmilioSoomro, Toufique AhmedGuerrero-Ibáñez, Juan AntonioSoon, Jong JeongGuerrero-Rodríguez, José-MaríaSophocleous, MariosGuerrier, StephaneSoria, Pablo RamonGuerrier, StéphaneSoriano, AntonioGuerrieri, AntonioSorici, AlexandruGuglielmino, SalvatoreSorli, BriceGui, GuanSorohan, StefanGui, LinqingSorrentino, MarcoGuida, DomenicoSośnica, KrzysztofGuida, MarcoSossa, HumbertoGuidi, FrancescoSotelo Monge, Marco AntonioGuido, GIuseppeSotiriadis, PaulGuidotti, AlessandroSotner, RomanGuil, NicolasSoto Rodriguez, Paul Eduardo DavidGuimarães, Frederico GadelhaSoto, IgnacioGuiotto, AnnamariaSoto-Cámara, RaúlGuivant, JoseSoukis, KonstantinosGulati, KapilSoulis, KonstantinosGuldiken, RasimSouravlas, StavrosGulić, MarkoSousa Lima, WesllenGulrez, TauseefSousa, Armando JorgeGunawardana, UpulSousa, InêsGundavarapu, Sarat ChandraSouza, Eduardo G.Gun'ko, YuriiSouza, Francisco A. A.Günlü, OnurSouza, Victor HugoGüntner, AndreasSpachos, PetrosGuo, BaofengSpagnol, SimoneGuo, Chin-LinSpagnolo, VincenzoGuo, FeiSpanakis, Elias K.Guo, HangSpanakis, Emmanouil G.Guo, HongzhiSpano', AntoniaGuo, JiajieSpecht, MariuszGuo, JianhuaSpeck, Andrew J.Guo, JianlinSpecogna, RubenGuo, JimingSpencer, Daniel CGuo, JinhuaSpengler, Anderson WedderhoffGuo, Jiun-InSperanza, DomenicoGuo, LiSperanzini, EmanuelaGuo, LingzhongSperlì, GiancarloGuo, LuSpezzano, GiandomenicoGuo, PeixuanSpezzi, LoredanaGuo, QiandongSpiers, AdamGuo, QingboSpigulis, JanisGuo, ShuangzhuangSpikol, DanielGuo, WeiSpiliotopoulos, MariosGuo, XiangguiSpina, RobertoGuo, XianshengSpinelli, NicolasGuo, XiaoliangSpingler, BernhardGuo, XinfeiSpinsante, SusannaGuo, YaoSpivak, ArthurGuo, ZhiqianSpivak, ArturGuo, ZhouSpizzichino, ValeriaGupta, DeepaSpohr, Klaus MichaelGupta, InderSpoladore, DanieleGurban, Ana-MariaSpolaor, FabiolaGurtov, AndreiSportelli, Maria ChiaraGustavo, CallicoSpratling, MichaelGut, KazimierzSpringer, ShmuelGutiérrez Guerrero, Jóse MiguelSpringmann, John C.Gutiérrez Reina, DanielSpronck, JoGutiérrez, EdgarSqueglia, FlaviaGutiérrez, J. JavierSreekumari, PrasanthiGutierrez, JairoSresht, VishnuGutiérrez, JonSrinivasan, KathiravanGutiérrez, Juan ManuelSrinivasan, SrikantGutierrez, Miguél ÁngelSriram, RashmiGutiérrez-Capitán, ManuelSriramdas, RammohanGutierrez-Heredia, GerardoSrivastava, GautamGuttieri, Mary J.Srivastava, VibhaGuyonneau, RémySrivastava, VishwasGuzik, AgnieszkaSroka, RyszardGuzman, MarceloSrpak, DunjaGuzman-Chavez, Ana DinoraStaack, IngoGuzman-Quesada, JoseStaal, Odd MartinGuzman-Sepulveda, Jose RafaelStai, EleniGuzowski, BartłomiejStajanca, PavolGwak, JeonghwanStamatelos, Anastassios M.Gwenzi, DavidStamatescu, GrigoreGyrard, AmelieStamatopoulos, Constantine A.Ha, Dong SamStamenkovic, ZoranHa, IlkyuStamm, AndyHa, MinjeongStamnes, SnorreHa, SohmyungStan, MirunaHaack, BarryStancic, IvoHaas, RüdigerStančin, SaraHaas, ZygmuntStanciu, AlexandruHaase, Karl B.Staniek, MarcinHabib, Md SelimStanko, DavorHabisreuther, TobiasStanko, StephanHachaj, TomaszStankovski, VladoHachour, SamirStanojevic, Dragana M.Hackett, MarkStarecki, TomaszHadachi, AmnirStarek, MichaelHadaś, TomaszStaruch, MargoHadas, ZdenekStasiewicz, KarolHaddad, AssedStateczny, AndrzejHaddadi, KamelStateczny, Andrzej EugeniuszHadid, AbdenourStatkiewicz-Barabach, GabrielaHadjiiski, LubomirStauffer, FlurinHäfliger, PhilippStavrakis, ModestosHager, IzabelaStavrakis, StavrosHaghparast, MajidStavroulakis, Georgios E.Hagmüller, MartinStawiarski, AdamHahm, Sung HoStawska, HannaHahn, AxelSt-Charles, Pierre-LucHahn, Jin-OhSteele, John P HHahn, MichaelStefan, OvidiuHai, GuoqiangStefanescu, AlexandraHaidegger, TamasStefani, LauraHaider, GolamStefanini, LucianoHaider, Mohammad RafiqulStefanov, AndrejHaidry, Azhar AliStefanov, KonstantinHailu, GetuStefańska, PatrycjaHainberger, RainerSteffert, TonyHaitjema, HanStegall, PaulHaiyong, LuoStein, AlfredHajder, LeventeSteiner, Benedikt M.Hajduch, GuillaumeSteinhauer, StephanHajigholizadeh, MohammadStellavato, AntoniettaHajimirsadeghi, MohammadStemler, ThomasHakala, IsmoStepanian, Phillip M.Håkansson, JohanStepanov, Oleg A.Hakem, NadirStepanyan, KarenHakonen, AronStępień, GrzegorzHakulinen, JaakkoStepien, JacekHalder, ArnabStepien, KrzysztofHalder, Sharly JoanaStepinski, TadeuszHalilaj, EniSternberg, HaraldHalim, Md AbdulStetter, RalfHalkon, BenSteup, MartinHall Barbosa, Carlos RobertoStevan Jr., Sergio L.Hallaji, MiladStevens, NobbyHaller, ArminStevens-Navarro, EnriqueHallewell Haslwanter, Jean D.Stewart, ThomasHallik, LeaSthal, FabriceHallil, HamidaStienne, GeorgesHalls, JoanneStiens, JohanHaluska, MiroslavStipanicev, DarkoHalvorsen, KjartanStiros, Stathis C.Ham, SuyunStochino, FlavioHamacher, DennisStocker, MarkusHämäläinen, JoonasStodola, PetrHamann, EliasStoean, CatalinHamdar, SamerStoean, RuxandraHamel, MatthieuStoimenov, LeonidHamilton, AndrewStojcsics, DanielHammerstrom, Donald J.Stolarczyk, AgnieszkaHamon, MorganStoop, Ralph L.Han, BinStopar, BojanHan, CaiqinStopforth, RiaanHan, ChongStoppa, DavidHan, CongzhengStorace, MarcoHan, Dai-InStorm, HanneHan, DezhiStornelli, VincenzoHan, FangStortini, AngelaHan, FengtianStory, BrettHan, Guo-ChengStoyanov, BorislavHan, Il SongStrakowski, MarcinHan, Jae-HungStrati, VirginiaHan, JunStraub, JeremyHan, JungheeStrazdins, GirtsHan, JungongStrazdins, PeterHan, KeStraže, AlešHan, KyoohyungStrazza, AnnachiaraHan, MengStreimikiene, DaliaHan, MengdiStreitferdt, DetlefHan, QingwenStrelniker, YakovHan, XianhuaStreque, JérémyHan, XiaohuiStreubel, RobertHan, XiaotaoStrigul, NickHan, XiuyouStrle, DragoHan, YahongStrohbach, MartinHan, YoukyungStrohm, EricHan, You-KyungStrokina, NataliyaHan, Young-GeunŠtroner, MartinHan, YoungjoonStroppa, FabioHand, EmilyStroulia, EleniHanink, Dean M.Strumillo, PawelHanke, StenStrzałkowski, KarolHanks, Ephraim M.Strzalkowski, NickHanley, BrianStrzelecki, MichałHann, ChristopherSTUERGA, DidierHanna, NaderŠturala, JiříHannink, JuliusŠtys, DaliborHannu, JariSu, Ching-LiangHanoune, BenjaminSu, DanHansard, MilesSu, GuoshaoHänsch, RonnySu, HangHänsel, AndreasSu, HongboHansen, ClintSu, JinyaHansen, JohnSu, JunweiHanson, Lars G.Su, LuHao, JianguoSu, Ming-ShouHao, QunSu, Pi-GueyHao, ZhaoSu, RongHao, ZhenhuaSu, RuoyuHapp, Patrick NigriSu, Ting-LiHara, ShinsukeSu, Tung-ChingHarada, YukihiroSu, Wen-HaoHaraguchi, AkiraSu, YanjunHaras, MaciejSu, YichiHare, JamesSu, YishanHarezlak, KatarzynaSu, YuanjieHarik, El Houssein ChouaibSu, ZhiqiangHariri-Ardebili, M. AminSu, Zhong QingHarizanov, StanislavSuarez, AlvaroHarja, MariaSuarez, FranciscoHarley, Joel B.Suárez-Casal, PedroHarmanci, Yunus EmreSubr, KarticHarmel, TristanSubramanian, SenthilHarris, NickSućeska, MuhamedHart, AdamSucharda, OldrichHart, JustinSuchorab, ZbigniewHartanto, AndreeSuciu, GeorgeHartanto, RonnySudduth, KennethHartlieb, PhilippSuematsu, KoichiHartwig, ValentinaSuess, BeatrixHaruyama, TetsuyaSuess, DieterHarvey, AndrewSuess, Ryan J.Hasan, Khondokar FidaSugden, KateHasegawa, GoSuh, JangwonHasegawa, HideyukiSuh, Young SooHasegawa, KeisukeSuhanek, MiaHasegawa, YoshihiroSuhling, KlausHashemi, EhsanSuhr, Jae KyuHashemi-Beni, LeilaSui, HaigangHashishin, TakeshiSui, XiaohongHasimoto-Beltran, RogelioSuicmez, Vural SanderHassan, EmadeldeenSujecki, SlawomirHassan, FirasSukhov, VladimirHassan, Quazi K.Sukthankar, Gita ReeseHassanalian, MostafaSulai, IbrahimHassapis, GeorgiosSulewski, PiotrHatsukade, YoshimiSullivan, KeithHattori, ReijiSułowicz, MaciejHattori, YoshihideSulyman, Ahmed IyandaHatzikraniotis, EvripidisSulzer, JamesHatzivasilis, GeorgeSum, Yee LoonHaupt, MichaelSun, BaoqiHausman, SławomirSun, BaoqingHavens, Kathryn LSun, ChengmingHavlík, JanSun, Chung-HsunHavran, LudekSun, Daniel (Jian)Hawash, ZaferSun, FangminHawbani, AmmarSun, GongchenHawkins, BrianSun, GuanghaoHaworth, JamesSun, GuanghuiHayakawa, YuichiSun, HaijianHayashi, NeiseiSun, HaixinHayat, MunawarSun, HanjunHaycock, BruceSun, Hong-TaoHazrati, MehrnazSun, HuchengHe, BingweiSun, HungHe, ChengbingSun, JianchunHe, DapingSun, JiandeHe, DiSun, JiazhenHe, DongfengSun, JinpingHe, DongjianSun, JinweiHe, FangningSun, Kien-WenHe, HuSun, LiHe, HuijingSun, LinHe, JiayuanSun, MeijunHe, JiboSun, MengHe, JinSun, MingjianHe, Jing-SelenaSun, Ming-JieHe, JingjingSun, MinhongHe, JinxinSun, RuopengHe, limingSun, Shih-WeiHe, QingsongSun, ShuqingHe, SuiningSun, WeiHe, WeiSun, WeiweiHe, WeifengSun, WujinHe, XianqiangSun, XiankaiHe, YaqianSun, XuanHe, YiSun, YanHe, YihaiSun, YanhuaHe, YijunSun, YanjingHe, YuanSun, YankuiHe, YunzeSun, YongliangHe, ZhenyuSun, YuanxiHe, ZongjianSun, Yun-LuHeadland, DanielSun, Yun-PingHeadley, WilliamSun, YunxuHeal, MathewSun, YuqiongHeartfield, RyanSun, ZheHeberger, KarolySun, ZhiyongHedegaard, BrockSundararajan, RajiHegde, NagarajSundaravadivel, PrabhaHeijnen, Petra W.Sundberg, RobertHeikkilä, TapioSung, Guo-mingHeimfarth, TalesSunny, Ali ImamHein, JoachimSuomi, IreneHeindl, RankoSuppa, AntonioHeinrich, Mattias P.Surace, CeciliaHeins, BradleySurawski, NicHeintzman, LarkinSuresh, Mahima AgumbeHeise, Herbert MichaelSurinta, OlarikHeiselberg, HenningSuryadevara, Nagender KumarHeiskanen, JanneSusnea, IoanHejduk, LeszekSutin, AlexanderHelebrandt, PavolSutlief, ArinHella Ellen, AhrendsSužiedelytė-Visockienė, JūratėHellack, BryanSuzuki, ArataHellbrück, HorstSuzuki, TaroHellman, AmySuzuki, YoshioHellwagner, HermannSuzurikawa, JunHelmer, SvenŠvanda, MilanHelmi, GhanmiSvatek, VojtechHelseth, LarsSvecko, RajkoHelske, JouniSvedberg, GustavHemmati, FarzadSvensson, Carl-MagnusHemsel, TobiasSvilainis, LinasHenarejos, PolSvoronos, Paris D.N.Henderson, RobertSweetman, MartinHendon, ChristineŚwiderski, WaldemarHendrick, PatrickŚwięch, ŁukaszHendrie, JamesŚwit, GrzegorzHengy, SebastienSyafrudin, MuhammadHenley, RobertSydänheimo, LauriHenriques, Felipe Da RochaSylwestrzak, MarcinHenriques, RenatoSynhaivska, OlenaHenriques, Renato FilipeSynnott, JonathanHenry, DaveSyritski, VitaliHenry, ManusSystä, KariHeo, JoonSzabo, AlexandreHeo, MoonbeomSzabo, LorandHeo, Yun JungSzabolcsi, RóbertHer, Shiuh ChuanSzantoi, ZoltanHeras, JónathanSzaszák, GyörgyHerbin, StéphaneSzatyłowicz, JanHerceg, MarijanSzczerska, MalgorzataHerkommer, AloisSzczerska, Malgorzata JędrzejewskaHerle, StefanSzczesna, AgnieszkaHerlitzius, ThomasSzczęsny, SzymonHermens, FroukeSzczuko, PiotrHermosilla, GabrielSzeleszczuk, ŁukaszHernaez, MiguelSzerement, JustynaHernandes, Andre CarmonaSzermer, MichalHernández Creus, AlbertoSzeto, Grace P. Y.Hernández, Ana L.Szewczyk, RomanHernández, David NaranjoSziebig, GaborHernandez, IvanSzkaliczki, TiborHernandez, MonicaSzostak-Chrzanowski, AnnaHernández, NicolásSztajnberg, AlexandreHernandez, NoeliaSztyber, AnnaHernandez, SarahSzulczyński, BartoszHernández, VicenteSzulwic, JakubHernandez-Escobedo, QuetzalcoatlSzwoch, GrzegorzHernández-Orallo, EnriqueSzwoch, MariuszHernández-Ramos, José L.Szymak, PiotrHernández-Romano, IvanSzymanik, BarbaraHernández-Serrano, JuanSzymanska-Chargot, M.Hernando, DavidSzymborski, TomaszHerrera, Jesus Antonio SosaSzypłowska, AgnieszkaHerrera, ManuelT. Delaney, DeclanHerrera-Alcántara, OscarTabar, Yousef RezaeiHerrera-López, Enrique J.Tabassum, ShawanaHerrera-May, Agustin L.Tabatabaeipour, MortezaHerrero Vinias, PauTabib-Azar, MassoodHerrero, Elías RevestidoTabish, TanveerHerrero-Agustín, José-LuisTaboada, FernandoHerrmann, IttaiTaborri, JuriHerrojo, CristianTadayon, RaminHerron, Jeffrey A.Tadepalli, SirimuvvaHess-Dunning, AllisonTadesse, MelkieHesselbach-Serra, XavierTaebi, AmirtahaHeuer, HenningTaebi, AmirtahàHey, JonathanTaghizadeh, OmidHeydari, ShahramTagliatesta, PietroHiblot, GaspardTaglietti, AngeloHida, HidekiTague, PatrickHidalgo López, José AntonioTahara, HironobuHidalgo, J. IgnacioTaheri, HosseinHielscher, Kai-SteffenTaheri, SaiedHigo, AkioTaherizadeh, SalmanHilburn, Kyle A.Tai, HuilingHilkert, James M. (Jim)TAIAR, RedhaHill, JonathanTakabayashi, KentoHill, RichardTakagi, ToshiyukiHill, VictoriaTakahashi, KazunoriHiller, DanielTakahata, TomoyukiHiller, MatthiasTakala, JosuHina, Manolo DulvaTakashima, KazutoHinkov, BorislavTakeda, RyoHinks, JamieTakei, MasahiroHino, HideitsuTakeishi, NaoyaHinojo, Jose MaríaTakemura, KenjiroHinostroza, IsraelTaki, MasayasuHippler, RainerTakyi, GabrielHirmer, PascalTal, IrinaHiromori, AkihitoTalaat, AhmedHiromoto, R.E.Talaśka, TomaszHiryu, ShizukoTaleb, AbdelhafedHjelme, Dag RoarTalhi, OualidHlaváčová, Irena M.Talla, VamsiHlubina, PetrTallman, Tyler NHmam, HatemTallman, Tyler N.Hmila, MariemTalone, MarcoHnilicka, FrantisekTalvitie, JukkaHo, Aaron H.P.Tamagnone, MarioHo, Chong PeiTamari, SergeHo, Jun-WonTamarin, OllivierHo, Ko-ShanTamazin, MohamedHo, Siu WaiTamazin, Mohamed E.Ho, Tan-JanTamberg, GertHoang, DoanTames, JoseHoath, StephenTamilia, EleonoraHobiger, ThomasTamm, TarmoHochberger, ChristianTamošiūnas, VincasHochreiner, ArminTamulevičius, TomasHodge, VictoriaTamura, HirokiHoffmann, DanielTan, Bee KangHoffmann, JanTan, BoHoffmann, MatejTan, ChaoHofhansl, FlorianTan, Chee H.Höflinger, FabianTan, Chin YawHOLADE, YaoviTan, HaowenHolak, KrzysztofTan, KunHölbl, MarkoTan, Le ThanhHölbling, DanielTan, LianshengHolder, LawrenceTan, SabineHolland, Charles W.Tan, ShengHollander, KarstenTan, Yew TeckHoller, StephenTan, YihuaHolobaca, Iulian HoriaTan, ZhengguoHolst, AndersTanabe, TadaoHolst, ChristophTanaka, HiroshiHolub, MichalTanaka, NaoroHolzinger, AndreasTanaka, ShigehoHomayouni, SaeidTang, FangfangHonaker, Lawrence W.Tang, FeiHondongwa, DonaldTang, HongHonegger, DavidTang, JinjunHoneychurch, KevinTang, JunHong Lin, ChangTang, JunqingHong, Cheng-YihTang, LinHong, FengTang, LongtengHong, Ic-PyoTang, MingHong, JaesungTang, Shiang-FengHong, Jang-EuiTang, Shih-tsangHong, Jin B.Tang, SuhuaHong, Sang-HoonTang, WeiHong, SanghyunTang, WenlongHong, Sun K.Tang, WenwuHong, Sung KyungTang, WilkinHong, Wei-ChiangTang, XiaojunHong, YangTang, XuHong, YuningTang, YingganHong, ZhonghuaTang, YunchaoHönnicke, Marcelo GonçalvesTang, Yunchao TangHonório, Leonardo De MelloTang, ZhanyongHontañón, EstherTang, ZhichuanHooshmand, MohsenTang, ZhifengHopmans, JanTanganelli, GiacomoHoppe, NiklasTangirala, Venkata Krishna KarthikHopper, RichardTango, FabioHorak, EmaTangoulis, VassilisHorauer, MartinTani, HirofumiHordyniec, PawełTaniai, TatsunoriHoremuz, MilanTaniguchi, MasateruHorgan, GrahamTaniguchi, Rin-ichiroHoriguchi, TsuneoTankasala, PavanHorla, DariuszTanno, TakenoriHornak, JaroslavTanyeri, MelikhanHorne, MalcolmTao, JunHorng, Gwo-JiunTao, LeiHorng, Shih-ChengTao, MingHorng, Shi-JinnTao, TianyouHorng, Tzyy-ShengTao, XiaofengHorrillo Güemes, CarmenTao, XinHorswill, Craig ATao, XuyuanHortal, EnriqueTapete, DeodatoHorvat, RobertTapu, RuxandraHorváth, JuditTar, JozefHorvath, TomasTarabini, MarcoHoseinnezhad, RezaTarantino, CristinaHosek, JanTarantino, EufemiaHoshiba, KotaroTarantino, LauraHosoi, FumikiTarasov, AlexeyHossain, AkbarTarasov, Sergei YuHossain, M.NurTaravat, AlirezaHossain, MahmudTardif, Pierre MartinHossain, Mohammad JobayerTarnita, DanielaHossein-Zadeh, ManiTasca, FedericoHosseinzadeh, MehdiTashakori, ShervinHossny, MohammedTashima, ToshihikoHosu, OanaTatar, ErdincHoth, JulianTate, DanielHotovy, IvanTate, Rothwelle J.Hou, JingmingTatlas, Nicolas-AlexanderHou, Kun-meanTatullo, MarcoHou, Ting-ChaoTavakkoli, AlirezaHou, WenshengTavernini, DavideHou, XiaoleiTayara, HilalHou, YanxiaTayeb, ShahabHou, YunfeiTaylor, Matthew E.Houben, SebastianTaylor, AndyHowcroft, JenniferTaylor, CianHowe, Bruce M.Taylor, ClarkHowes, PhilipTaylor, HaydenHożyń, StanisławTaylor, MichaelHrad, JaromirTaylor, Michael A.Hradiš, MichalTcheou, Michel P.Hrgetić, MarioTeal, PaulHristoforou, EvangelosTecpoyotl-Torres, MargaritaHsia, Chih-HsienTeghil, RobertoHsia, Shao-YiTegos, AristotelesHsiao, Fu YuenTeh, AndrewHsiao, Fu-yuenTehrani, FarazHsiao, Wen-TseTeichmann, DanielHsieh, Chang-YuTeixeira, Adunias Dos SantosHsieh, Cheng-HsiungTeixeira, AntónioHsieh, GaryTeixeira, Fernando L.Hsieh, Hung-LinTeixeira, Francisco CuradoHsieh, Ping-HsuanTeixeira, Marcos F.S.Hsieh, Tung-HsunTejado, InésHsieh, Wen-ChingTelada, SouichiHsu, Chih-MingTello-Oquendo, LuisHsu, Huan-HsuanTemiz, YukselHsu, Li-TaTempesta, GioacchinoHsu, Quang-CherngTen Bhömer, MartijnHsu, Shun-HsiTenace, ValerioHsu, Ting-YuTeng, Kah HouHsu, Wei-ChunTenhunen, MirjaHsu, Wei-YenTennakoon, RuwanHu, BinTennina, StefanoHu, BoyiTeo, EJHu, ChuanTeodoro, Ana CláudiaHu, ChunxiaoTeodoro, Paulo EduardoHu, FengTera, MelaniaHu, GuohuaTeranishi, NobukazuHu, GuoqingTerebes, RomulusHu, HanTerefenko, PawełHu, HongliTerpenny, JanisHu, HongpingTerrazas Angulo, GermanHu, JianjunTerze, ZdravkoHu, JiankunTessarolo, MartaHu, JingtianTestoni, NicolaHu, JiweiTetteroo, DanielHu, JunTeucke, MichaelHu, JunhuiTeunissen, PeterHu, LiangTeymoori, PeymanHu, NingTeymourian, HazhirHu, QingwuThai Duy, LeHu, ShaohanThakker, DhavalkumarHu, SijungThakur, GarimaHu, WeiduoThakur, UjwalHu, XianbiaoThang, Tran QuyetHu, XiangyunThanh, Kien NguyenHu, XiaogongTharmarasa, RatnasinghamHu, XiaosuThemistocleous, KyriacosHu, XiaotaoTheodorakopoulos, GeorgeHu, XinghaoTheodorakopoulos, IliasHu, YaxiTheodoridis, EvangelosHu, Yuh-ChungTheodosiou, AntreasHu, YunpuTheorell, ToresHU, Zhan-YiTheuwissen, AlbertHu, ZhaozhengThevenon, HerveHu, ZhirunThevenot, JérômeHua, LiThiamwong, LaddaHua, XugangThielecke, JörnHuang, BanqiThielemann, ChristianeHuang, ChangchunThielens, ArnoHuang, Chester S. J.Thieme, Christoph AlexanderHuang, Chih-HsienThirion-Lefevre, LaetitiaHuang, Chih-WeiThiyagarajan, Jothi SaravananHuang, Ching-ChunThom, ChristianHuang, Ching-JerThomas, DiegoHuang, Chiou-JyeThomas, Jean BaptisteHuang, Chi-YoThomas, Jean-BaptisteHuang, Chun-HsianThorleuchter, DirkHuang, DongThornburg, JonathanHuang, FamingThornton, BlairHuang, FayThramboulidis, KleanthisHuang, Genin GaryThuau, DamienHuang, GuangyanThurow, KerstinHuang, GuanwenTian, ChaoHuang, HailongTian, DaxinHuang, HaipingTian, Gui YunHuang, HaocaiTian, HeHuang, Helen J.Tian, HongdaHuang, HuaweiTian, JiangweiHuang, HuiTian, QinglinHuang, Hung JiTian, ShiyiHuang, JennyTian, XiaoyongHuang, JianqiuTian, YingweiHuang, JiaruiTian, YingzhongHuang, JieTian, YonghuiHuang, Ji-JerTian, ZhenhuaHuang, JingfengTiana, DavideHuang, JingyiTibaduiza Burgos, Diego AlexanderHuang, JiyanTiberi, ChristelHuang, JunTiberi, GianluigiHuang, LianfenTibor, ChovanHuang, LiangTiebe, CarloHuang, LimingTiemann, JanisHuang, LinTiemann, MichaelHuang, MingfengTietze-Jaensch, HolgerHuang, Ming-ShyanTime, Rune WHuang, Nien-TsuTimmons, Adela C.Huang, Pei-ChiTinarelli, RobertoHuang, PengdaTirado-Mendez, Jose AlfredoHuang, Po-TsangTirronen, TuomasHuang, QijinTiseira, Andrés OmarHuang, QijunTittmann, Bernhard RainerHuang, QinganTiu, Brylee DavidHuang, ShengxiTivarus, CristianHuang, Shih-ChangTivive, Fok Hing ChiHuang, ShipingTiwari, Kumar AnubhavHuang, SonglingTjondronegoro, DianHuang, SunanTkáč, JánHuang, TaoTkach, ItshakHuang, TianyaoTlelo-Cuautle, EstebanHuang, Ting-ZhuTobarra, LlanosHuang, Tzu-HaoTobe, YoshitoHuang, WeiminTobella, Aida BarguésHuang, WenbinTobon, JorgeHuang, XianToczylowska-Maminska, RenataHuang, XueqingTodt, EduardoHuang, YanboToffanin, ChiaraHuang, YanjunToffoli, DanieleHuang, YingTognetti, AlessandroHuang, YiqingToivonen, JuhaHuang, Yo-PingTokarz, DanielleHuang, YuToker, OnurHuang, YueTokuda, TakashiHuang, Yu-HsiTol, SerifeHuang, Yung-FaToledo, JonayHuang, ZacharyTollefsen, DagHuber, ChristianTollner, Ernest W.Hudec, RobertTomas-Burguera, MiquelHudoklin, DomenTomasi, BeatriceHughes, BenTomasic, IvanHughes-Riley, TheodoreTomasz, RymarczykHuguet, Silvia SoriaTomaszewski, DariuszHuh, JungwonTomažič, SimonHuh, Jun-HoTomer, Vijay K.Huh, Yun SukTomic, SlavisaHui, JieTomita, Jesuino TakachiHui, PatrickTomkowski, RobertHui, YilongTomšič, BrigitaHuignard, Jean-PierreTomsik, ElenaHulka, KarelTon, Shan-ShinHumar, MihaTonacci, AlessandroHung, Ho-LungTondini, StefanoHung, Jui-chungTonelli, DomenicaHung, King FaiTong, FeiHung, Li-WeiTong, FengHung, Ming-ChihTong, JinwuHung, Yu-HanTong, KennethHuo, Lian-ZhiTong, LiangHuo, LinshengTong, Sheau-RuHuo, XingliangTong, YexiangHuo, YimingTönges, LarsHuotari, MattiTonoli, AndreaHur, KyeongTopp, Elin AnnaHur, ShinTopputo, FrancescoHura, TomaszToprakci, Hatice Aylin KarahanHurley, Ryan CToral-Cruz, HomeroHursthouse, Andrew STöreyin, HakanHurtík, PetrTornabene, FrancescoHussain, AmjadTornari, ViviHussain, FatimaTornil-Sin, SebastianHussain, GhulamTörök, ÁkosHussain, Suhail S.M.Torralba, AntonioHussain, Syed RafiulTORRES, LizethHussain, ZahirTorres, Pedro M. B.Hussein, HusamTorres, RoqueHussein, MoustafaTorres, SergioHussein, RamyTorres-Cisneros, MiguelHussein, SarfarazTorres-Diaz, IsaacHuston, DryverTorres-Gomez, IsmaelHutter, TanyaTorres-Mapa, Maria LeilaniHuttunen, HeikkiTorres-Ramírez, EduardoHuttunen, JanneTorres-Roman, Deni LibradoHutu, Florin DoruTorres-Rua, AlfonsoHuynh, Thanh-CanhTorres-Sospedra, JoaquínHuynh-The, ThienTorres-Torres, ReydezelHwang, Cheong-SooTorti, EmanueleHwang, Chi-HungTortschanoff, AndreasHwang, Chi-SunTorun, HamdiHwang, Dong-HwanTosato, PietroHwang, Geon-TaeTosello, GuidoHwang, GraceToshilhiro, TsujiHwang, Jae YounTosi, DanieleHwang, Jung-hwanTóth, TündeHwang, Keum-CheolTóth, ZsoltHwang, Ren-HungTouhafi, AbdellahHwang, Ruey-BingTountas, KonstantinosHwang, Suk-WonToups, Z O.Hwang, SungjaeTourani, RezaHwang, Wan SikTouryan, JonathanHwang, Wen-JyiToyoura, MasahiroHwang, Wen-LiangTrainiti, GiuseppeHwang, YoungbaeTrakic, AdnanHwu, Jenn-GwoTramarin, FedericoHyakawa, YuichiTran, Binh Q.Hyrynsalmi, SamiTran, DatHyun, EuginTran, Duy PhuHyun, Kyung-ATran, Gia KhanhHyunjo, JeongTran, Kim PhucIacoviello, DanielaTran, Minh AnhIadicicco, AgostinoTran-Dang, HoaIakovidis, DimitrisTraulsen, ImkeIammarino, MarcoTravagliati, MarcoIano, YuzoTraver, VicenteIban, Antolín LorenzanaTravieso-González, CarlosIbarra-Arenado, ManuelTreiber, MartinIbarra-Manzano, OscarTreister, EranIce, Si-YinTrejo, LuisIchihashi, RyujiTrendafilova, IrinaIchikari, RyosukeTresanchez, MarcelIchikawa, KoheiTriboulet, JeanIdzkowski, AdamTrigo, Flavio CelsoIeng, Sio-SongTrigo-Rodríguez, Josep M.Ientilucci, EmmettTrihinas, DemetrisIero, DemetrioTrimmel, MichaelIglesias Gallego, DamiánTrinder, JohnIglesias Rodríguez, RobertoTripathi, ManojIglesias, Carlos A.Tripathi, RenuIgnjatovic, AleksandarTripicchio, PaoloIgoe, DamienTripoliti, Evanthia E.Igoe, DavidTrivinho-Strixino, FranciscoIgual, JorgeTriviño, AliciaIgual, RaulTroglia Gamba, MicaelaIhm, InsungTroia, BenedettoIida, DaisukeTroisi, SalvatoreIizuka, KotaroTroncone, AntonelloIkeda, AkihikoTroncoso-Pastoriza, FranciscoIkehashi, TamioTronstad, ChristianIkiades, ArisTrontelj, JanezIkonomidou, Vasiliki N.Trotta, AngeloIlankoon, SamanTrouillon, RaphaëlIliev, IvoTrovati, MarcelloIliyasu, Abdullah M.Trucco, AndreaIlovitsh, TaliTrujillo-León, AndrésIluk, ArturTrujillo-Schiaffino, GerardoIlyakov, I.E.Trung, Ngo ThanhIlyas, AzharTrung, NgoThanhImai, NiltonTruong, LongImaki, MasaharuTruong, NguyenImamura, GakuTruong-Hong, LinhImani, MahdiTrusiak, MaciejImasu, RyoichiTrusso Sfrazzetto, GiuseppeImbiriba, TalesTsagaris, ApostolosImmich, Roger KreutzTsagkaris, AristeidisImoto, HiorakiTsai, Chi-YiImpedovo, DonatoTsai, Kun-LinImperatore, PasqualeTsai, Pang-WeiImtiaz, Masudul HaiderTsai, Pei-HsuanImtiaz, Syed AnasTsai, Pei-WeiInácio, Pedro R. M.Tsai, Pei-YunIndri, MarinaTsai, Yao-HongIndurkhya, BipinTsai, Yao-TeInga, EstebanTsai, Yi-JuInnocent, ManuelTsai, Yu-TingIno, KosukeTsakanikas, PanagiotisInoue, JunichiTsaklis, PanagiotisInuso, GiuseppinaTsamados, MichelInvernizzi, StefanoTsamis, ChristosInzerillo, LauraTsang, Kim FungInzillo, VincenzoTsangouri, EleniIoakimidis, ChristosTsarchopoulos, PanagiotisIoana, CornelTsarev, AndreiIoannidis, CharalabosTseng, Fan-HsunIoannidis, DimosthenisTseng, Victor Farm-GuooIoannidou, AlexandraTseng, Yuan-ChiehIocchi, LucaTsiakas, KonstantinosIodice, AntonioTsiartas, AndreasIonescu, Elena RodicaTsigaridas, GeorgiosIonete, Eusebiu IlarianTsiigkaridis, John (Ioannis)Iqbal, Hafiz M. N.Tsiligiridis, Theodore A.Iqbal, Hafiz. M. NTsinidis, GrigoriosIqbal, UmarTsinos, Christos G.Ireland, ChristopherTsioliaridou, AgelikiIrrera, FernandaTsipouras, Markos G.Irusta, UnaiTsiropoulou, Eirini EleniIsakov, DmitryTso, RaylinIsernia, TommasoTsogka, ChrysoulaIsgrò, FrancescoTsoukalas, DimitrisIshida, HiroshiTsoumanis, GeorgiosIshikawa, DaitaroTsouti, VasilikiIshikawa, JunTsoutsouras, VasileiosIskierko, Zofia EwaTsow, Francis (Tsing)Islam, Kh TohidulTsubaki, ShuntaroIslam, M M ManjurulTsuchiya, Ken'ichiIslam, MahfuzulTsuji, ToshiakiIslam, SalekulTsuji, YutakaIslam, ShamaTsujii, ToshiakiIsoaho, NooraTsunekawa, AtsushiIsola, GaetanoTSUTSUI, MakusuIsozaki, AkihiroTsutsumida, NarumasaIsvoranu, DragosTsuzuki, MarcosIta, KevinTsvetkov, PavelIto, AkinoriTsytsarev, VassiliyIto, TomohiroTu, ChengIto, YoshihiroTu, DongItrić, KatarinaTu, LiangchengItuero, PabloTu, MinIula, AntonioTu, Min-ChengIvan, LoredanaTu, RuiIvan, VladimirTu, ShanshanIvanov, DanilTuck, LoughlinIvanov, Ilia N.Tudorache, FlorinIvanov, RosenTUDOSIE, Alexandru-NicolaeIvanov, StepanTuhtan, Jeffrey A.Ivanova, Alla V.Tuinea-Bobe, CristinaIvanovici, MihaiTullis, JasonIvanovska, TatyanaTulpan, DanIvić, StefanTumarkin, AndreyIvković, NikolaTuna, CagdasIvorra, AntoniTung, Chun-WeiIwami, KentaroTung, Nguyen-DangIwata, TatsuyaTura, AndreaIyer, ChitraTuraga, PavanIyota, TaketoshiTuranoglu Bekar, EbruIzadyar, AnahitaTurchetta, RenatoIzaguirre, AlbertoTurchetti, ClaudioIzaguirre, Alberto CordobaTurchin, IlyaIžák, TiborTurcza, PawelIzquierdo Cordova, RamonTurdean, Graziella L.Izquierdo, Estibaliz MartinezTurgul, VolkanIzquierdo-Fuente, AlbertoTuriel, Javier PérezIzu, NoriyaTurk, GoranIzumi, Celly M.S.Turkmen, MustafaIzydorczyk, JacekTurkoglu, KamranJ.R. PHUA, EricTuzson, BelaJabari, ShabnamTwu, Ruey-ChingJabba, DaladierTylecek, RadimJabrane, YounesTysiac, PawelJacek, ŻmojdaTysiąc, PawełJachowicz, RyszardTyszczuk-Rotko, KatarzynaJacinto, TiagoTzallas, AlexandrosJackson, D. A.Úbeda, AndrésJackson, NathanUbertini, FilippoJacob, Maik H.Uccheddu, FrancescaJacobs, DanielUchiyama, HideakiJacobs, Jesse V.Uchiyama, KenjiJacobs, PeterUdalcovs, AleksejsJacyna-Gołda, IlonaUddin Ahamed, NizamJADIDI, ZahraUddin Ahmed, MobyenJaehne-Raden, NicoUddin, A.S.M. IftekharJafari, RahelehUdrea, AndreeaJafari, RoyUdroiu, RazvanJafarian, J. HaadiUeberschär, OlafJaffrey, SamieUemoto, JyunpeiJaffrezic-Renault, NicoleUgbolue, UkadikeJagannath, JithinUgo, PaoloJagiela, MariuszUgon, AdrienJagüey, Joaquín GutiérrezUhlmann, JeffreyJahangir, IfatUl-Abdin, ZainJahier Pagliari, DanieleUlapane, NalikaJaime, MarceloUlazia, AlainJaitner, ThomasUlbrich, DariuszJakieła, SławomirUllah, MohibJakimow, BenjaminUllo, SilviaJakli, AntalUllod, M. TeixidóJakob, GerhardUlrich, MarkusJakobson, ClaudioUlrich, SchwaneckeJakšić, OlgaUm, TaiwonJaksic, VesnaUmbaugh, ScottJakubik, Wiesław P.Umemoto, TakahiroJalal, AhmadUnderhill, Peter RossJalal, Dr. AhmedUngureanu, FlorinaJalali, FatemehUno, MasatoshiJalil Piran, Md.Uno, ShigeyasuJamalabadi, Mohammad Yaghoub AbdollahzadehUnterlass, MiriamJamali, SadeghUpham, JeremyJames, Peter B.Ur Rehman, MasoodJamrozik, WojciechUras, MarcoJana, AtanuUrbanic, Ruth J.Janas, DawidUrgese, GianvitoJanczyk, GrzegorzUricchio, TiberioJaneliukstis, RimasUrquiza-Aguiar, LuisJang, Byeong-WookUrsu, IoanJang, Gil-JinUrusov, Alexander E.Jang, Ho WonUsamentiaga, RubenJang, HongjeUsery, E.Jang, IngookUshakov, NikolaiJang, JongwookUslar, MathiasJang, ShinaeUsman, MuhammadJang, SoohwanUsov, NikolaiJang, YeongUtrilla-Manso, ManuelJanicki, ArturUtsuzawa, ShinJanicki, GrzegorzV. Achillopoulou, DimitraJankauskas, VytenisVa, VuthaJanko, VitoVacca, AnnalisaJankowski, ŁukaszVacca, GaetanoJankowski-Mihułowicz, PiotrVáclavík, JaroslavJanner, DavideVaculík, JurajJanošek, MichalVaculovičová, MarketaJanota, AlešVagin, MikhailJansen, Carl-PhilippVaglini, GigliolaJansen, K.M.B.Vahtmäe, EleJanting, JakobVaida, CalinJantunen, ErkkiVain, AntsJanusas, GiedriusVaismoradi, MojtabaJaradat, MohammadVaks, VladimirJaren, CarmenValdenegro-Toro, MatiasJarolmasjed, SanazValdez, JoseJaroszewicz, LeszekValdiani, AlirezaJarzabek, DariuszValdivia-Moral, PedroJasielec, Jerzy J.Vale, AlbertoJasinski, GrzegorzVălean, HonoriuJasiul, BartoszValença, JónatasJasiuniene, ElenaValencia, PhilipJat, PrahladValencia-Palomo, GuillermoJato-Espino, DanielValente, AntonioJáuregui-Vázquez, DanielValente, Renata Kelly MendesJavadi, BahmanValenti, Roberto G.Javanmardi, MahdiValenti, Vitor EngraciaJavarone, Marco AlbertoValentini, FedericaJavornik, TomazValentino, GianlucaJavorski Eckert, JonyValenzuela, Jose LuisJawad, Brahim K.Valera, EnriqueJaworek- Korjakowska, JoannaValeriani, DavideJayakody, DushanthaValerio, ApicellaJayaraman, ChandrasekaranValerio, IanJayasumana, SadeepValero, EdelmiraJayavelu, SenthilnathValiente, DavidJean-Fulcrand, AnneliseValigi, Maria CristinaJeannin, Jean-BaptisteValinevicius, AlgimantasJebelli, HoutanValkama, MikkoJechow, AndreasVallati, MauroJedlicka, KarelVallejos, StellaJedlovec, GaryVallet, BrunoJedrzejewska-Szczerska, MałgorzataVallianatos, FilipposJeerapan, ItthiponValous, NektariosJekova, IrenaValsesia, DiegoJelly, ThomasValtierra-Rodriguez, MartinJen, Chun-PingValvano, StefanoJen, Yi-JunVan Bennekom, W.P.Jeng, Jyh-HorngVan Blerkom, DanielJeng, YihVan De Moortele, Pierre-FrançoisJenkins, R. BrianVan Der Eerden, FritsJensen, Brian D.Van Der Loos, HendrikJeon, Heung SeokVan Der Perre, LiesbetJeong, Chang KyuVan Der Velde, EnnoJeong, DongwonVan Der Woerd, H.J.Jeong, Han-YouVan Do, TienJeong, Ho-ChangVan Eerdenburg, FrankJeong, InbaeVan Gastel, MarkJeong, Jin-WooVan Hamme, DavidJeong, JungsikVan Helleputte, NickJeong, SanghwaVan Hoecke, SofieJeong, Seung Heevan Hoof, JoostJeong, UseokVan Hoof, RichardJer Jaw, JenVan Huffel, SabineJerkowić, IgorVan Hulle, MarcJesionek, MarcinVan Laarhoven, TwanJesus, GoncaloVan Laerhoven, KristofJeyakumar, ParamsothyVan Langenhove, LievaJha, NandVan Le, DucJhe, WonhoVan Schyndel, RonJi, HonghaiVan Torre, PatrickJI, KefengVan Vliet, MarijnJi, ShengchangVan Winden, StevenJi, WeiVAN ZWIETEN, Koos JaapJi, XuewuVan, HiepJi, YimingVan, VienJi, ZhanlinVandooren, JenniferJi, ZhengVanetti, Maria Cristina DantasJia, DongyaoVangelista, LorenzoJia, JianfengVanhille, ChristianJia, MaoshenVanhoef, MathyJia, MingmingVanlanduit, SteveJia, QuanxiVanli, O.ArdaJia, WenyanVannozzi, GiuseppeJia, XiaodongVaradinov, Maria JoséJia, YanweiVaranis, MarcusJia, YuVardasca, RicardoJia, YunfangVarde, AparnaJian, Ming-ShenVarga, PalJian, MuweiVarga, RobertJiang, BoVargas Solar, GenovevaJiang, ChaoVargas-Rodriguez, EverardoJiang, ChuandongVargas-Rosales, CesarJiang, ChunxiaoVarghese, JobinJiang, FanVarpula, AapoJiang, HaoVarshney, AmbujJiang, HongchuanVaščák, JánJiang, HuaweiVasconcelos, Flavio HenriqueJiang, Jehn-RueyVashishtha, ParthJiang, JiajiaVasilakos, AthanasiosJiang, JunVasilakos, ChristosJiang, JunlinVasilateanu, AndreiJiang, LelunVasilescu, AlinaJiang, LinVasiliev, AlexeyJiang, NiVasques, GustavoJiang, PingVásquez-Correa, Juan CamiloJiang, QiVassallo, Raquel FrizeraJiang, QiuVassilakis, CostasJiang, RubinVatavu, AndreiJiang, RuinianVauche, RemyJiang, SongVaughan, NeilJiang, WanshouVauhkonen, MarkoJiang, WeikangVavilov, VladimirJiang, WenchaoVázquez, Andrés S.Jiang, XiantaVazquez, FranciscoJiang, XiaoguangVazquez, Rebeca MartinezJiang, XiaoliVázquez-Castillo, JavierJiang, XiqianVázquez-Jiménez, RenéJiang, XuchuanVeeran Ponnuvelu, DineshJiang, XueVeeraraghavan, Ashokjiang, yizhangVega-Nieva, DanielJiang, YongyaoVeganzones, Miguel A.Jiang, YuVegas, JesúsJiang, ZhiyuVehí, JosepJianyu, YangVehkaoja, AnttiJiao, ChaoqunVelázquez, RamiroJiao, JingpinVelazquez-Perez, Jesus EJiao, LichengVelha, PhilippeJiao, PengchengVelichko, SergeyJiao, WanguoVelkov, TonyJiao, YuboVella, FilippoJiao, ZitiVelmuzhov, AlexanderJijie, RoxanaVelotta, RaffaeleJimenez Alonso, Javier FernandoVelten, AndreasJimenez Angeles, LuisVeltink, PeterJiménez, Alfredo ArcosVenafra, SaraJiménez, AntonioVenčkauskas, AlgimantasJiménez, Antonio R.Vendik, IRINAJimenez, CeciliaVendittozzi, CristianJiménez, FelipeVenediktov, VladimirJiménez, FernandoVenkatasubramanian, KrishnaJimenez, RicardoVenkatesh, A.G.Jimenez-Ballesta, RaimundoVentouras, Erricos-ChaimJiménez-Herranz, José MiguelVenturini, FrancescaJiménez-Muñoz, Juan CarlosVenu, AnnamdasJiménez-Muñoz, Juan-CarlosVera, EstebanJimeno-Morenilla, AntonioVera-Pérez, JoseJin, BoVerbelen, TimJin, ChengluVerbovšek, TimotejJin, GeVerbruggen, StefaanJin, HaimingVerd, JaumeJin, JingVerde, FrancescoJin, LishengVerdini, FedericaJin, MiaoVerdun, JéromeJin, Ren-ChengVeres, SandorJin, RuiVereshchaka, Alexey A.Jin, ShuanggenVeres-Székely, AnnaJin, TianVergara, DiegoJin, XiVergari, ClaudioJin, XiaobinVergauwen, MaartenJin, Xue-boVergos, GeorgeJin, ZhanpengVerhaert, NicolasJin, ZhigangVerhaevert, JoJin, ZongwenVerhulst, TobiasJing, JuliaVeríssimo, MartaJing, LeiVerly, JacquesJing, QingshenVerona, EnricoJing, TaoVerotti, MatteoJiralerspong, TrongmunVershovskii, AntonJirka, SimonVértesy, GáborJmerik, ValentinVervisch-Picois, AlexandreJo, Byung WanVerwaeren, JanJo, HongkiVeryaskin, AlexeyJo, KichunVescio, BasilioJoão Ramalhosa, MariaVespignani, MassimoJochymczyk-Woźniak, KatarzynaVespini, VeronicaJofre Roca, LluísVessio, GennaroJohansson, ChristerVestergaard, Mun'delanji C.Johari, PedramVettore, AntonioJohn, VijuVezzani, RobertoJohnson, AnjuVezzetti, EnricoJohnson, Brandy J.Via, BrianJohnson, ColinViáfara, Cristian C.Johnson, EricViassone, MilenaJohnston, MatthewVicent, José F.Jokisch, OliverVicente, Carlos Miguel SantosJolly, PawanVicente, Miguel A.Jombo, GbanaibolouViciani, SilviaJonah, OlusegunVictores, Juan G.Jonak, JózefVidal, IvánJonas, StephanVidal-Álvarez, GabrielJones, Corinne A.Vidal-Verdú, FernandoJones, RyanVidic, JasminaJones, TrevorVidor, Fábio F.Jong, Gwo-jiaViedma, OlgaJonsson, BrorViegas, Eduardo KuglerJonuscheit, JoachimViegas, VítorJoo, Ki-NamVieira Lopes, NunoJoo-Nagata, JorgeVieira, Alex BorgesJoordens, MatthewVieira, JoséJordi, FonollosaVieira, Marcelo BernardesJörg, BeckmannVien, Quoc-TuanJorge, HiginioViespe, CristianJorge, Vitor A. M.Viik, JariJornet, JosepVijay, SaurabhJose, Roberto SanVikas, VisheshJoseph, JamesVilà, AnnaJOSHI, NIRAVKUMARVilanova, RamonJou, ShyankayVilanova, XavierJoubert, TrudiVilardy, Juan M.Joutsijoki, HenryVilaseca Ricart, MeritxellJovanović, SlavišaVilcu, Gabriel EduardJoyce, Thomas J.Vilela, CarlaJózefczak, ArkadiuszVilladangos, JesusJu, XiangyangVilladangos, José ManuelJuan, Carlos G.Villagra, JorgeJuang, Jyh-ChingVillagra, VictorJuárez-Gracia, Antonio-GustavoVillani, Maria GabriellaJudd, Johnn P.Villanueva Vásquez, DanielJudd, MartinVillanueva, Manuel VargasJugessur, AjuVillar, José R.Juidías, Emilio RamírezVillas-Boas, Paulino R.Julien, Le KernecVillegas-Cortéz, JuanJulrat, SakolVilleneuve-Faure, ChristinaJun, KondohVillez, KrisJun, Martin Byung GukVilliger, MartinJun, SunghaeVimarlund, VivianJung, Byung C.Vinayak, ElangovanJung, DaewoongVincent, DidierJung, HaejoonVincent, ThomasJung, HaminVincent, TimothyJung, Jae-HunVincenzo, RomanoJung, JasonVinogradov, EvgeniiJung, MinchaeVinogradov, SergeyJung, SeungwonVinogradov, Sergey L.Jung, Soon KiVinueza Naranjo, Paola GabrielaJung, Woo-SungViola, DonnaJung, WooyoungViolakis, GeorgiosJung, Young DoViollet, StéphaneJungeblut, ThorstenVirdee, BalJunior, Joao Gari Da Silva FonsecaVirdis, AntonioJunior, PedroVirkki, JohannaJunttila, VirpiViroli, MirkoJuránek, RomanVirtanen, Timo H.Jurcevic, MarkoVirzonis, DariusJusas, VaciusVischer, AnninaJust, MichaelVisconti, PaoloJuszczak, RadoslawVisentin, FrancescoJuvekar, ChiraagVishwakarma, Bramha DuttJwo, Dah-JingVitali, Rachel V.Jyothi, VinayakaVitek, StanislavKa, Min-HoViter, RomanKaabouch, NaimaVittoria, BruniKaartinen, HarriVivas, Oscar AndresKabir, AbuzarVivet, DamienKabir, K. M. MohibulViviani, MarcoKabir, MinooVizza, PatriziaKaboli, MohsenVlachogiannis, DiamandoKaburlasos, Vassilis G.Vlachos, EvgeniosKacaniová, MiroslavaVladareanu, LuigeKačániová, MiroslavaVladeanu, CalinKacem, NajibVladyko, AndreiKacem, ThabetVladymyrov, MykhailoKacik, DanielVlahu-Gjorgievska, ElenaKačmařík, MichalVlaj, DamjanKaczmarek, Adam L.Vochin, MariusKaczmarek, MariuszVoglhuber, ThomasKaczmarek, MichałVoglhuber-Brunnmaier, ThomasKaddoum, GeorgesVogt, Dominik WalterKafal, MoussaVohnsen, BrianKafali, OzgurVoiculescu, IoanaKaftandjian-Doudet, ValérieVoirin, GuyKagawa, KeiichiroVojisavljevic, VukKahaki, Seyed M. M.Vojtisek-Lom, MichalKahrizi, MojtabaVolkov, ValentynKaimaris, DimitrisVolmer, MariusKaiser, CarlVolná, EvaKaiser, SusannaVolosencu, ConstantinKaisti, MattiVolpati, DiogoKaivosoja, JereVolpp, JoergKaiwartya, OmprakashVon Wangenheim, AldoKajitani, TakashiVos, StevenKajiwara, YusukeVoß, StefanKaklauskas, ArturasVoué, MichelKalaji, Hazem MohamedVoutetaki, Maria–StylianiKalalas, CharalamposVovides, YiannaKalantar-Zadeh, KouroshVozel, BenoitKalatehjari, RoohollahVoznak, MiroslavKalayci, Tahir EmreVrabec, TinaKalimeri, KyriakiVrančić, DamirKaliniewicz, ZdzisławVrba, JanKaliszewski, MironVrček, NevenKallen, VictorVrigneau, BaptisteKalliatakis, GrigoriosVrochidis, StefanosKalliokoski, MattiVryonides, PhotosKalloniatis, ChristosVrysis, LazarosKalogiros, JohnVu, Ngoc SonKaloxylos, AlexandrosVu, Quang-DoanhKaltiokallio, OssiVujaklija, IvanKalvøy, HåvardVujović, IgorKalyvas, NektariosVukovic, NatashaKamaruzaman, JusoffVuksanovic, BranislavKamat, Vineet R.Vulic, MilivojKamath, RahulVulpe, AlexanduKambezidis, Harry D.Vural, MertKambiz, TehraniVyas, AginKamenar, ErvinVyšata, OldřichKa-Meng, LeiVyskočil, VlastimilKameoka, JunW.k., FungKamezaki, MitsuhiroWada, TomohisaKamienski, CarlosWada, YujiKamienski, Carlos AlbertoWagner, BernardoKamilaris, AndreasWagner, ChristianKamińska, AgnieszkaWagner, IsabelKaminska, AnielaWagner, JörgKaminska, BozenaWagner, NormanKampa, AdrianWagner, PatrickKampel, MartinWagner, ThorstenKampel, SilvanaWagter, Christophe DeKamshilin, AlexeiWahaia, FaustinoKanakaris, VenetisWahde, MattiasKanamori, RyoWahid, Khan A.Kanarachos, StratisWahlström, JohanKandammathe Valiyaveedu, SreekanthWaine, Toby WKandris, DionisisWajman, RadosławKanellakis, ChristoforosWakayama, ToshitakaKanellopoulos, DimitrisWakeman, ShawneeKaneta, TakashiWalas, KrzysztofKang, Bo-YeongWaldman, ChristophKang, ChanKyuWalendziuk, WojciechKang, ChulhunWales, DominicKang, FeiWalewyns, ThomasKang, Gu EonWalker, JacquelineKang, HyunchulWalker, JasonKang, JamesWallace, Carlington W.Kang, JeeunWallace, JonKang, JidongWallace, Joshua S.Kang, JiyeonWallace, VincentKang, Lae-HyongWalsh, KerryKang, LeiWalter, SteffenKang, Li-WeiWalter, ToddKang, Moon GiWalther, AndreiKang, MoonsooWalther, FrankKang, NingWan, JiaKang, Soon JuWan, QuanKang, Suk juWan, WenhuiKang, SungweonWandowski, TomaszKang, TaejoonWang, AiningKang, WenjingWang, BangKang, Yang JunWang, ChanghongKang, YanghuiWang, ChaoKangas, JariWang, ChenKangas, MichaelWang, ChenghaoKangasluoma, JuhaWang, Cheng-LiangKangin, DmitryWang, ChenxingKaniewski, PiotrWang, Chih-HungKanno, AtsushiWang, Chih-YuKano, ShinyaWang, Chiu-YenKansanen, KimmoWang, Chuyuan (Carter)Kant, KrishnaWang, CrystalKantak, ChaitanyaWang, DaleiKantarci, BurakWang, DanlingKantaros, YiannisWang, DepengKapalavavi, BrahmamWang, DianhuiKaplan, AndreyWang, DingKapoulas, VaggelisWang, DongKapuscinski, TomaszWang, DongmingKara De Maeijer, PatriciaWang, FeiKarabasov, SergeyWang, Fu-KangKarabinis, Athanasios I.Wang, Fu-KwunKaragianni, EvangeliaWang, FupengKarahalios, HristosWang, GuangjianKarakuş, OktayWang, GuijinKarami, AminWang, GuodongKara-Mohamed, MohamedWang, GuoquanKarantzalos, KonstantinosWang, HaiKarathanassi, Prof. V.Wang, HaigangKaratsidis, AngelosWang, HaihongKaratzas, KostasWang, HaiyingKarayannis, ChrisWang, HanliKarg, PatriciaWang, HaoKargas, GeorgeWang, Harry HaoxiangKargas, GeorgiosWang, HengKarim, LutfulWang, He-shegKarim, NazmulWang, HongKarim, NissarWang, HongquanKarimi, HassanWang, HongwuKarimov, TimurWang, HuanKarkanis, AnestisWang, HuanqingKarkanis, Stavros A.Wang, HuaqingKarkazis, Panagiotis A.Wang, HuibinKarki, SitaWang, Jia-JungKarlik, BekirWang, JiananKarlsson, PetraWang, JiangtaoKarnaushenko, DaniilWang, Jianguo JackKärnfelt, CamillaWang, JianqiKarnowski, KarolWang, JianqiangKaroń, GrzegorzWang, JiaoKarpenko, OleksiiWang, JiaoleKarperien, Audrey L.Wang, JiaweiKarpov, DmitryWang, JidongKarpov, SergeyWang, JinKaruppannan, Senthil KumarWang, JinghuiKarutaa Gnaniar, Sampath KumarWang, JinhuKaryakin, ArkadyWang, Jin-YuanKaryotis, VasileiosWang, JunKasahara, ShojiWang, JungangKasai, NaoyaWang, JunshengKasapakis, VlasiosWang, JunyuanKashaninejad, NavidWang, KaiKashif, MuhammadWang, Kai-ChengKasneci, EnkelejdaWang, KanKasprowski, PawełWang, KangKasprzak, WłodzimierzWang, KeKassal, PetarWang, KeshengKassanos, PanagiotisWang, KezeKassaras, IoannisWang, Kun-ChingKasser, MichelWang, Kuo-nungKästner, ChristianWang, LeiKästner, MarkusWang, LiangminKastner, WolfgangWang, LidaiKataoka, HirokatsuWang, LifengKataoka, YusukeWang, LimingKatapodis, PetrosWang, LinKateris, DimitriosWang, LingyunKathayat, Rahul SinghWang, LuluKathuria, HimanshuWang, MengKati, AnttilaWang, Michelle (Yibing)Katkovnik, VladimirWang, MingshuKatoh, RyuziWang, Ming-ShyanKatona, JozsefWang, MingsongKatsanos, ChristosWang, MinjuanKatsaros, KonstantinosWang, Mu-chunKatsikouli, PanagiotaWang, MuguangKatsikouli, Panagiota (Yota)Wang, NengchaoKatsoulas, NikolaosWang, NingKatz, Brian FGWang, PeihongKaur, AmardeepWang, PengKaushik, AjeetWang, PengfeiKavakiotis, IoannisWang, PichaoKavitha, G.Wang, Pi-ChungKawahito, ShojiWang, PoKawala-Sterniuk, AleksandraWang, QiKawalec, AdamWang, Qian JaniceKawamoto, YuichiWang, Qi'angKawamura, KensukeWang, QiaohuaKaya, SavasWang, QiaosongKayacan, ErkanWang, QingKayes, ASMWang, QingQingKaynak, AkifWang, QiuguKazantsev, DaniilWang, QiuhuaKazanzides, PeterWang, RongKazimierczuk, KrzysztofWang, RuiKazimierski, WitoldWang, ShangKazuhiro, KondoWang, ShangguangKe, YinghaiWang, ShaohuaKealy, AllisonWang, ShapengKearney, PaulWang, Shau-ChunKeating, AdrianWang, ShengkaiKee, ChangdonWang, ShengqiangKee, ScholtenWang, Sheng-ShihKee, Seong-HoonWang, ShibingKefauver, Shawn C.Wang, ShiweiKehl, FlorianWang, TaoKelemen, MichalWang, TengKeller, DustinWang, TianKellnhofer, PetrWang, TianshiKellogg, RyanWang, WeiKelner, JanWang, WeizhuoKelty-Stephen, Damian GWang, WenKemna, StephanieWang, WenguangKemper, BjörnWang, WentaoKenett, Ron S.Wang, WilliamKennedy, DavidWang, XianboKennedy, RoyWang, XiandiKeogh, PatrickWang, XiaogongKepak, StanislavWang, XiaohongKépeš, ErikWang, XiaoyangKeramaris, EvangelosWang, XiaoyuKeramidas, GeorgiosWang, XiaoyuanKerman, KaganWang, XilinKermarrec, GaelWang, XingyuanKern, StefanWang, XinQingKerr, EmmettWang, XinyuanKersten, TobiasWang, XishaKertesz, AttilaWang, XuanKeser, TomislavWang, XuantongKeskinarkaus, AnjaWang, XueKeski-Saari, SaritaWang, XuefengKeszöcze, OliverWang, XuejuKhaleghian, MeysamWang, XueweiKhaleghi-Meybodi, MortezaWang, XuewenKhalid, AtaWang, XuezhiKhaliel, MaherWang, YaKhaliq, Dr JibranWang, YanKhamesee, BehradWang, YanfengKhamis, HebaWang, YangKhamis, MohamedWang, YanshanKhan, AamirWang, YanyanKhan, Adil MehmoodWang, YawenKhan, ArshiaWang, YeqiaoKhan, AsiyaWang, YiKhan, DiganganaWang, Yibing M.Khan, Dr. FaheemWang, YideKhan, FaheemWang, YifanKhan, JesminWang, YingKhan, Md. Rajibur RahamanWang, YipingKhan, Mohammad A.Wang, YishouKhan, MuhammadWang, YizhongKhan, Muhammad ShujaWang, YongcaiKhan, PervezWang, YonggangKhan, SaadWang, YongxiangKhan, SamirWang, YuKhan, SulemanWang, Yuan-KaiKhan, TareqWang, YueKhan, Umar F.Wang, YuehangKhan, UsmanWang, YufengKhandaker, MorshedWang, YuhuiKhansur, Neamul HayetWang, Yu-KaiKhanzadeh, MojtabaWang, Yu-LinKhare, HarmanWang, YunKharel, RupakWang, YuncaiKhateb, FabianWang, Yun-MingKhatibi, Akbar A.Wang, YunshengKhatir, SamirWang, Yu-TeKhattab, TawfikWang, ZhanKhazaei, BahramWang, ZhanyongKhedr, MaanWang, ZhelongKhenchaf, AliWang, ZhengfangKhodabakhsh, AmirWang, ZhengqiangKhodadadi, MohammadWang, ZhenxinKholodnyak, DmitryWang, ZhiKhonina, SvetlanaWang, ZhiguangKhonina, Svetlana N.Wang, ZhipingKhorov, EvgenyWang, ZhongliKhosravan, NajiWang, ZuankaiKhoury, HiamWarisawa, Shin'ichiKhurshid, ZohaibWarnick, Karl F.Kia, Seyed MostafaWarren-Smith, StephenKianfar, JalilWąs, JarosławKiang, Jean-FuWashizawa, YoshikazuKianoush, SanazWasisto, Hutomo S.Kiat, John EmmanuelWatanabe, FernandaKibria, Mirza GolamWatanabe, KeigoKickmeier-Rust, MichaelWatanabe, TetsuyouKiedrowski, PiotrWatanobe, YutakaKieninger, JochenWatier, BrunoKierat, WojciechWatkins, A. NealKiersztyn, AdamWatson, DennisKijanka, PiotrWatson, DesKikuchi, HiroakiWatts, SimonKilikevičius, ArtūrasWawrzyniak, NataliaKillgore, JasonWażny, MariuszKim, AlbertWeaver, Vincent M.Kim, BongjuWeber, AndreasKim, Byung-GyuWeber, FelixKim, ChaekyuWeber, Gerhard WilhelmKim, ChangjaeWeber, KonradinKim, Chang-SeiWeber, RobertKim, ChealWeber, ThomasKim, ChesoongWebster, BerenikaKim, ChulhongWebster, JohnKim, DaeEunWęglarski, MariuszKim, DaeheeWehrmeister, MarcoKim, DeokhwaWei, BaishenKim, Do HwanWei, BinKim, DonghoonWei, Chun-ShuKim, Dong-SeongWei, Chun-YiKim, Du YongWei, Da-HuaKim, EunhaWei, DaijunKim, EunwooWei, DongyanKim, Gi-WooWei, FengKim, GunWei, GuangfeiKim, HakilWei, GuohuaKim, HongjinWei, HaoyunKim, Hye-JinWei, HemingKim, HyeokWei, Hua-liangKim, HyogonWei, LiKim, Hyug-HanWei, MenglianKim, HyunWei, QingyangKim, Hyun SungWei, ShanshanKim, HyunbumWei, WeiKim, Hyung SeokWei, XiaohuiKim, HyungminWei, XiukunKim, Hyung-SinWei, ZhenboKim, HyunseokWei, ZhiQiangKim, HyunsungWei, ZhishunKim, InhiWei, ZhongbaoKim, InsooWeidinger, TamasKim, Jae JoonWeidinger, TamásKim, Jae KyeongWeidner, RobertKim, JayoungWeigel, RobertKim, Jee-InWei-Kocsis, JinKim, JeonghyunWeinberg, MarcKim, JeongraeWeinstein, BenKim, Jeong-TaeWeintrit, AdamKim, Ji-HwanWeisong, LiuKim, Jin YoungWeiss, Sharon M.Kim, JingookWelch, MitchellKim, JinhoWeldehawaryat, Goitom KahsayKim, JinwookWeltin, AndreasKim, JinyoungWen, ChangbaoKim, Jo-ChunWen, Chih-YuKim, Jong-hoonWen, ChihyungKim, Joo-HyungWen, FeiKim, Jung Tae(Steve)Wen, GuanghuiKim, JungkwunWen, KunhuaKim, JungyoonWen, MiaowenKim, Jun-SuWen, Tzi-HungKim, Kap JinWen, YangmaoKim, KeonwookWen, ZhenKim, Ki IlWen, ZhenyuKim, Ki-BokWendel, JanKim, Ki-IlWeng, Ching FengKim, KukjooWeng, ShunKim, Kyong HonWeng, WeiKim, Kyung-SookWenya, WUKim, Mi JeongWeppner, JensKim, Min YoungWeres, JerzyKim, MinKooWerman, MichaelKim, Moon IlWerneck, MarceloKim, MoonseongWerner, Carl FrederikKim, NamWerner, ChristianKim, Nam-YoungWerner, SebastianKim, RobinWesolowski, KrzysztofKim, Robin EunjuWestberg, JonasKim, Sang MoonWestbrook, PaulKim, SangdongWesterdahl, DaneKim, SangkilWesterlund, TomiKim, SangtaeWestern, DavidKim, Seong JinWeyn, MaartenKim, Seong-EunWeyrich, MichaelKim, SeunghyunWhalley, JacquelineKim, ShihoWhang, MincheolKim, SoaramWheeler, NatalieKim, SooyoungWicks, MichaelKim, Sung JaeWidhalm, PeterKim, SunghoWieczorek, Piotr Z.Kim, SunwookWiegerink, RemcoKim, SvetlanaWiegner, MatthiasKim, Tae-HyungWielgosz, MaciejKim, Tae-JoonWieringa, FokkoKim, TaewooWierzba, PawełKim, Tai-HoonWierzbicki, DamianKim, TaihoonnWijesinghe, Ruchire ErangaKim, UikyumWijk, Kasper VanKim, WonhaWijnhoven, RobKim, WonjunWild, GrahamKim, YeonginWilkins, RossKim, Yong SikWillander, MagnusKim, Yong SinWillassen, TrygveKim, Yong-GukWilliams, René M.Kim, Yong-GyooWilliams, WesleyKim, Yong-HwaWilliamson, Craig A.Kim, YoungbokWilliamson, ScottKim, Young-DukWillis, IanKim, Young-GabWillis, Katharine S.Kim, YoungjooWilm, JakobKim, YoungokWilmer, ChristopherKim, Young-RokWilmes, AnjaKim, Young-SikWilson, AdrianKimmel, RonWilson, Alphus DKinar, Nicholas J.Wilson, Christopher G.Kincses, ZoltánWilson, DanKindt, PhilippWilson, JosephKindu, MengistieWilson, William C.King, ChristineWimmer, MichaelKirchner, Elsa AndreaWine, MichaelKirchner, MatthiasWiniarski, TomaszKirk, AndrewWiniecki, WieslawKirsanov, DmitryWinkler, AndreasKirste, ThomasWinkler, Joab R.Kisala, PiotrWinkler, KKiselev, AndreyWinkler, StefanKishimoto, SatoshiWinkler, Thomas E.Kissinger, ThomasWiora, JózefKitamura, AkiraWiśniewski, RemigiuszKitano, ClaudioWiśniowski, PiotrKitsos, ParisWitayangkurn, ApichonKlaina, HichamWitczak, MarcinKlantsataya, ElizavetaWitczak, PiotrKlapez, MartinWitkowska Nery, EmiliaKlasnja-Milicevic, AleksandraWitos, MiroslawKlein, ItzikWitte, HartmutKlein, LeventeWitte, Hartmut F.Kleinschmidt, João HenriqueWITZ, Jean-FrançoisKlem, KarelWixted, AndrewKlepka, AndrzejWlodarczyk-Sielicka, MartaKliks, AdrianWodarski, PiotrKlimek, RadosławWoerd, Hendrik Jan Van DerKlímová, BlankaWohlrabe, KlausKlingler, FlorianWojasiński, MichałKljun, NataschaWojciech, GiernackiKljusurić, Jasenka GajdošWojciechowski, AdamKlomp, MatthijsWójcik, KrzysztofKłosowski, GrzegorzWójcikowski, MarekKłosowski, ŁukaszWojkiewicz, Jean-LucKlouda, LedaWojtas, JacekKlueh, UlrikeWójtowicz, PatrykKluge, TilmannWolf, Robert C.Kmoch, AlexanderWolff, MarcusKnaflitz, MarcoWolffenbuttel, ReinoudKnapkiewicz, PawełWolkiewicz, MarcinKnappenberger, ThorstenWollmann, PhilippKnauth, StefanWolny-Kucińska, AdaKneen, Melanie AnneWolszczak, PiotrKnipfer, ChristianWon, Chee SunKnuteson, RobertWon, Joong-SunKnypiński, ŁukaszWon, MyounggyuKo, ByoungChulWong, Chau-WaiKo, Chia-NanWong, Danny K. Y.Ko, ConnieWong, David Shan-HillKo, HaneulWong, KelvinKo, HoonWong, Kin-HongKo, Hyoung HoWong, LawrenceKo, JeongGilWong, Ling TimKo, Kwang HeeWong, Ling-timKobayashi, HidekiWong, Terence T. W.Kobayashi, MakikoWoo, HyenkyunKobayashi, ShunsukeWoo, JinseokKobayashi, YoshikazuWoo, Jong-KwanKobsar, DylanWoo, Seong TakKoch, AlexanderWoo, Wai LokKoch, ManfredWood, Graham S.Koch, MarkusWoodford, BrendonKocian, AlexanderWoodhead, IanKocifaj, MiroslavWoodhouse, JimKocur, DusanWoods, JohnKodikara Arachchi, HemanthaWoods, JoshKodors, SergejsWoolley, ElliotKoehler, BerndWorsley, PeterKogia, MariaWoschitz, HelmutKoh, AhyeonWotzka, DariaKoh, JaehanWouda, Frank Jkoh, Jung HyukWoyessa, GetinetKoh, Kah HowWozniak, MarcinKoh, Seok-JooWozniak, Slawomir B.Koh, Yee RuiWrenger, BurkhardKohler, MonicaWright, Ruishu F.Kohut, PiotrWróżyński, RafałKoike, YasuharuWu, BianKoike, YoshikazuWu, BInKõiva, RistoWu, ChangshanKojima, RyosukeWu, ChenKojima, ShoichiroWu, ChenshengKok, ManonWu, Chun HoKokinou, EleniWU, Chun HoKokkalis, PanagiotisWu, Chung-Tse MichaelKokkinos, ChristosWu, ChunshengKokkinos, PanagiotisWu, ChunxueKokkoniemi, JoonasWu, DapengKokkoris, GeorgeWu, DiKołakowska, AgataWu, DufanKolakowski, JerzyWu, GangKolasiński, PiotrWu, GuoqiangKolb, AndreasWu, HangbinKolios, StavrosWu, Hong RenKolláth, ZoltánWu, HongpengKolosz, BenWu, Huai-NaKolozali, ŞefkiWu, HuamingKoma, ZsofiaWu, I-ChenKómar, LadislavWu, JayneKomarov, MikhailWu, JiafengKomatsu, TeruhisaWu, JiajunKommuri, Suneel KumarWu, JianboKomninos, NikosWu, JianfaKon, FabioWu, JianfengKonar, ArkaprabhaWu, JiankangKoncar, VladanWu, JianpingKondru, NaveenWu, JianqingKong, DemingWu, JunKong, LingfuWu, KaiKong, LingjieWu, LianjunKong, QingzhaoWu, LiliKong, WanzengWu, LinKong, XiangxiongWu, MengxiKono, HitoshiWu, MinKono, YasuyukiWu, NanKonovalov, Dmitry A.Wu, QiKonovalov, SergeyWu, QingqingKonovaltsev, AndriyWu, Sau-HsuanKonstantaki, MariaWu, ShengbiaoKonstantinos, VotisWu, ShiangjenKonstantynovski, KostyantinWu, Shih-JehKontoni, Denise-Penelope N.Wu, ShuicaiKonukseven, Erhan İlhanWu, Shu-PaoKonushin, AntonWu, ShupingKonyakhin, Igor A.Wu, ShuyingKoohbor, BehradWu, TaixiaKoohbor, Behrad BehradWu, TonghuaKopačková-Strnadová, VeronikaWu, WanqingKopechek, JonathanWu, WeiKopecký, DušanWu, Wei-TaoKopel, PavelWu, WengangKopelevich, OlegWu, XiangKoplin, ChristofWu, XiaofengKoptyug, AndreyWu, XiaolingKopyt, PawełWu, XiaoshengKor, Ah-LianWu, XiongbinKorablev, OlegWu, XuejianKoral, CanWu, XueruiKorbel, PiotrWu, YihongKordella, StavroulaWu, Yik-ChungKordestani, MojtabaWu, YuanmingKordos, MiroslawWu, YuanxinKordov, KrasimirWu, ZhengKoren, KlausWu, ZhongKoreň, MilanWu, ZhuKoricic, MarkoWu, ZiyanKorki, MehdiWuming, ZhangKorkiakoski, VisaWunderli, Jean-MarcKorneev, DenisWurz, Marc ChristopherKörner, JuliaWydra, MichalKorobiichuk, IgorWylie, StephenKorodi, AdrianWysocki, MarianKorostynska, OlgaXavier-de-Souza, SamuelKorotcenkov, GhenadiiXenakis, ApostolisKorposh, SerhiyXhafa, FatosKorwan, DanielXi, JuntongKorzun, DmitryXi, PengKos, AntonXi, XugangKos, MarkoXia, ChunleiKos, TomislavXia, ChunmingKosa, GaborXia, FengKosar, TomazXia, HaiyunKosec, TadejaXia, LinyuanKoseki, YoshihikoXia, MinghuaKoslowski, Hans RudolfXia, RenboKosman, JoannaXia, WeiKosmopoulos, DimitriosXian, XiaojunKošnar, KarelXiang, ChaoqunKosnik, DavidXiang, DeliangKosolapov, Alexey F.Xiang, HengxueKost, GeraldXiang, LiangzhongKostelecký, JakubXiang, LinKostin, Vladimir NikolaevichXiang, RongKostopoulos, George K.Xiang, XianboKostrzewa, DanielXiang, YijianKostrzewski, MariuszXiang, YuanjiangKostyuchenko, Evgeny YuryevichXiang, YujiangKotelba, AdrianXiang, YunKothapalli, Sri-Rajasekhar (Raj)Xiang, ZhiyuKottapalli, Ajay Giri PrakashXiao, BingKotuliak, IvanXiao, DeqinKotyński, RafałXiao, DunhuiKou, GangXiao, FuyuanKouchaki, SamanehXiao, GuijianKoucheryavy, AndreyXIAO, GuoruiKoudelková, ZuzanaXiao, JielingKoudouridis, Georgios P.Xiao, KaijunKouh, TaejoonXiao, LiangKoukiou, GeorgiaXiao, LiangliangKoulamas, ChristosXiao, YongKoulouridis, StavrosXiao, YuKourkouli, PenelopeXiao, YuzheKõuts, TarmoXiao, ZhiqiangKoutsandria, GeorgiaXiao, ZhuKoutsia, NikosXiao, ZhuolingKoutsouris, Dimitris DionissiosXiaoying, OuyangKouvelas, AnastasiosXibilia, Maria GabriellaKouzes, RichardXie, HuikaiKouziokas, Georgios N.Xie, JiafengKouznetsov, Rostislav D.Xie, JingKouzoudis, DimitrisXie, JunqiKovač, OndrejXie, KunKovacević, TonkoXie, LeiKovács, ZoltánXie, LiangKovalev, Valeri I.Xie, LipingKovalevsky, Andrei V.Xie, MengjunKovář, JiříXie, MengyingKovari, AttilaXie, ShangranKowal, JuliaXie, ShuangKowal, VirginiaXie, TingKowalczuk, ZdzisławXie, WeiyingKowalczyk, AgataXie, XiufengKowalczyk, KonradXie, YanhuaKowalczyk, SebastianXie, YingyingKowalski, DariuszXie, YuedongKowalski, GregoryXie, YujiangKowalski, LukaszXie, YuliangKowalski, MarcinXie, ZhijianKowerko, DannyXifré-Pérez, ElisabetKoyama, YuyaXin, BaoguiKoydemir, HaticeXin, KunlunKozas, KonstantinosXing, DAKozey Keadle, SarahXing, FeiKozhoridze, GiorgiXing, MengdaoKozik, RafałXiong, BinKoziol, MateuszXiong, GuangmingKozlov, KonstantinXiong, JiaqingKozłowski, EdwardXiong, JieKraft, MarekXiong, JinboKraft, MartinXiong, JipingKraft, ThomasXiong, KaiKrajník, TomášXiong, KeKralevska, KatinaXiong, LinKranz, ChristineXiong, NaixueKrasemann, HajoXiong, XiaogangKrasnok, AlexXiong, YonghuaKrasoulis, AgamemnonXiong, ZhiKrasovsky, TalXu, ChaoKrasteva, VesselaXu, ChengKrasuski, AdamXu, ChenyangKrasuski, KamilXu, ChiKratzke, NaneXu, ChongKraus, HannesXu, DanKrause, Hans-JoachimXu, Dong-ShengKravari, KalliopiXu, FengKravtsov, SergeyXu, GabrielKrebsz, MelindaXu, GuobaoKreimer, JosephXu, GuochangKreislova, KaterinaXu, HaitaoKrejcar, OndrejXu, HeKrejsa, JiříXu, HongliKrell, Mario MichaelXu, HuiKremen, VaclavXu, JianKremlev, ArtemXu, JiangboKrief, FrancineXu, JiangtaoKrishnan, SiddharthXu, JiaqiangKrishnaswamy, BhuvanaXu, JingweiKrishtop, VladimirXu, JuanjuanKristensen, Anders RinggaardXu, JunKristoffersson, AnnicaXu, KaichenKritikakou, AngelikiXu, KaikaiKrivetskiy, ValeriyXu, KailiKrobka, NikolayXu, KeKroeger, SilkeXu, KeleKrólczyk, GrzegorzXu, KunKrólczyk, JolantaXu, LangKrólicka, AgnieszkaXu, LijunKroll, LotharXu, LinfengKronander, JoelXu, LingweiKroupa, MartinXu, MinghuiKrtalić, AndrijaXu, PengchengKruczek, ZygmuntXu, QuanKrueger, EddyXu, RuiKrüger, BjörnXu, ShengKrüger, WolfgangXu, ShengyongKrupitzer, ChristianXu, ShoujunKruss, SebastianXu, TingtingKrygier, JaroslawXu, TongyangKrysinski, PawelXu, TuanweiKrzak, Joseph J.Xu, WeiqingKrzeszowski, TomaszXu, WeitaoKrzykowska, KarolinaXu, WenjunKrzysztof, NausXu, WTKrzysztofik, IzabelaXu, XiangKrzywiecki, ŁukaszXu, XiangminKrzyzak, AdamXu, XiangyangKtena, AphroditeXu, XiaohuaKu, Chia-HaoXu, XiaojiKu, Nian-Wei (Tony)Xu, XiaolanKuai, JieXu, XiaoLiKuan, Yean-DerXu, XiaominKuang, YangXu, XiaoweiKubiak-Wójcicka, KatarzynaXu, XunKubicek, JanXu, YanyanKubíček, VojtěchXu, YingKubik, ZdenekXu, YongKubota, KeiichiXu, YongjunKucera, MartinXu, YongmingKuckling, DirkXu, YougenKucner, TomaszXu, YufuKudela, PawelXu, YuquanKudela, PawełXu, YushengKudelski, AndrzejXu, ZheKujawa, SebastianXu, ZhihuoKujawinska, MalgorzataXu, ZhongyangKujawska, MałgorzataXuan, QiKukaev, AlexanderXuan, ShouhuKula, SławomirXue, BindangKuladinithi, KoojanaXue, CunjinKulawiak, MarcinXue, JinlinKulesza, WlodekXue, TianyuKulesza, ZbigniewXue, XinyuKulha, PavelXue-Feng, HeKulhandjian, HovannesXuerui, GaoKulich, MiroslavXW, WangKulinich, SergeiXylomenos, GeorgeKulkarni, AnandYaacoubi, SlahKulpa, KrzysztofYabe, IchiroKulyukin, VladimirYabuki, MasanoriKumar And Roy Choudhury, Piyush And AhanaYaghouby, FaridKUMAR YADAV, SUSHEELYaguchi, YuichiKumar, AbhishekYahiaoui, RedaKumar, AmitYalikun, YaxiaerKumar, AnilYamada, HidekiKumar, GaneshYamada, IchiroKumar, ManojYamada, SatoruKumar, ManuYamagishi, KentoKumar, NareshYamagiwa, ShinichiKumar, PankajYamaguchi, TomoyukiKumar, PardeepYamaguchi, YukiKumar, PradeepYamanaka, MasahitoKumar, SajeeshYamane, SatoshiKumar, ShailabhYamaner, YalcinKumar, Suryadevara NagenderYamashita, HirofumiKumar, VineetYamauchi, TakashiKumar, VivekYamazaki, KimitoshiKume, TomonoriYamazoe, HirotakeKumhala, FrantišekYan, JiaKunapuli, GautamYan, JingKuncová, GabrielaYan, JiningKundracik, FrantisekYan, JunkunKundu, AbhishekYan, KeKundu, ChinmoyYan, LeiKundu, TribikramYan, MingyuanKunert, WolfgangYan, QiaoKunicki, MichałYan, SenKunihisa, TashiroYan, YongKunkel, JulianYan, ZhepingKunpeng, FengYan, ZhongjiangKunugi, TomoakiYáñez-Sedeño, PalomaKuo, Chao-LinYang, BaijianKuo, Chung-HsienYang, ChangxiKuo, Hsing-FuYang, ChaoweiKuo, Tung-WeiYang, ChengKuok, Sin ChiYang, ChenguangKuok, Sin-ChiYang, Chia-MinKurabayashi, DaisukeYang, Chih-YuKurachi, RyoYang, ChunhuaKurata, NaritoYang, DanKurata, TakeshiYang, DiangeKurata, YoheiYang, DongkaiKurazume, RyoYang, FangKurbatov, A.M.Yang, FeiKuriakose, MajuYang, FengKurita, KazunariYang, GangqiangKurlyandskaya, Galina V.Yang, GuanciKurtser, PolinaYang, GuangKurugollu, FatihYang, GuominKurz, MarcYang, HaifengKUSAMA, YusukeYang, HangKusari, ArpanYang, HongzhouKusek, MarioYang, Hsiao-YuKushnir, Sergey E.Yang, HuiKushwah, VarunYang, JaewonKussul, NataliiaYang, JasonKusters, IljaYang, JiachenKutafina, EkaterinaYang, JiamiaoKutin, JožeYang, JianhuaKutt, KrzysztofYang, JianjunKuusik, AlarYang, JiaqiKuželka, KarelYang, JieKuzilek, JakubYang, JingKuznetsov, SergeyYang, Jong-RyulKuzovkin, DmitryYang, KailunKvas, AndreasYang, KaiyuanKwan, ChimanYang, LiangjingKwan, PatrickYang, LianxiangKwasnicki, Richard MarkYang, LinghuiKwok, Sheldon J.J.Yang, MijiaKwon, Dong-SooYang, MinhaoKwon, JinhyeongYang, QuanlongKwon, JunseokYang, RuiKwon, MinhaeYang, ShanKwon, Soon-HongYang, Shanchieh JayKwon, SoonilYang, ShichunKwon, Soon-kakYang, ShimingKwon, SoonwookYang, ShufanKwon, TaehoonYang, ShuhuiKwon, TaekyoungYang, ShumingKwon, YounggooYang, Su ScottKyamakya, KyandoghereYang, TaoKyas, MarcelYang, TingbinKyrarini, MariaYang, Tsung-HsunKyriakopoulos, KonstantinosYang, WeiKyveryga, PeterYang, WeiqingLa Belle, JeffreyYang, WenLa Notte, LucaYang, WenchengLa Russa, MauroYang, WendongLa Spada, LuigiYang, WuqiangLa Torraca, PaoloYang, XiaodongLa, Duc DuongYang, XinghuaLa, HungYang, XinmaiLa, Thanh-GiangYang, XuLaaksonen, HannuYang, XueLabardi, MassimilianoYang, YangLabonte, GillesYang, YansongLabra Gayo, José EmilioYang, Yi (Tsinghua University)Labuda, JanYang, Yi (Wuhan University)Labus, AleksandraYang, Yuan-SenLabuta, JanYang, Yu-MingLacaze, BernardYang, YunjieLachapelle, GerardYang, YunzeLacidogna, GiuseppeYang, YushiLacik, JaroslavYang, ZhaochuLackovic, IgorYang, ZhaohuiLacy, StuartYang, ZhenchuanLadha, CassimYang, ZhiboLaefer, DebraYang, ZhileLaerhoven, Kristof VanYaniv, ZivLaffly, DominiqueYannis, GeorgeLaflamme, SimonYano, MitsuakiLaforge, François O.Yanushkevich, Svetlana N.Lage, EnnoYao, Da-JengLaghrouche, MouradYao, DezhongLagkas, ThomasYao, FanLago, PaulaYao, HaipengŁagód, GrzegorzYao, JianLaguarda-Miró, NicolásYao, JunLahav, AssafYao, LanLahmer, TomYao, LeehterLahmiri, SalimYaqoob, IbrarLai, Chao-SungYaqub, Muhammad AzfarLai, ChinLunYardimci, Nezih TolgaLai, JianbinYarovoy, AlexanderLai, JinxingYarushkina, NadezhdaLai, JizhouYasun, EmirLai, PuxiangYasyukevich, YuryLai, StefanoYatsu, YoichiLai, Wen ChengYavari, EhsanLai, Ying-ChihYazdanpanah, HamedLai, YingxuYe, ChaofengLai, Yu-ChiYe, JiaMinLain, Jenn-KaieYe, NanLal, ChhaganYe, RuquanLallana, Pedro C.Ye, WeilinLallas, Efthymios N.Ye, XuesongLallechere, SébastienYe, YuLalos, ArisYeh, Chia-HunLam, MaryYeh, Chia-HungLam, Poh-SangYeh, Chien-HungLam, Soi HoiYeh, Chih-FengLam, Wei HaurYeh, Lo-YaoLamberti, FabrizioYeh, Min-HsinLambert-Torres, GermanoYeh, Sheng ChengLambri, Osvaldo A.F.Yeh, Sheng LihLameiro, ChristianYeh, Wei-ChangLamonaca, FrancescoYeh, Yin-TingLamparelli, Rubens Augusto CamargoYehezkeli, OmerLan, GuohaoYekan, TahaLan, ShengchangYelamarthi, KumarLan, ShoufengYen, Jia-YushLan, YuchengYen, Yi-KuangLancaster, DavidYeo, Joo ChuanLanceros-mendez, SenentxuYeo, JunhoLanders, AndrewYeo, Woon-HongLandowska, AgnieszkaYeom, EunseopLane, BrandonYeom, JunghoonLane, DavidYeom, SeokwonLaneve, GiovanniYesilkoy, FilizLang, Hans PeterYeum, Chul MinLang, JochenYi, DaqingLang, MichaelYi, JianxinLang, WalterYi, JingangLange, HolgerYi, PengxingLange, ManfredYi, TinghuaLangelaan, Jack W.Yi, WeiLangendoerfer, PeterYi, Won-JaeLangley, PhilipYi, ZaoLangone, RoccoYi, ZhenxiangLanillos, PabloYiannopoulos, KostasLanotte, RuggeroYihun, YimeskerLanovaz, JoelYildirim, Kasim SinanLantada, NievesYildiz, Huseyin UgurLantsoght, Eva O.L.Yilmaz, MehmetLanza, GiselaYin, GaofeiLanza, GiuseppeYin, JunjunLanza, JorgeYin, KunboLanza, Luca GiovanniYin, ShenLanzolla, AnnieYin, TaoLao, Keng WengYin, WuliangLaparra-Hernández, JoseYin, XiaobinLapington, JonYin, XiaokangLappalainen, KariYin, XiaoluLara Molina, Fabian AndresYin, ZhendongLarsen, YngvarYin, ZunLarsson, MarcusYing, JieLarue, GrégoireYitmen, IbrahimLashkari, BahmanYizhuo, ZhangLaskowski, ŁukaszYobanny Reyes López, SimónLasky, TyYokota, TatsuyaLassila, AnttiYokota, TomoyukiLastra, MiguelYokoyama, TakashiLatif, TahmidYoneda, KeisukeLatorrata, SaverioYonel, BariscanLatorre, PedroYong, Kar WeyLatorre-Carmona, PedroYoo, ByungseokLatré, StevenYoo, ChuckLatychevskaia, TatianaYoo, JaehyunLaude, VincentYoo, SeehwanLauneau, PatrickYoon, BoraLaurendeau, DenisYoon, ChangwooLaurent, JérômeYoon, HeenamLaureti, StefanoYoon, HyosangLaurini, RobertYoon, HyungchulLauritano, DorinaYoon, IkjuneLautner, GergelyYoon, Ji-WookLavagetto, FabioYoon, JongheeLavalliere, MartinYoon, Moon-YoungLavine, BarryYoon, SeokhoonLavric, AlexandruYoon, ZizungLawnik, MarcinYordanova, KristinaLawrence, DaleYoshino, ToshihikoLawrence, KarlYoshinobu, TatsuoLawrence, PeterYoshizawa, ShingoLay-Ekuakille, AiméYou, BaiqiangLazár, DušanYou, ChangshengLazar, ZsofiaYou, Cheol-HwanLazarescu, MihaiYou, Hye JinLazaridis, PavlosYou, JiseonLázaro, AntonioYou, TianyanLazaro, JesusYou, XingeLázaro, JoséYoun, Hee YongLazarov, Andon DimitrovYoun, Ik-HyunLazarus, NathanYoun, InchanLazecky, MilanYounes, RabihLazik, DetlefYoung, Kuu-YoungLazkano, ElenaYoung, RupertŁazuga, KingaYoung, Sheng-JoueLe Bars, FabriceYounus, Muhammad UsmanLe Bas, Pierre-YvesYousaf, JawadLe Borgne, BriceYousefi, BardiaLe Dimet, François XavierYousif, Jabar H.Le Faucheur, AlexisYu, AnxiLe Grognec, ErwanYu, BailangLe Moal, PatriceYu, ChenLe, Duc VanYu, Cheng-JuLe, NamTuanYu, Chin-pingLe, The DungYu, ChunyangLe, TrungYu, Deng-GuangLe, Trung-KienYu, FabiaoLe, ZhangYu, HaitaoLeal Junior, ArnaldoYu, HakkiLeal-Junior, ArnaldoYu, HanwenLebedev, Alexander AlexandrovichYu, HaofeiLebel, KarinaYu, Heejung (Korea University)Lebioda, MarcinYu, Heejung (Yeungnam University)Lecamp, LaurenceYu, HongkaiLeccese, FabioYu, HongyuLech, PiotrYu, HuaizheLech, RafalYu, HuangchaoLechuga, YolandaYu, HuapengLecieux, YannYu, JaehyungLecler, SylvainYu, JaesokLedger, PaulYu, JiayongLednev, VasilyYu, JingLednev, Vasily NYu, JinhaiLedo, AnaYu, KyoungsikLeduc, DamienYu, LianDongLeduc, DominiqueYu, LiangjiangLee, Boon GiinYu, ManzhuLee, Boon-GiinYu, MiaoLee, Byeong HaYu, QingminLee, Byung ChulYu, QingxuLee, ByunghunYu, QuanqingLee, ByungKwanYu, Ruei-SungLee, ChaiwooYu, RuozhouLee, ChanghoYu, ShiqiLee, ChanghoonYu, Son-cheolLee, Chang-HunYu, WeiLee, Chang-JuYu, WeizeLee, Chang-RyeolYu, WenwuLee, Chang-SooYu, XiaojunLee, ChangwonYu, YangLee, Chao-HsienYu, YufengLee, Chi HwanYu, YushuLee, Chong HyunYu, ZeyunLee, Choong HeonYuan, BingLee, DeukheeYuan, FeiLee, Eui ChulYuan, HuiLee, Gi-JaYuan, LeiLee, Gyu MyoungYuan, MingLee, GyudoYuan, QiangqiangLee, Hee-JoYuan, Shyan-MingLee, HoonsooYuan, WeijieLee, Hsiang-ChiehYuan, WenanLee, Hsiao-YiYuan, WenzhenLee, Hyeong JaeYuan, YeLee, Hyun HoYuan-Tsung, ChenLee, Hyung KeunYuce, MehmetLee, Hyunjoo J.Yudin, DmitryLee, HyunsooYue, JingLee, Il-GuYue, PengLee, In SooYue, ShigangLee, Jae OneYue, TaoLee, JaesungYue, WenzhenLee, Jae-WooYue, ZhangLee, JangmyungYuen, Ka-vengLee, Jea UkYuen, PeterLee, JieunYufera, AlbertoLee, JihoonYúfera, AlbertoLee, JinseokYUN, IL DONGLee, Jin-ShyanYun, Ji-HoonLee, JinsilYun, Jong PilLee, Jong TaekYun, YounghyunLee, JongyongYun, YoungsunLee, JooHoYunus, Ali P.LEE, JOON-YONGYunusa-Kaltungo, AkiluLee, Ju-HyuckYurekli, Ali I.Lee, JuhyunYuste-Delgado, Antonio JesusLee, Jung KeunYüzügüllü, OnurLee, Jung SeungZ. Psarakis, EmmanouilLee, Jung-RyunZaballos Diego, AgustínLee, JungshinZabierowski, WojciechLee, Jun-HakZabihi, OmidLee, JunseopZabini, FlavioLee, Ju-YiZaborowicz, MaciejLee, Keon MyungZabulis, XenophonLee, KeunhwaZacchini, LeonardoLee, Kwan HyiZacharatos, FilimonLee, Kyu-haengZachariades, ChristosLee, MeonghunZagajewski, BogdanLee, MinZagan, IonelLee, MoonjinZagar, BernhardLee, Myeong-jinZaghetto, AlexandreLee, Paul Tae-WooZaghloul, MohamedLee, PosenZaghloul, MonaLee, Sang HyunZago, MatteoLee, Sang JunZagrodny, BartłomiejLee, Sang WoonZaharia, GheorgheLee, Sang-MaeZahid, TaimoorLee, Sang-SunZahorian, Stephen A.Lee, SangukZaitouny, AyhamLee, Sang-WookZaitsev, Boris D.Lee, Sang-WoongZaitsev, Dmitry L.Lee, Seok JaeZaitsu, Shin-ichiLee, SeulkiZajc, MatejLee, Seung JuZakaria, AmerLee, SeungchulZakšek, KlemenLee, SeungyunZaleski, StephanieLee, Shie-JueZalewski, PawelLee, Soo SukZam, AzharLee, SoojeongZamani, MazdakLee, Suk JinZamfir, Lucian-GabrielLee, SukhoZamfira, Constantin SorinLee, Sung PilZaminpardaz, SafooraLee, SunghoŻamojć, KrzysztofLee, SungjuZamora-Polo, FranciscoLee, SungonZampieri, NicolòLee, Tae YoonZanchetta, GiulianoLee, UichinZang, YapingLee, Wearable DeviceJinseokZangeneh-Nejad, FarzadLee, WinsonZangl, HubertLee, Won ChulZanotti Fragonara, LucaLee, WoncheolZanutta, AntonioLee, YangsunZapata, Jaime W.Lee, YoncheolZapłata, JacekLee, Yong-HwanZappatore, MarcoLee, Yoon HoZappone, AlessioLee, YoungDooZarayeneh, NedaLee, YounhoZarazaga, F. JavierLeeke, MatthewZareei, MahdiLefebvre, GrégoireZarejousheghani, MashaalahLefèvre, EricZarembski, AllanLegat, AndražZarepoor, MasoudLeger, Jean-MichelZarifi, Mohammad H. (University of Alberta)Legleiter, Carl J.Zarifi, Mohammad H. (University of British Columbia)Legutko, StanisławZarpalas, DimitriosLe-hegarat, SylvieZarpelão, BrunoLehmann, ArminZarpelão, Bruno BogazLehmann, RüdigerZarra, TizianoLei, ChongZarrin, HadisLei, DengZarzar, Lauren D.Lei, HongjiangZatelli, PaoloLei, ManchunZavabeti, AliLei, MeiZavafer, AlonsoLei, Po-Ruey (Barry)Zawadzki, PrzemysławLei, XushengZayas, Almudena DiazLei, ZhouZboril, FrantisekLeightley, DanielZednik, RicardoLeineweber, MatthewZednikova, MariaLeinonen, JussiZefferer, ThomasLeinonen, MarkusZegzhda, PeterLeira, Bernt J.Zeid, AbeLeister, WolfgangZeimpekis, IoannisLeitao, Diana C.Zeinali, YashaLeitner, GerhardZelei, AmbrusLekunberri, Edorta CarrascalZema, Nicola RobertoLeliaert, JonathanZeman, KrystofLemaire, EdwardZemcik, PavelLemic, FilipZemková, ErikaLemma, EnricoZemouri, RyadLemnaru, CameliaZeng, FengLemon, ChristopherZeng, HulieLenac, KristijanZeng, JiangyuanLenci, StefanoZeng, Jun-JieLeng, JieWuZeng, PengLengden, MichaelZeng, ShuwenLenik, JoannaZeng, XiangyanLenoir, MatthieuZeng, YongLensu, MikkoZeng, Zhao-ChengLenton, GavinZeng, ZhiguoLenz, ChristophZeng, ZhoumoLeo, MarcoZeni, LuigiLeón, OlgaZennaro, MarcoLeone, PierreZeppelzauer, MatthiasLeong, ZhaoyuanZetik, RudolfLeonow, SebastianZeydan, EnginLeonowicz, ZbigniewZhai, ChaoLeontidis, GeorgiosZhan, GaoLepine, JulienZhan, WeiLepouras, GeorgiosZhang, A PingLeppakoski, HelenaZhang, AronLeppäkoski, HelenaZhang, BaochangLera, FranciscoZhang, BaochengLerche, ChristophZhang, BinLerga, JonatanZhang, BingLerma, ClaudiaZhang, ChaoLeroux, DenisZhang, ChengLeroux, PaulZhang, ChengyuanLeroux, PhilipZhang, ChiLerro, AngeloZhang, ChuanLesiak, PiotrZhang, CongchunLesniewska, DanutaZhang, DapengLete, CeciliaZhang, Di (Korea)Letia, TiberiuZhang, Di (USA)Lettieri, StefanoZhang, DimingLetu, HusiZhang, DonglaiLeucci, GiovanniZhang, DongmingLeung, CarsonZhang, DouLeung, Kwong-SakZhang, Dr. YushuLeutenegger, MarcelZhang, Eric J.Leuzzi, FabioZhang, Fan (Beijing University of Chemical Technology)Lev, DanZhang, Fan (Chinese Academy of Sciences)Leveugle, RégisZhang, FangzhaoLevin, EugeneZhang, FangzhengLevski, DeyanZhang, FeihuLevy, Jason K.Zhang, FuminLewandowicz, ElżbietaZhang, GuangmingLewińska, PaulinaZhang, GuimingLewis, ElfedZhang, Gui-RongLewis, GregoryZhang, GuogangLewis, PeterZhang, GuojunLeyva, RobertoZhang, GuoqingLeyva-Mayorga, IsraelZhang, GuozhiLezzerini, MarcoZhang, HaiLhotska, LenkaZhang, Haichong K.Li, BaoqingZhang, HaihongLi, BeiwenZhang, HaijunLi, BinZhang, HaiyanLi, BingZhang, Han (Singapore)Li, BinghaoZhang, Han (USA)Li, BoZhang, HaoLi, BofengZhang, HaopengLi, ChangqingZhang, HeLi, ChenZhang, HengcaiLi, ChengdongZhang, HepingLi, ChengqingZhang, HeyeLi, ChongZhang, HongLi, ChongyiZhang, HongboLi, ChunguoZhang, HongpengLi, ChunxuZhang, HongyanLi, DavidZhang, HongyingLi, De-junZhang, HuihuiLi, DeminZhang, HuilanLi, DezhiZhang, IvanLi, DianqingZhang, JianLi, DongZhang, Jian AndrewLi, DongshengZhang, JianmingLi, DuoZhang, Jianzhong (Harbin Engineering University)Li, FanZhang, Jianzhong (Southeast University)Li, FeiZhang, Jianzhong (Taiyuan University of Technology)Li, FengqiangZhang, JiashuLi, FuZhang, JiazhongLi, FuhuaZhang, JieLi, GangZhang, JingjingLi, GenZhang, JingxiongLi, GuofaZhang, JinnanLi, GuoqiangZhang, JixianLi, GuyueZhang, JulieLi, HaijunZhang, JunqingLi, Hanchuanzhang, kuangLi, HaoZhang, LameiLi, HengchaoZhang, LanLi, Heng-ChaoZhang, LefeiLi, HongZhang, Lei (Australia)Li, Hong XianZhang, Lei (China)Li, HongkunZhang, Lei (University of Maryland, USA)Li, HongqiangZhang, Lei (UT-Dallas, USA)Li, HuanZhang, Leo YuLi, JiaZhang, LiangLi, JianZhang, LibaoLi, JiangZhang, LibingLi, JianhuaZhang, LifuLi, JianyuZhang, LiguoLi, JiayuZhang, LihaiLi, JinZhang, LijunLi, JingZhang, Lin (Henan University, China)Li, Jing SongZhang, Lin (Yangtze Normal University, China)Li, JinghuaZhang, LiqiangLi, JiweiZhang, LiqunLi, JunZhang, LiyanLi, JunhuiZhang, LuLi, KezhiZhang, MiaoLi, Ki-JouneZhang, MingLi, KuiZhang, MingzheLi, LeileiZhang, PengfeiLi, LiZhang, PenglinLi, LiangqunZhang, QiLi, LiangzhiZhang, QieshiLi, LinZhang, QijinLi, Ling-YunZhang, QingLi, LinyiZhang, QingjunLi, LiqiangZhang, QuanLi, LiweiZhang, QunLi, LiyuanZhang, RongLi, LongjiangZhang, RonghuiLi, MeichengZhang, Rui (China)Li, MengZhang, Rui (Germany)Li, MiaoZhang, ShaojieLi, MinZhang, ShaoliangLi, MingZhang, ShaolinLi, MingchaoZhang, ShengpingLi, MingchuZhang, ShijieLi, MinghuiZhang, ShimingLi, MoZhang, ShiwuLi, NaZhang, ShuaiLi, NanZhang, ShulingLi, PanZhang, SuLi, PeijunZhang, Sung-UkLi, PengdaZhang, TaoLi, QianZhang, TianweiLi, QiliangZhang, TingtingLi, QingZhang, WanchangLi, QingyongZhang, Wei (CN)Li, RongpengZhang, Wei (Singapore)Li, RuiZhang, WeidongLi, RuozhouZhang, WeijieLi, RuyaZhang, WeixingLi, ShandongZhang, WeizheLi, ShaopengZhang, WenboLi, ShengZhang, WenjunLi, ShiyangZhang, Wenjun (Chris)Li, ShuaiZhang, WentaoLi, ShuoZhang, WenyuLi, TaiyongZhang, WenzengLi, TaoZhang, WuxiongLi, TengZhang, XiandouLi, TianchengZhang, Xian-MingLi, TianliangZhang, Xiao (UK)Li, WanwanZhang, Xiao (USA)Li, WeiZhang, XiaochenLi, Wei-ChangZhang, XiaodongLi, WeijieZhang, XiaofeiLi, WeiqiangZhang, XiaogangLi, WenkaiZhang, XiaoshengLi, WenliangZhang, Xiao-YuLi, WenlingZhang, XinMingLi, Wen-TaiZhang, XinxinLi, WenwuZhang, XiuyingLi, XiZhang, XizhengLi, XiangZhang, XuechenLi, XianjuZhang, XuefengLi, Xiao JianZhang, Xun (Beijing Technology and Business University)Li, XiaodongZhang, Xun (Southwest Jiaotong University)Li, XiaofangZhang, Yan (Rockee)Li, XiaoluZhang, Ya-nanLi, XiaopengZhang, YanpingLi, XiaoqiangZhang, YanshunLi, XinZhang, YeLi, XinbinZhang, YiLi, XinghuaZhang, YimingLi, XingxingZhang, YinLi, XinmingZhang, YinghuiLi, XinyuZhang, YongboLi, XinzhongZhang, YongcunLi, XishengZhang, Yu (Stanford University)Li, XiuZhang, Yu (UK)Li, XuanZhang, Yu (University of Vermont)Li, XudongZhang, YuanyuLi, XuejinZhang, YuanzhiLi, XuekeZhang, YudongLi, XujieZhang, YunLi, XuranZhang, YunfeiLi, YangfanZhang, YunfengLi, YanshengZhang, YutingLi, YantaoZhang, ZhanyingLi, YiZhang, ZhenbinLi, YinZhang, ZhengLi, YingZhang, ZhenjiangLi, Ying-ChunZhang, ZhiguoLi, YingguangZhang, ZhiqiangLi, YingsongZhang, ZhongbaoLi, YinyaZhang, Zhou (New York City College of Technology)Li, YongZhang, Zhou (University of Wisconsin-Madison)Li, YongboZhang, ZonghuaLi, YouZhang, ZutaoLi, YuanZhao, BaoliangLi, YuandongZhao, ChaoyingLi, YuanhaoZhao, ChengLi, YuhangZhao, ChumingLi, YulongZhao, ChunhuiLi, YundongZhao, DiLi, Yung-HuiZhao, DongfangLi, YunhuaZhao, DonglinLi, YuwenZhao, FeipingLi, YuxingZhao, GangLi, ZheZhao, GenpingLi, ZhengZhao, Guo-huaLi, Zheng (Eddie)Zhao, HaifengLi, ZhenglinZhao, HangLi, ZhenguoZhao, HengjunLi, ZhengweiZhao, HongLi, ZhenhuaZhao, HongyuLi, ZhibinZhao, HuichanLi, ZhihuaZhao, JianLi, ZhijieZhao, JingzhouLi, ZhixiongZhao, JinlingLi, ZhiyuanZhao, JinpingLi, ZishenZhao, JinqiLi, ZuohuaZhao, JinsongLian, Ian Y.Z.Zhao, KaichunLian, JieZhao, LiboLian, XizhenZhao, LijunLiang, ChengchaoZhao, LinpingLiang, Chiu-kuoZhao, MingLiang, CunrenZhao, NanLIANG, DongZhao, PeixinLiang, GuolongZhao, PengxiangLiang, Hai NingZhao, QibinLiang, HuaweiZhao, QijunLiang, JinxingZhao, RuiLiang, KaitaiZhao, SichengLiang, LiangZhao, SihaoLiang, LinzhouZhao, TianLiang, Tsair-ChunZhao, TiebiaoLiang, XiaoZhao, WeiLiang, YanZhao, WeijieLiang, YiZhao, WeiqianLiang, YunjiZhao, WeishengLiang, ZiluZhao, WeiweiLiao, Shu-HsienZhao, WenfengLiao, Tai-ShanZhao, XiaoyuLiao, Teh-LuZhao, XihongLiao, Tien-HaoZhao, Yang (Canada)Liao, Wen-IZhao, Yang (USA)Liao, XinqinZhao, YanliLiao, Ying-ChihZhao, YanyuLichti, DerekZhao, YifanLicitra, GaetanoZhao, YiliangLie, ArneZhao, YongqiangLieberzeit, Peter AZhao, YubinLiehr, SaschaZhao, ZhanLienhart, WernerZhen, DongLiesenberg, VeraldoZhen, ZhenLigęza, PawełZheng, BoLiKamWa, RobertZheng, ChengshiLillethun, DaveZheng, DongLim, DaewoonZheng, DonghaiLim, Deok WonZheng, GuoanLIM, GilbertZheng, Hai-TaoLim, Jae-HanZheng, JiangbinLim, KihoZheng, JunLim, Kok SingZheng, LeLim, Kwan HuiZheng, Lian YuLim, Min-CheolZheng, LiangLim, SamsungZheng, LingxiangLim, SolZheng, PaiLim, SungjoonZheng, WenboLim, Yee YanZheng, XiLima, FredericoZheng, XianweiLima, José LuisZheng, XiaojingLima, Rui A.Zheng, XintingLimbeck, AndreasZheng, Xu-QianLimiti, ErnestoZheng, YangongLimongelli, Maria GiuseppinaZheng, YiliLin, Bor-ShingZheng, YiqunLin, ChengZheng, YuanjinLin, Cheng-HungZhong, BoLin, ChengyouZhong, CaimingLin, Che-WeiZhong, RuofeiLin, Chia-FengZhong, ShuncongLin, Chien-hongZhong, Yu LinLin, Chih-HongZhong, YuanhongLin, Chih-mingZhong, YuchengLin, Chih-PingZhong, ZhangduiLin, Chih-TingZhou, AoLin, Chin E.Zhou, BaodingLin, Chin-FengZhou, BingpengLin, ChingShunZhou, BingpuLin, Chi-YiZhou, BoLin, Chung-WeiZhou, ChengweiLin, Chun-HungZhou, ChuanboLin, ChunyuZhou, ChunjieLin, Der-YuhZhou, CimingLin, FengZhou, FengLin, Feng-ChengZhou, FengfengLin, GrayZhou, FuhuiLin, GuoqingZhou, GuoqingLin, Guo-ShiangZhou, HaiboLin, HongZhou, HuachunLin, Hsueh-ChunZhou, HuanLin, Huei-YungZhou, HuapengLin, HuiZhou, HuiyuLin, Hui-TingZhou, JiafengLin, HungyenZhou, JianfengLin, JerryZhou, JianhuaLin, JianhanZhou, JianjiangLin, John Chun-HanZhou, JiehanLin, JunZhou, JinLin, Kun-WeiZhou, JinghaiLin, Kuo-YiZhou, LianjieLin, MengZhou, MengchuLin, MengshiZhou, MingLin, Ming-BoZhou, NanLin, MingyueZhou, QiangLin, Pao TaiZhou, RongLin, Shih-ChunZhou, SusanLin, Tang-HuangZhou, WenjunLin, TingtingZhou, WenyaLin, Tsung-HungZhou, XianlianLin, Tzu-EnZhou, Xiao DongLin, WeiZhou, XiaoboLin, WenZhou, XiaopingLin, Wen-YenZhou, XiranLin, XiaoshanZhou, YapingLin, YangZhou, YongquanLin, Yang-WeiZhou, YufengLin, YaolinZhou, Yunlai (Singapore)Lin, Yuan-PinZhou, Yunlai (Spain)Lin, Yuh-ChungZhou, ZebingLin, Yu-HsiuZhou, ZeboLin, ZekaiZhou, ZhenyuLin, ZhibinZhou, ZhiqiangLin, Zuan-TaoZhou, ZimuLinden, MariaZhu, BenpengLindner, GerhardZhu, BinhaiLindner, LarsZhu, BowenLindquist, NathanZhu, ChenxinLing, BingoZhu, ChunshengLing, BoZhu, CongxuLing, QiangZhu, FanLing, SteveZhu, GuohunLingua, AndreaZhu, HuachengLinker, RaphaelZhu, JunxiaoLinnhoff-Popien, ClaudiaZhu, LiangdongLins, Romulo GoncalvesZhu, LingliLins, Vanessa De Freitas CunhaZhu, LiqiangLinsen, LarsZhu, NiLinß, SebastianZhu, QiuxiLinzon, YoavZhu, XiLioe, De XingZhu, XiaofengLiolios, Konstantinos A.Zhu, XiaoliangLiolis, KonstantinosZhu, XiaolinLiotou, EiriniZhu, XiufangLiotta, AntonioZhu, XuanLiou, MichelleZhu, Xu-DongLipiński, AdamZhu, XuejunLipiński, SewerynZhu, YaguangLipnickas, ArunasZhu, YanminLisboa, Adriano ChavesZhu, YiboLisein, JonathanZhu, YongLisi, MatteoZhu, YongxuLisiński, PrzemysławZhu, YungangLisjak, DragutinZhu, YunpengLiska, JindrichZhu, ZhigangLisowski, JózefZhu, ZhiyuanLissenden, CliffZhu, ZijieLissorgues, GaelleZhuang, ChijieListanti, MarcoZhuang, HanqiLittle, ThomasZhuang, LinaLitwin, DariuszZhuang, WeichaoLiu, Alan Z.Zhuang, XinLiu, AnfengZhuang, YanLiu, BifengZhuang, YuanLiu, BoZhuo, XiangyuLiu, ChangZhuo, YueLiu, ChangqingZhuravlev, AndreyLiu, ChangrongZi, BinLiu, ChaoZi, YunlongLiu, ChaoxingZia, Muhammad Yousuf IrfanLiu, ChengyuŽic, MarkLiu, Chien-HaoZidek, KamilLiu, Chien-ShengZiębiński, AdamLiu, ChuanjunZiebold, RalfLiu, Chung-ChiunZielinski, OliverLiu, ChuntaoZiemianski, LeonardLiu, Damon Shing-MinZietkiewicz, Joannaliu, DatongZíková, NaděždaLiu, DongZikria, Yousaf BinLiu, EnjieZilic, ZeljkoLiu, ErfuZilinskas, AntanasLiu, FaguiZillger, TinoLiu, FanZima, BeataLiu, FangZimmer, AlessandroLiu, FangmingZimmermann, FelixLiu, FeiZin, Thi ThiLiu, GangjunZink, Matthias DanielLiu, GuigenZinn, Yuri L.Liu, GuixiongZiogou, ChrysovalantouLiu, GuochengZiółkowski, PatrykLiu, GuoliangZiolkowski, RobertLiu, HaitaoZissis, GeorgesLiu, HaiyunZitouna-Chebbi, RimLIU, HexuZivcak, MarekLiu, HongboZłotkowska, DagmaraLiu, HongchaoZobi, FabioLiu, Hsiao-ChuanZohrabi, NasibehLiu, HuaishanZoitl, AloisLiu, HuanZÖLDY, MátéLiu, HuiZolfagharian, AliLiu, HuizengZoli, MarcoLiu, Hung-HuanZolotukhin, MikhailLiu, Jain-ShingZorbas, DimitriosLiu, Jen-ChiehZormpas, DimitriosLiu, JialunZorzano, María-PazLiu, JianZosel, JensLiu, JianguoZotti, AldobenedettoLiu, JianhuaZou, BinLiu, JianlinZou, DujianLiu, JianqiZou, FangxinLiu, JianzhengZou, HaiyangLiu, JiechaoZou, HangLiu, JingZou, JieLiu, JingfeiZou, JunLiu, Jing-SinZou, LeiLiu, JinjieZou, QinLiu, JuewenZou, TingLiu, JunZou, XiangjunLiu, JunminZou, XiaoboLiu, KaiZou, XudongLiu, KefeiZou, YajieLiu, Keng-KuZou, YoushengLiu, Kuang-YenZouhar, AlexanderLiu, KunZovko, MonikaLiu, LanjunZsoldos, RebekaLiu, LiZubarev, AndreyLiu, LiangguiZubizarreta, AsierLiu, LianxiZucca, FrancescoLiu, LinZuccon, PaoloLiu, LinboZuffada, CinziaLiu, LinfengZuffo, MichelaLiu, LingqiaoZuluaga, Maria A.Liu, MinZúñiga, EmmanuelLiu, MingZuniga, Marcos DLiu, MinminZuppella, PaolaLiu, NingZúquete, AndréLiu, PanZurbuchen, AdrianLiu, PeixiangZurita, GroverLiu, PengZvánovec, StanislavLiu, PengfeiZweiri, YahyaLiu, QiZwolak, KarolinaLiu, QiongZych, MarcinLiu, QuanhuaŻyczkowski, MarekLiu, QuanyingŻygierewicz, JarosławLiu, RanZygiridis, TheodorosLiu, RenΚaliviotis, Stathis

